# Cardiac myofibrillogenesis is spatiotemporally modulated by the molecular chaperone UNC45B

**DOI:** 10.1016/j.stemcr.2023.05.006

**Published:** 2023-06-08

**Authors:** Serena Huei-An Lu, Yi-Hsuan Wu, Liang-Yu Su, Zi-Ting Hsu, Tzu-Han Weng, Hsin-Yu Wang, Chiao Yu, Paul Wei-Che Hsu, Su-Yi Tsai

**Affiliations:** 1 Department of Life Science, National Taiwan University, Taipei 10617, Taiwan; 2 Research Center for Developmental Biology and Regenerative Medicine, National Taiwan University, Taipei 10617, Taiwan; 3 Genome and Systems Biology Degree Program, National Taiwan University, Taipei 10617, Taiwan; 4 Institute of Molecular and Genomic Medicine, National Health Research Institutes, Miaoli County 350, Taiwan

**Keywords:** Sarcomere, molecular chaperone, cardiac myofibrillogenesis, protocostamere, cardiomyocyte, CRISPR/Cas9 technique

## Abstract

Sarcomeres are fundamental to cardiac muscle contraction. Their impairment can elicit cardiomyopathies, leading causes of death worldwide. However, the molecular mechanism underlying sarcomere assembly remains obscure. We used human embryonic stem cell (hESC)-derived cardiomyocytes (CMs) to reveal stepwise spatiotemporal regulation of core cardiac myofibrillogenesis-associated proteins. We found that the molecular chaperone UNC45B is highly co-expressed with KINDLIN2 (KIND2), a marker of protocostameres, and later its distribution overlaps with that of muscle myosin MYH6. UNC45B-knockout CMs display essentially no contractility. Our phenotypic analyses further reveal that (1) binding of Z line anchor protein ACTN2 to protocostameres is perturbed because of impaired protocostamere formation, resulting in ACTN2 accumulation; (2) F-ACTIN polymerization is suppressed; and (3) MYH6 becomes degraded, so it cannot replace non-muscle myosin MYH10. Our mechanistic study demonstrates that UNC45B mediates protocostamere formation by regulating KIND2 expression. Thus, we show that UNC45B modulates cardiac myofibrillogenesis by interacting spatiotemporally with various proteins.

## Highlights

• A non-replated hESC-CM model elucidates the stepwise process of sarcomere assembly
• UNC45-knockout CMs impaired protocostamere formation
• UNC45-knockout CMs failure to polymerize F-ACTIN and MYH6 replacement defects
• UNC45B modulates cardiac myofibrillogenesis by interacting with various proteins

## Graphical abstract

**Figure:**
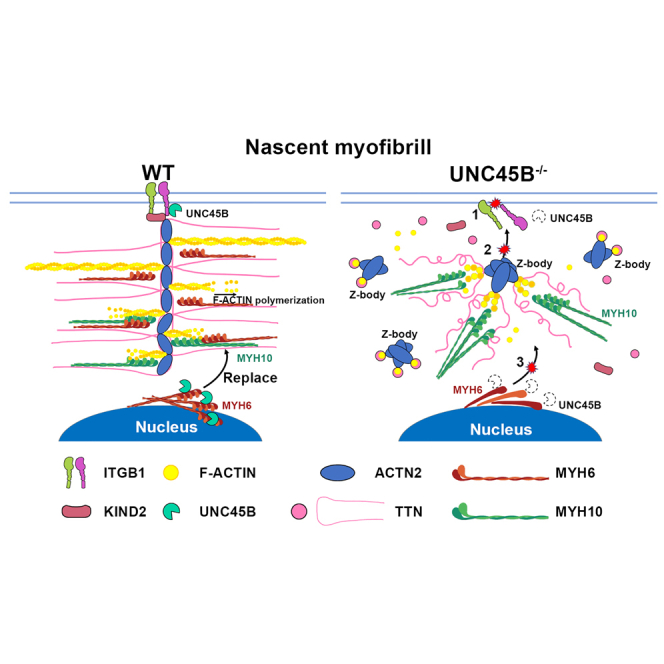

## Teaser

Lu et al. demonstrate that the molecular chaperone UNC45B modulates cardiac myofibrillogenesis by interacting spatiotemporally with various proteins in different cell components (protocostameres, non-muscle myosin, muscle myosin). By elucidating the stepwise process and spatiotemporal regulation of core cardiac myofibrillogenesis-associated proteins, they provide new insights into cardiac development and highlight potential new therapeutics for cardiomyopathies.

## Introduction

Assembled by hundreds of proteins, the sarcomere is the basic contractile unit of heart muscle (Clark et al., 2002). Each sarcomere is defined by two Z lines consisting of an A band (myosin thick filament) and an I band (actin thin filament). Mutations in genes encoding sarcomere-associated proteins result in cardiomyopathies, leading causes of morbidity and mortality worldwide. Although the function and organization of the sarcomere have been studied extensively, the precise process of sarcomere assembly remains elusive.

Among current models of sarcomere assembly, the premyofibril model is the widely accepted framework (Rhee et al., 1994). In this model, integrin adhesion sites, which form protocostameres (initial sites of sarcomerogenesis), recruit the Z line anchor protein ACTN2, actin, and non-muscle myosin (NMM) MYH10 for premyofibril assembly. During the transition from nascent myofibrils to mature myofibrils, muscle myosin II (MYH6) starts to displace NMM II B (MYH10), and Z bodies (composed of ACTN2, ACTIN, and TITIN [TTN]) amalgamate other Z bodies laterally into a Z line to form the mature sarcomere (Rhee et al., 1994; Sanger et al., 2010; Sparrow and Schock, 2009). Apart from the scaffold proteins that assemble into the building blocks of sarcomeres, the sarcomere assembly process is modulated by multiple regulatory factors, such as RNA-binding proteins (RBPs) and molecular chaperones (Poon et al., 2012; Srikakulam et al., 2008).

Our previous study demonstrated that the RBP RBM24 mediates alternative splicing of core myofibrillogenesis genes in a stage-specific manner and that ACTN2 interacts with the N terminus of TTN (TTN-N), allowing MYH6 to bind the C terminus of TTN (TTN-C) (Lu et al., 2022). Here, we examine how a molecular chaperone regulates sarcomere assembly. Uncoordinated mutant number 45 (Unc45) is a key molecular chaperone exerting a critical role in myosin assembly during sarcomerogenesis (Barral et al., 2002; Lee et al., 2011). Unc45 was first discovered in *Caenorhabditis elegans* (Epstein and Thomson, 1974; Venolia et al., 1999), and two isoforms (UNC45A and UNC45B) have been identified in mammals (Price et al., 2002); whereas UNC45A is expressed ubiquitously in all mammalian cells, UNC45B is expressed primarily in skeletal and cardiac striated muscle cells (Price et al., 2002). Myosin forms a complex with multiple chaperones when it assembles, including Unc45b and heat shock protein 90 (Hsp90) (Etard et al., 2007). Ultimately, myosin is incorporated into thick filaments, but Unc45b and Hsp90 both migrate to and remain at the Z line (Etard et al., 2008). Notably, under stress conditions, Unc45b and Hsp90 move to A bands, preventing myosin from denaturing (Etard et al., 2008). Additionally, ablation of *Unc45b* in mice elicited defective heart looping, and the mutant mice displayed embryonic lethality (Chen et al., 2012). *Unc45b* knockdown in zebrafish resulted in delayed nucleation of α-actinin and mislocalization of NMM (Myhre et al., 2014). Thus, UNC45B may exert functions in addition to modulating myosin folding during sarcomere assembly.

Human pluripotent stem cells (PSCs), including embryonic stem cells (ESCs) and induced PSCs (iPSCs), have the unique competence to differentiate into all cell types, including cardiomyocytes (CMs). Hence, human ESC (hESC)-derived CMs (hESC-CMs) provide great potential for studying sarcomere formation and cardiac development. Sarcomere assembly is a multi-step process, so we first used hESC-CMs to dissect the critical steps of the sarcomere assembly process using core myofibrillogenesis markers (ACTN2, MYH6, TTN, MYH10, and F-ACTIN), protocostamere markers (ITGB1 and KINDLIN2 [KIND2]), and a molecular chaperone (UNC45B). We established the following stepwise process of sarcomere assembly in our hESC-CM system: (1) protocostameres are initiated at directed cardiac differentiation day 4; (2) sarcomeres start to assemble and expression of muscle myosin MYH6 initiates at day 5, with premyofibrils being assembled by protocostameres, NMM, and Z bodies (ACTN2, TTN, and F-ACTIN); (3) at day 7 (the nascent myofibril stage), muscle myosin MYH6 replaces NMM MYH10 and completely co-localizes with TTN-C, with F-ACTIN also beginning to polymerize into actin thin filaments; and (4) at day 10, Z lines are established, and MYH6 fully replaces MYH10, thus completing the sarcomere assembly process.

Significantly, during the sarcomere assembly process, we found that UNC45B co-localizes with KIND2, MYH10, and MYH6 at different stages of sarcomere assembly. We sought to determine the function of *UNC45B*, so we ablated *UNC45B* from MYH6:mCherry cardiac reporter hESCs using the CRISPR-Cas9 technique. Interestingly, although hESCs with homozygous ablation of UNC45B (UNC45B^−/−^) still differentiated into CMs, essentially no beating contractility was detectable in the mCherry-positive cells. Phenotypic analysis further revealed ACTN2 accumulation because of impaired protocostamere formation, failure to polymerize F-ACTIN, and MYH6 replacement defects in the mutant cell lines. Notably, our mechanistic study demonstrates that UNC45B facilitates expression of the protocostamere marker KIND2, with disruption of *UNC45B* resulting in impairment of the initiating site of sarcomere assembly. Thus, UNC45B directs human cardiac myofibrillogenesis by spatiotemporally modulating protocostamere formation and muscle myosin folding. In conclusion, we have used directed cardiac differentiation to establish the spatiotemporal expression pattern of cardiac myofibrillogensis genes and the function of a molecular chaperon during the process of human sarcomere assembly.

## Results

### Deciphering the stepwise processes of cardiac myofibrillogenesis in hESC-derived CMs

**Figure 1:**
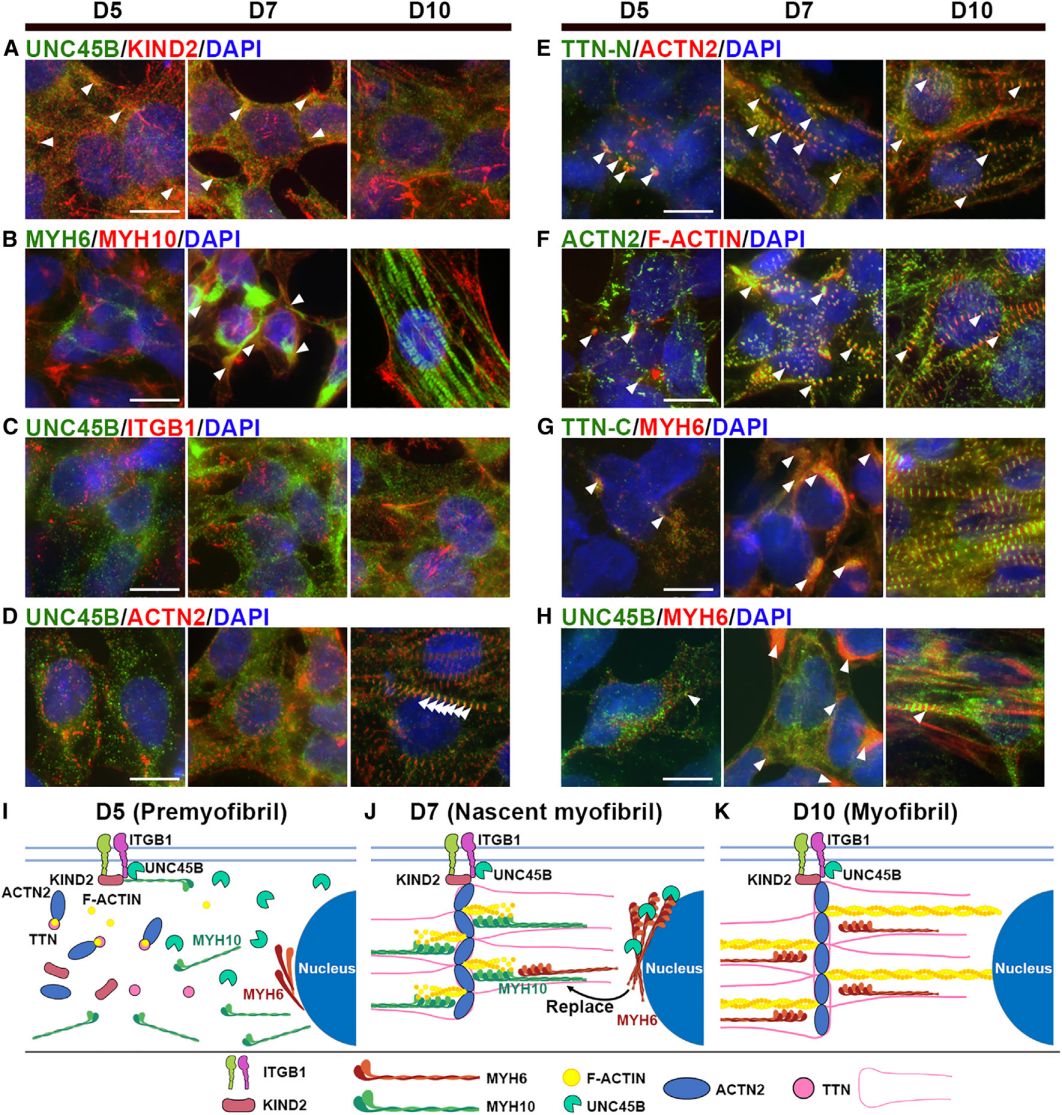
The stepwise process of cardiac myofibrillogenesis in human embryonic stem cell-derived cardiomyocytes (A–H) Representative immunofluorescence (IF) images of WT-CMs cultured on Matrigel-coated cover slides and harvested at the indicated time points. Cells were stained for (A) UNC45B and KIND2, (B) MYH6 and MYH10, (C) UNC45B and ITGB1, (D) UNC45B and ACTN2, (E) TTN-N and ACTN2, (F) ACTN2 and F-ACTIN, (G) TTN-C and MYH6, and (H) UNC45B and MYH6. Overlapping fluorescence signals in each panel are indicated by arrowheads. All fluorescence images have been merged with DAPI staining. Scale bars: 10 μm. (I–K) Schematics of sarcomere assembly and integrity at (I) day 5 (the premyofibril stage), (J) day 7 (nascent myofibril), and (K) day 10 (mature myofibril). Results represent data from at least three independent experiments. See also Figures S1 and S2.

To elucidate the stepwise process of human cardiac myofibrillogenesis, we cultured MYH6:mCherry cardiac reporter hESCs on Matrigel-coated cover slides and harvested hESC-CMs at differentiation days 5, 7, and 10 without replating, allowing us to observe the spatiotemporal process of sarcomere assembly. Immunofluorescence (IF) staining was conducted to examine the expression patterns of various markers for protocostameres (ITGB1 and KIND2), core myofibrillogenesis proteins (ACTN2, TTN, MYH6, MYH10, and F-ACTIN), and a molecule chaperone (UNC45B). Among these, MYH10, KIND2, ITGB1, and F-ACTIN can also be detected in non-cardiac cells. Expression of all cardiac-specific markers (UNC45B, ACTN2, TTN, and MYH6) we examined began on directed cardiac differentiation day 5 (Figures 1A–1H, S1A–S1H [unmerged images], and S2A–S2B), whereas NMM (MYH10) and the protocostamere marker KIND2 were co-localized on day 4 (Figure S2C), indicating that protocostameres form before other sarcomere components. Moreover, on day 5, we observed higher expression of UNC45B compared with other cardiac-specific markers (Figures 1D, 1H, S1D, and S1H). We also observed that on day 5, ACTN2 co-localized with TTN-N (stained with TTN-N-specific antibody) and actin thin filament (F-ACTIN), forming nucleation sites termed Z bodies (precursors of Z lines) (Figures 1E, 1F, and S2D). Furthermore, muscle myosin MYH6 co-localized with TTN-C (stained with TTN-C-specific antibody) and UNC45B (Figures 1G, 1H, S1G, and S1H). On day 7, MYH6 partially replaced NMM MYH10, so some MYH6-positive cells displayed signal overlap with MYH10 (Figure 1B, middle, and Figure S1B), and F-ACTIN began to polymerize into actin thin filaments (Figure 1F, middle, and Figure S1F). On day 10, MYH6 had fully replaced MYH10, with the former displaying a filamentous-like structure and the MYH10 solely being expressed at the cell periphery (Figure 1B, right panel, and Figure S1B). IF staining patterns for ACTN2, MYH6, and TTN were punctate-like (Figures 1D–1H and S1D–S1H), whereas those of MYH6, ACTN2, TTN, F-ACTIN, and UNC45B were filamentous-like (Figures 1B, 1D, 1E–1H, S1B, S1D, and S1E–S1H).

Intriguingly, IF staining revealed co-localization of UNC45B with the protocostamere marker KIND2 (Figures 1A and S1A), muscle myosin MYH6 (Figures 1H and S1H), as well as NMM MYH10 (Figure S2B). We also co-stained for the protocostamere marker KIND2 and both muscle myosin MYH6 and NMM MYH10. Notably KIND2 only co-localized with MYH10, and not with MYH6 (Figures S2A and S2C). Thus, UNC45B co-localizes with various sarcomeric components (protocostamere, muscle myosin, NMM). Taken together, our IF analysis reveals that (1) by directed cardiac differentiation day 4, NMM MYH10 has bound to protocostameres (labeled by KIND2); (2) sarcomere assembly begins at day 5, with UNC45B being present in the protocostamere and NMM forming earlier than other tested sarcomeric components (Figure 1I); (3) core myofibrillogenesis proteins (ACTN2, TTN, MYH6, and F-ACTIN) displayed a punctate pattern and low expression levels (Figure 1I); (4) ACTN2, together with TTN-N and actin thin filament (F-ACTIN), forms nucleation sites (i.e., Z bodies) (Figure S2D), with only a small proportion of muscle myosin MYH6 being bound to TTN-C and UNC45B (Figure 1I); (5) MYH6 starts to replace MYH10 by day 7, and F-ACTIN begins to polymerize into actin thin filaments (Figure 1J); and (6) by day 10, Z lines are established, and MYH6 has completely replaced MYH10, thereby completing the process of sarcomere assembly (Figure 1K).

### *UNC45B* knockout does not affect cardiac differentiation efficiency, but the resulting cells lack contractility

**Figure 2:**
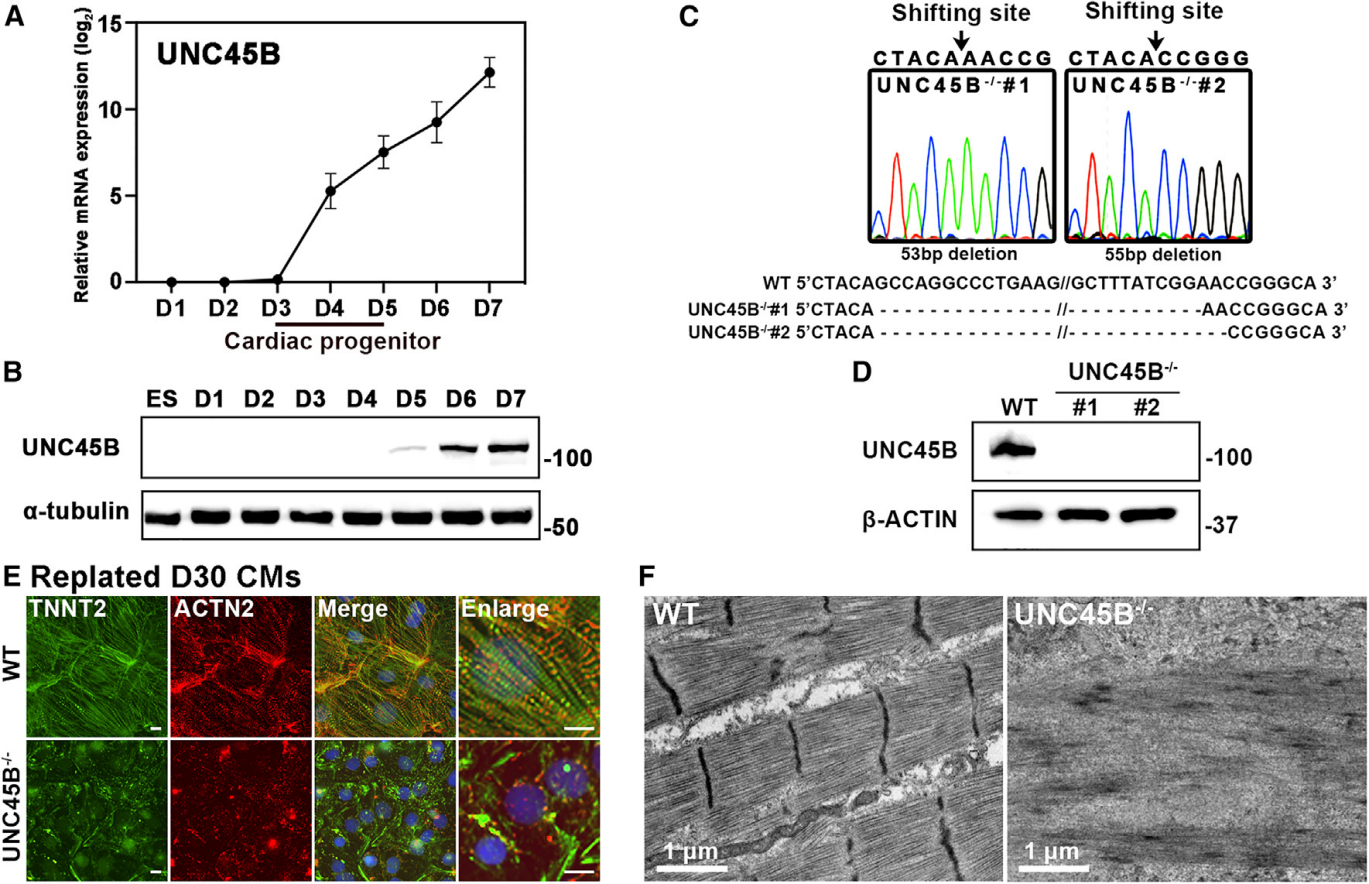
Ablation of *UNC45B* from hESCs (A and B) Transcription (A) and protein (B) levels of *UNC45B* in WT hESCs during directed cardiac differentiation from day 1 to day 7, as determined by qRT-PCR (n = 3) and western blot. Error bars represent SD. (C) DNA sequencing data of genomic DNA samples from the two homozygous *UNC45B*-knockout lines. Black arrows indicate the sgRNA cleavage sites. (D) Protein levels of UNC45B in WT-CMs and UNC45B^−/−^-CMs, as determined using western blot. (E) Representative IF images of replated WT-CMs and UNC45B^−/−^-CMs harvested at day 30 and stained for TNNT2 and ACTN2. Scale bars: 10 μm. (F) Representative TEM images of WT-CMs and UNC45B^−/−^-CMs. Scale bars are indicated. See also Figure S3.

Previous studies have shown that UNC45B plays a critical role in myosin assembly (Barral et al., 1998, 2002; Landsverk et al., 2007). However, we found that *UNC45B* expression began at the cardiac progenitor stage (UNC45B mRNA and protein were detected at days 4 and 5, respectively), and gradually increased at the CM stage (Figures 2A and 2B). Importantly, our IF analysis revealed that UNC45B is associated with different sarcomeric components (Figures 1A, 1D, 1H, S1A, S1D, S1H, and S2B). Thus, in order to determine if UNC45B conducts diverse functions in different components of the sarcomere, we ablated *UNC45B* from our previously published hESC cardiac reporter line (MYH6:mCherry) (Tsai et al., 2020) using a CRISPR-Cas9 approach. DNA sequencing further validated the deletion mutants. We selected two homozygous mutant lines harboring 53 or 55 bp deletions, respectively, for further study (Figure 2C). Western blot analysis revealed that UNC45B protein was essentially undetectable in both of these knockout lines (Figure 2D).

Next, we sought to assess if homozygous knockout of *UNC45B* (denoted UNC45B^−/−^) affected CM differentiation. We observed that UNC45B^−/−^ hESCs still differentiated into CMs and displayed MYH6:mCherry signals (Figure S3A). Flow cytometry analysis using TNNT2 staining combined with MYH6:mCherry signals further demonstrated a similar differentiation efficiency compared with a wild-type (WT) line (Figure S3B). However, whereas WT-CMs beat regularly, the UNC45B^−/−^-CMs did not exhibit any contractility (Video S1).

Video S1. Beating video of WT-CMs and UNC45B^-/-^-CMs

### UNC45B^−/−^-CMs exhibit severely disrupted sarcomeric structures

As UNC45B^−/−^-CMs could not contract, we investigated if the phenotype is caused by impairment of sarcomere structures. To do so, we conducted IF staining for ACTN2 (Z line marker) and TNNT2 (actin thin filament) on hESC-CMs at differentiation day 30. Notably, we observed disordered Z lines and actin thin filaments in our UNC45B^−/−^-CMs (Figure 2E). To further verify our IF results, we performed transmission electron microscopy (TEM) to visualize the sarcomeric structures of the hESC-CMs. Strikingly, in contrast to the well-organized sarcomere structures found in WT-CMs, UNC45B^−/−^-CMs displayed aberrant filamentous structures (Figure 2F), supporting that UNC45B modulates sarcomere organization and that blocking its activity inhibits cell contractility.

**Figure 3:**
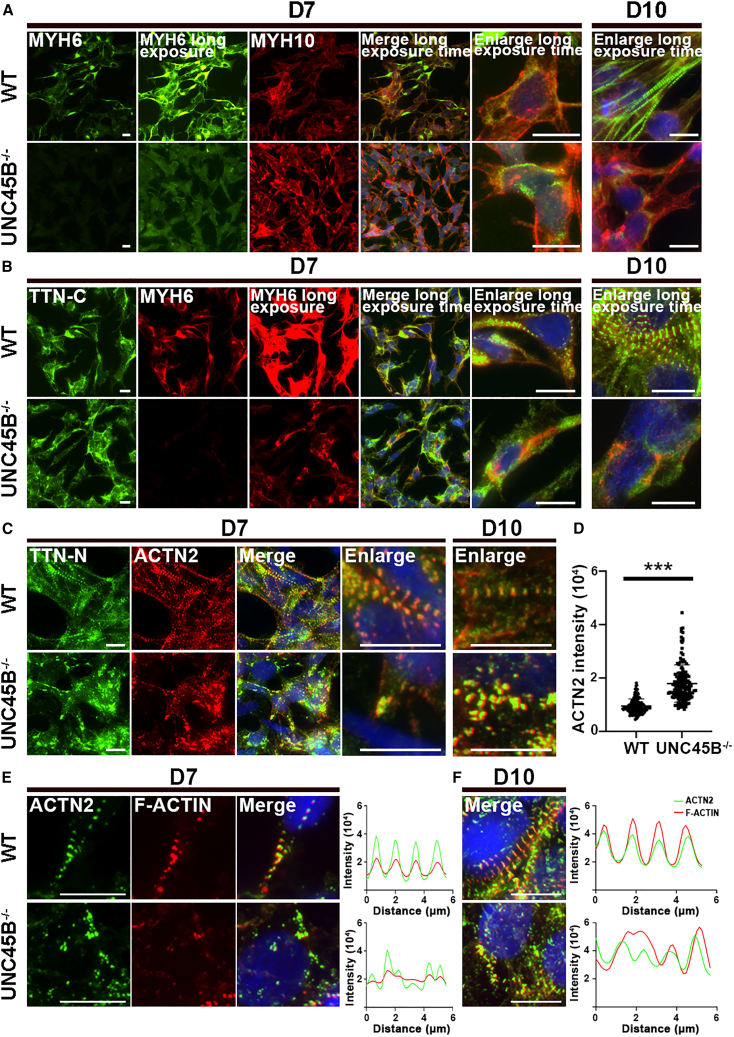
ACTN2 accumulation and defective F-ACTIN polymerization during the nascent myofibril stage in UNC45B^−/−^-CMs (A and B) Representative IF images of MYH6, MYH10, and TTN-C in non-replated WT-CMs and UNC45B^−/−^-CMs harvested at day 7 or day 10. Cells were stained for (A) MYH6 and MYH10 and (B) TTN-C and MYH6. To improve visualization of MYH6 signal in the UNC45B^−/−^ cells, additional sets of MYH6 images with a longer exposure time are shown. Scale bars: 10 μm. (C) Representative IF images of non-replated WT-CMs and UNC45B^−/−^-CMs harvested at day 7 or day 10 and stained for TTN-N and ACTN2. Scale bars: 10 μm. (D) Quantification results for ACTN2 signal intensity in WT-CMs and UNC45B^−/−^-CMs at day 7, showing ACTN2 accumulation (n = 150). Error bars represent SD. Statistical significance is indicated: ^∗∗∗^p < 0.001. (E and F) Left panel: representative IF images of WT-CMs and UNC45B^−/−^-CMs harvested at (E) day 7 and (F) day 10 and stained for ACTN2 and F-ACTIN. Scale bars: 10 μm. Results represent data from at least three independent experiments. See also Figure S3.

To further investigate how sarcomere assembly is affected by *UNC45B* ablation at early differentiation days 7 and 10 (representing the nascent myofibril and mature myofibril stages, respectively), we conducted IF staining for core myofibrillogenesis markers (MYH6, MYH10, ACTN2, TTN, and F-ACTIN). UNC45B is involved in regulating folding of the myosin head domain (Srikakulam et al., 2008). Our IF staining using anti-MYH6 antibody revealed that levels of MYH6 were substantially reduced in UNC45B^−/−^-CMs relative to WT (Figures 3A and 3B). Subsequently, we conducted western blot analysis to examine MYH6 protein expression. MYH6 levels were indeed significantly reduced in the UNC45B^−/−^-CMs at differentiation days 7 and 10 (Figure S3C). We examined if the unfolded MYH6 affects MYH10 replacement and TTN binding during sarcomere formation, potentially leading to the reduced MYH6 levels observed in the mutant cell lines. In WT-CMs, MYH6 co-localized with MYH10 at early cardiac differentiation day 7 (Figures 3A and S3D), with MYH6 displaying filamentous structures at day 10 when MYH10 signal substantially diminished (Figure 3A, upper panel). However, in the UNC45B^−/−^-CMs, most MYH6 did not co-localize with MYH10 at day 7 (Figures S3D and S3E), nor did it replace MYH10 (Figure S3D). Instead, in the mutant cells, MYH6 appeared to surround the nucleus at day 10 and did not form well-organized filaments (Figure 3A, lower panel, and Figure S3D). Western blotting analysis showed that amounts of MYH10 in fact increased in UNC45B^−/−^-CMs at day 15 (Figure S3F), indicating that *UNC45B* knockout causes myosin (MYH6) protein degradation, thereby limiting replacement of MYH10 by MYH6. Moreover, TTN-C and MYH6 co-localized and surrounded the nucleus of WT-CMs at day 7 (Figure 3B, upper panel, and Figures S3G and S3H), forming filamentous structures in those cells by day 10 (Figure 3B, upper panel, and Figure S3G). In contrast, MYH6 did not co-localize with TTN-C on days 7 or 10 in UNC45B^−/−^-CMs (Figure 3B, lower panel, and Figures S3G-H).

TTN-N and F-ACTIN can both bind to ACTN2, forming nucleation sites at day 5 (Figure S2D). As Z lines were severely disrupted in the UNC45B^−/−^-CMs, we examined if this phenotype is due to impaired nucleation site formation. Accordingly, we conducted IF staining to determine if ACTN2 co-localizes with TTN-N and F-ACTIN. Even though Z lines were perturbed in UNC45B^−/−^-CMs, ACTN2 still co-localized with TTN-N and F-ACTIN in both WT and UNC45B^−/−^-CMs (Figures 3C–3F and S3I). Notably, we observed an accumulation of ACTN2 (Figures 3C–3F), as well as a lack of filamentous-like actin thin filaments (Figures 3E and 3F, lower panel), in UNC45B^−/−^-CMs at days 7 and 10, indicating that the nucleation sites may not function properly. Thus, in addition to its role in myosin assembly, UNC45B most likely regulates Z line and actin filament formation.

In addition, as UNC45B is a molecule chaperon, it is most likely that UNC45B regulates these sarcomere markers (MYH6, MYH10, ACTN2, and KIND2) only in the protein level, but not their transcription. We therefore performed qPCR and corroborated that ablation of UNC45B did not affect the transcript levels of these tested sarcomere markers (Figure S4A).

### UNC45B regulates protocostamere formation as well as myosin degradation to modulate myofibrillogenesis

**Figure 4:**
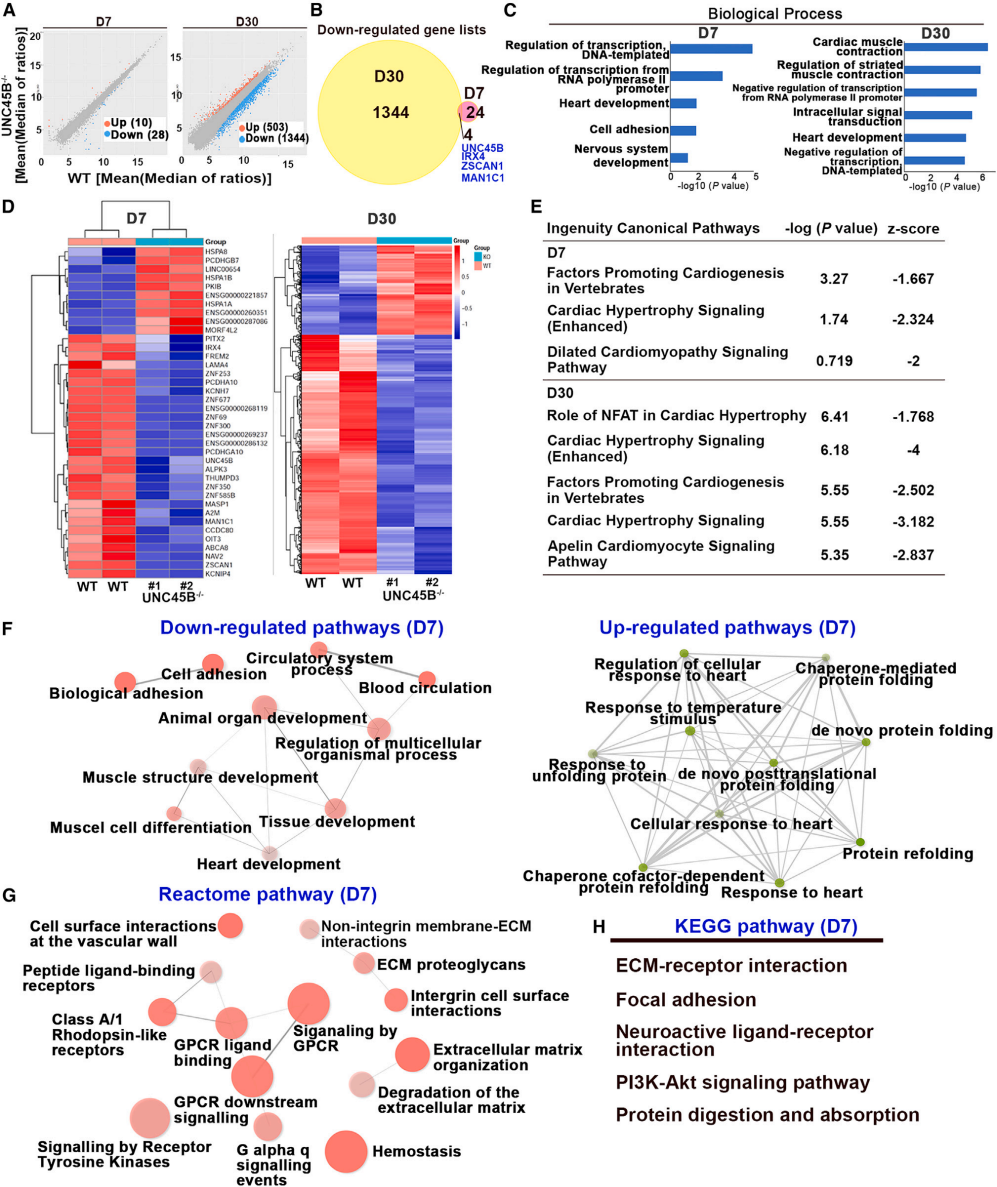
*UNC45B*-regulated genes mainly involved in heart development, extracellular matrix organization and focal adhesion (A) Scatterplot illustrating the DEGs based on RNA sequencing results from day 7 and 30 WT-CMs and UNC45B^−/−^-CMs. Up-regulated and down-regulated genes (adjusted p < 0.05) in UNC45B^−/−^-CMs relative to WT-CMs are shown in orange and blue, respectively. (B) Venn diagram analysis of UNC45B-regulated genes on the basis of day 7 (pink circle) and day 30 (yellow circle) RNA-seq datasets. (C) Gene Ontology (GO) biological process analyses of down-regulated genes in day 7 and day 30 UNC45B^−/−^-CMs. (D) Hierarchical clustering analysis of the DEGs from day 7 and day 30 RNA-seq datasets. Scale bar represents the *Z* score value. For RNA-seq, WT-CMs (n = 2), and UNC45B^−/−^-CMs (n = 2). (E) Ingenuity canonical pathway analysis of DEGs from day 7 and 30 RNA-seq datasets. Gene lists from up-regulated and down-regulated genes (p < 0.05) in UNC45B^−/−^-CMs relative to WT-CMs. (F) DEG enrichment network analysis of day 7 RNA-seq data reveals that these DEGs are involved in both down-regulated pathways and up-regulated pathways. (G and H) Reactome (G) and KEGG (H) pathway analysis of day 7 RNA-seq datasets. Note that gene lists for (E)–(H) are from fold change (F.C.) > 1.5 up-regulated and down-regulated genes (p < 0.05) in UNC45B^−/−^-CMs relative to WT-CMs. See also Figure S4.

We sought to elucidate the molecular mechanism by which UNC45B regulates sarcomere assembly. Hence, we isolated the RNAs of MYH6:mCherry-positive cells at differentiation day 7 and day 30 from both WT-CMs and UNC45B^−/−^-CMs, and then carried out RNA sequencing (RNA-seq). In Figure 4A, we present a scatterplot of genes either 1.5-fold up- or down-regulated (adjusted p < 0.05) in UNC45B^−/−^-CMs relative to WT-CMs. We detected 503 and 1344 genes that are either up- or down-regulated by *UNC45B* at day 30, respectively. However, much fewer genes were affected by *UNC45B* at early-stage day 7 (28 down-regulated and 10 up-regulated genes). We further performed Venn diagram analysis on those day 7 and day 30 down-regulated gene lists and found that four genes (*UNC45B*, *IRX4*, *ZSCAN1*, and *MAN1C1*) were down-regulated at both stages (Figure 4B). Gene Ontology (GO) analysis revealed that these down-regulated genes are involved in heart development in both day 7 and day 30 UNC45B^−/−^-CMs (Figure 4C). Additionally, day 7 down-regulated genes are also involved in regulation of transcript from RNA polymerase II, heart development, and cell adhesion (Figure 4C, left panel), whereas day 30 down-regulated genes are predominantly involved in cardiac muscle contraction, intracellular signal transduction, the Wnt signaling pathway, and regulation of striated muscle contraction, among other terms (Figure 4C, right panel). Next, to further validate the RNA-seq data, we selected multiple genes from different GO categories of day 7 RNA-seq and examined them using qPCR. These genes include (1) heart development (*FREM2*, *ALPK3*, *IRX4*), (2) cell adhesion (*FREM2*, *LAMA4*, *PCDHGA10*, and *PCDHA10*), and (3) regulation of transcript from RNA polymerase II promoter (*IRX4* and *PITX4*). Consistently, these selected genes are significantly down-regulated in day 7 UNC45B^−/−^-CMs (Figure S4B). Moreover, the heatmap in Figure 4D summarizes that *UNC45B* deletion mainly causes the majority of affected genes to be down-regulated. To further understand the UNC45B regulatory network at the early differentiation stage, we selected genes that are 1.5-fold up- and down-regulated (p < 0.05) from our day 7 and day 30 RNA-seq datasets and performed Ingenuity Pathway Analysis (IPA) (Figure 4E). Interestingly, we found that UNC45B-regulated genes in both day 7 and day 30 UNC45B^−/−^-CMs are involved in (1) factors that promote cardiogenesis in vertebrates and (2) signaling pathways related to cardiac hypertrophy and dilated cardiomyopathy (Figure 4E). In addition to cardiac-related pathways, UNC45B-regulated genes (day 7) also participate in the GP6 signaling pathway, cAMP-mediated signaling, HIF1 signaling, and the BAG2 signaling pathway, among others (Table S2). Additionally, gene enrichment network analysis on day 7 differentially expressed genes (DEGs) further revealed that these DEGs are involved in both down-regulated pathways (e.g., biological and cell adhesion, circulatory system process, muscle structure development, and heart development) (Figure 4F, left panel) and up-regulated pathways (e.g., chaperone-mediated protein folding, response to unfolded protein, and protein refolding) (Figure 4F, right panel). Moreover, Kyoto Encyclopedia of Genes and Genomes (KEGG) enrichment network analysis and reactome pathway analysis uncovered that these DEGs (day 7) are involved in extracellular matrix organization, focal adhesion, GPCR signaling, and integrin cell surface interactions (Figures 4G and 4H). Taken together, these gene regulatory networks corroborate that UNC45B is involved in cardiogenesis and the extracellular cell matrix (focal adhesion), consistent with the protocostamere phenotypes displayed by CMs upon UNC45B knockout.

**Figure 5:**
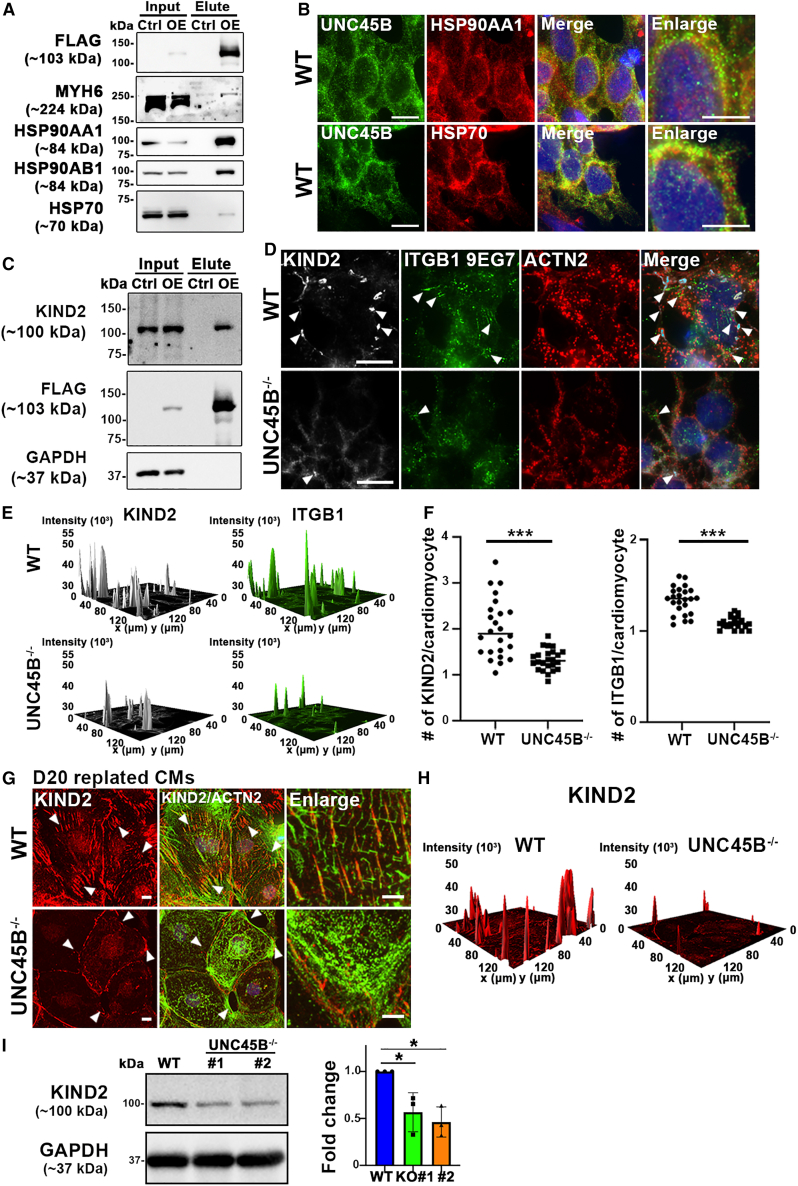
UNC45B modulates protocostamere formation by regulating KIND2 expression (A) Co-IP assay showing the association of UNC45B-FLAG with MYH6 and heat shock proteins, as detected using western blots. Cell lysates of WT (negative control) and WT^UNC45B-OE^-CMs (input) and pull-down samples using anti-FLAG beads (elute) were analyzed using SDS-PAGE. Anti-FLAG antibody was used to validate the co-IP assay. WT-CMs acted as a negative control. (B) Representative IF images of WT-CMs harvested at day 7 and stained with antibodies for UNC45B and HSP90AA1 or HSP70. Scale bars: 10 μm. Enlargement scale bars: 5 μm. Results represent data from at least three independent experiments. (C) Western blot following UNC45B-FLAG and KIND2 pull-down assay. (D) Representative IF images of WT-CMs and UNC45B^−/−^-CMs harvested at day 7 and stained for KIND2, ITGB1 9EG7, and ACTN2. Nucleation sites are indicated by arrowheads. Scale bars: 10 µm. (E) Representative fluorescence intensity three-dimensional (3D) surface plots of KIND2 and ITGB1 9EG7 signals in day7 WT-CMs and UNC45B^−/−^ CMs, illustrating protocostamere sites. (F) Quantification results of numbers of KIND2 and ITGB1 nucleation sites per CM in WT-CMs and UNC45B^−/−^-CMs (KIND2, n = 24; ITGB1, n = 24). (G) Representative IF images of replated WT-CMs and UNC45B^−/−^-CMs at day 20 and stained for KIND2 and ACTN2. Protocostamere sites are indicated by arrowheads. Scale bars: 10 μm. Enlargement scale bars: 5 μm. (H) Representative fluorescence intensity 3D surface plots of KIND2 signal in replated WT-CMs and UNC45B^−/−^-CMs at day 20. (I) Western blots and the corresponding quantification data for KIND2 signal in WT-CMs and UNC45B^−/−^-CMs at day 20. Error bars represent SD. Statistical significance is indicated: ^∗^p < 0.05 and ^∗∗∗^p < 0.001. Results represent data from at least three independent experiments. See also Figure S5.

Next, to determine the binding partners of UNC45B during sarcomere assembly, we conducted co-immunoprecipitation (co-IP) coupled with mass spectrometry (MS). Among them were a number of well-known UNC45B binding partners (e.g., MYH6, HSP90AA1, HSP90AB1 and HSP70) (Table S3), which were verified using western blotting (Figure 5A), thereby validating our co-IP/MS analysis. IF staining further confirmed co-localization of UNC45B with MYH6, HSP90AA1, and HSP70 (Figures 1H and 5B).

**Figure 6:**
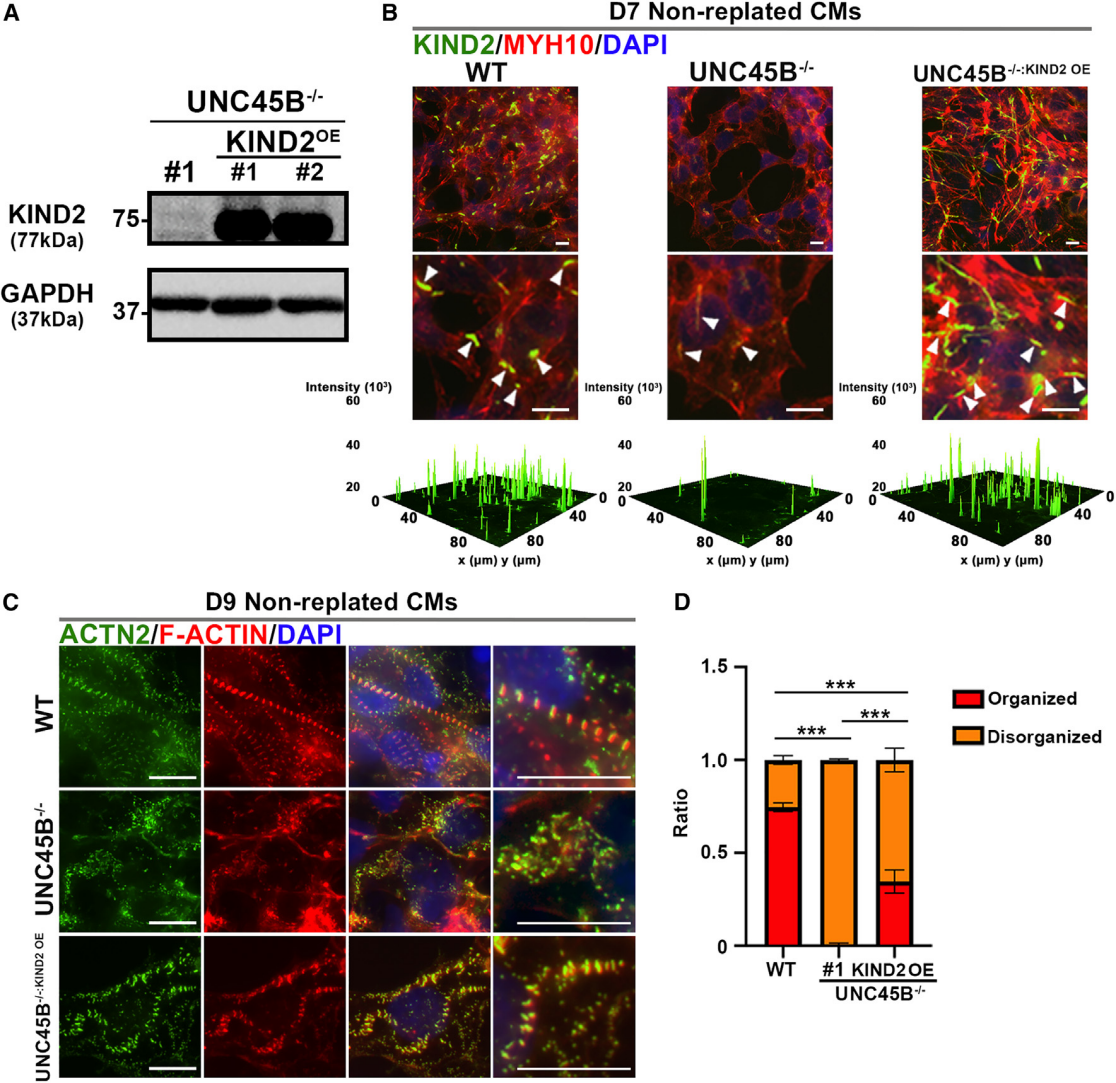
Ectopic expression of full-length KIND2 in a UNC45B^−/−^ line rescues the protocostamere phenotype (A) Western blots of day 7 UNC45B^−/−^-CMs and UNC45B^−/−:KIND2 OE^-CMs. (B) Representative IF images of non-replated WT, UNC45B^−/−^-CMs, and UNC45B^−/−:KIND2 OE^-CMs harvested at day 8 and stained for KIND2 and MYH10. Representative fluorescence intensity 3D surface plots of KIND2 signal in non-replated WT-CMs, UNC45B^−/−^-CMs, and UNC45B^−/−:KIND2 OE^-CMs. Note that KIND2 and MYH10 IF signals strongly overlap in both WT-CM and UNC45B^−/−:KIND2 OE^-CMs. (C) Representative IF images of non-replated WT, UNC45B^−/−^-CMs and UNC45B^−/−:KIND2 OE^-CMs harvested at day 9 and stained for ACTN2 and F-ACTIN. (D) Rescue efficiency was quantified on the basis of ACTN2 staining. Note that ∼34.7% ± 3.62% of ACTN2 accumulation was rescued in the UNC45B^−/−:KIND2 OE^-CMs. Results represent data from at least three independent experiments. Scale bars: 10 μm. See also Figure S6.

Apart from these known UNC45B binding partners, our co-IP/MS analysis also identified the protocostamere marker KIND2 (Table S3). Western blotting of our UNC45B pull-down samples with KIND2 and other protocostamere markers (i.e., ITGB1, vinculin [VCL] and paxillin [PXN]) revealed that only KIND2 interacts with UNC45B (Figure 5C), and not the other protocostamere markers (Figure S5A). Strikingly, IF staining revealed that expression of all these protocostamere markers (KIND2, ITGB1, VCL, and PXN) in nucleation sites was substantially reduced in UNC45B^−/−^-CMs relative to WT-CMs at day 7 (non-replated) and day 20 (replated) (Figures 5D–5H and S5B–S5F). As KIND2 strongly co-localizes with UNC45B at early differentiation day 5 (i.e., the premyofibril stage), we wanted to examine KIND2 expression in our *UNC45B*-knockout lines using western blot. We found that levels of KIND2 were significantly reduced in UNC45B^−/−^-CMs relative to WT-CMs (Figure 5I). Although co-localization of other protocostamere markers (VCL, PXL, and ITGB1) with sarcomere nucleation sites was significantly diminished, as revealed by immunostaining analysis, the protein levels of those protocostamere markers were not significantly different between WT-CMs and UNC45B^−/−^-CMs (Figure S5G). Thus, we propose that UNC45B is involved in protocostamere formation and that it modulates KIND2 expression levels. To further demonstrate that KIND2 expression levels modulate UNC45B-mediated protocostamere formation, we ectopically expressed KIND2 in a UNC45B^−/−^ line (hereafter denoted UNC45B^−/−:KIND2 OE^) (Figure 6A). Notably, IF staining of KIND2 and MYH10 revealed their strong co-localization in both WT and UNC45B^−/−:KIND2 OE^ CMs at day 7, indicating that the protocostamere-defective phenotype can be rescued by overexpressing KIND2 in the UNC45B^−/−^ line (Figure 6B). Next, we wanted to determine if ACTN2 accumulation and a failure of F-ACTIN polymerization in the UNC45B^−/−^ line are attributable to ACTN2 not being able to anchor to protocostameres. Importantly, IF staining of ACTN2 and F-ACTIN displayed filamentous-like structures in day 9 (not replated) and day 30 (replated) UNC45B^−/−:KIND2 OE^-CMs (Figures 6C, 6D, and S6), supporting that ACTN2 accumulation and failed F-ACTIN polymerization are associated with the protocostamere deficiency.

### Ectopic expression of UNC45B does not affect sarcomere structure

**Figure 7:**
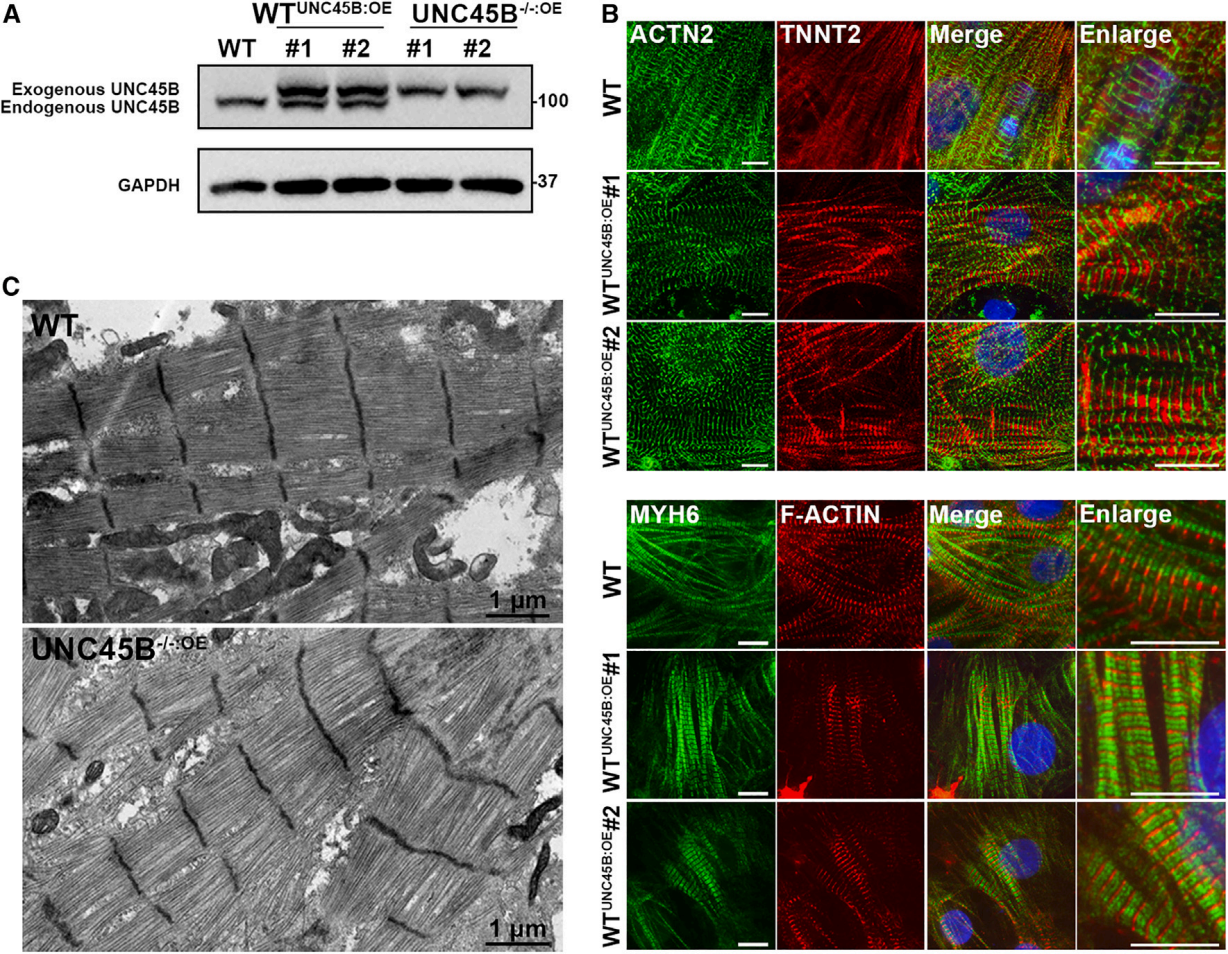
Effect of ectopic UNC45B expression in WT-CMs and UNC45B^−/−:^-CMs (A) Western blots of the protein expression levels of UNC45B in WT-CMs, WT^UNC45B-OE^-CMs, and UNC45B^−/−:OE^-CMs. Exogenous and endogenous UNC45B are indicated. (B) Representative TEM images of WT-CMs and UNC45B^−/−:OE^-CMs. Scale bars are indicated. (C) Representative IF images of replated WT-CMs and WT^UNC45B-OE^-CMs harvested at day 30 and stained for the ACTN2 and TNNT2 (upper panel) or for MYH6 and F-ACTIN (bottom panel). Scale bars: 10 μm. Results represent data from at least three independent experiments.

Previous studies have shown that UNC45B overexpression in zebrafish and *C. elegans* disrupts myosin thick filament and enhances myosin degradation (Bernick et al., 2010; Landsverk et al., 2007; Ni et al., 2011). In order to determine if ectopic expression of UNC45B affects human sarcomere assembly, we overexpressed UNC45B in WT (denoted WT^UNC45B^) and UNC45B^−/−^ (denoted UNC45B^−/−:OE^) hESC-CMs (Figure 7A). First, we performed IF staining for sarcomere markers (ACTN2, TNNT2, MYH6, and F-ACTIN) to examine the structure of sarcomeres in WT^UNC45B^-CMs. In contrast to previous observations for zebrafish and *C. elegans*, we detected well-organized sarcomeric structures in the WT^UNC45B^-CMs (Figure 7B), indicating that ectopic UNC45B in hESCs does not affect their sarcomere structures. However, ectopic UNC45B overexpression in a UNC45B^−/−^ mutant line did rescue its knockout phenotypes, as the UNC45B^−/−:OE^-CMs displayed beating contractility equivalent to WT (Video S2) and TEM analysis revealed well-organized sarcomeric structures (Figure 7C, lower panel).

Video S2. Beating contraction of UNC45B^-/-:OE^-CMs

Taken together, our findings demonstrate that (1) ablation of *UNC45B* impairs protocostamere formation (representing the initiating sites of myofibrillogenesis), so Z bodies (ACTN2, TTN, F-ACTIN) cannot bind to protocostameres to form mature Z lines and actin thin filament cannot be polymerized, and (2) *UNC45B* knockout perturbs myosin folding, so MYH6 cannot replace NMM MYH10, arresting myofibrillogenesis at an early stage and eliciting disrupted sarcomeres displaying filamentous-like structures.

## Discussion

In this study, we first used hESC-CMs as a model to elucidate the spatiotemporal expression of core myofibrillogenesis markers (ACTN2, TTN, MYH6, MYH10, and F-ACTIN), protocostameric markers (ITGB1 and KIND2) and a molecular chaperone (UNC45B). We propose a model that sarcomere assembly starts from directed cardiac differentiation at day 5 when (1) protocostameres form as initial sites of cardiac myofibrillogenesis; (2) NMM MYH10 is highly expressed and co-localizes with the protocostamere marker KIND2; (3) ACTN2, TTN-N, and F-ACTIN form Z bodies (representing Z line precursors), and expression of muscle myosin MYH6 and UNC45B begins; (4) then, on day 7, F-ACTIN polymerizes to form actin thin filaments, and muscle myosin MYH6 starts to replace NMM MYH10; and, ultimately, (5) on day 10, ACTN2, TTN, MYH6, F-ACTIN, and UNC45B display a filamentous morphology and form sarcomeres.

Intriguingly, UNC45B co-localized with both KIND2 and MYH6. We ablated *UNC45B* from hESCs and found that doing so impaired protocostamere formation, so that the Z line anchor protein ACTN2 could not bind to protocostameres, resulting in ACTN2 accumulation and F-ACTIN polymerization in UNC45B^−/−^-CMs. We have further demonstrated that UNC45B mediates protocostamere formation by regulating expression of KIND2.

The dynamic process of sarcomere assembly has been characterized previously using immunohistochemical analyses on non-human CMs (Rhee et al., 1994; Schultheiss et al., 1990). Moreover, because of the limited proliferative ability of human CMs, our knowledge of the process of cardiac myofibrillogenesis remains incomplete. Chopra et al. performed elegant experiments using human iPSC (hiPSC)-CMs as a model and showed that human cardiac sarcomere assembly starts at a site of cell-matrix adhesion (known as the protocostamere) (Chopra et al., 2018). In order to observe the process of sarcomerogenesis, in that study they replated day 30 hiPSC-CMs to trigger sarcomere re-assembly and demonstrated that β-cardiac myosin-titin-protocostameres form a mechanical connection required for sarcomere assembly (Chopra et al., 2018). We recently established a protocol for culturing hESCs on Matrigel-coated cover slides, enabling direct harvesting of hESC-CMs at different time points of directed cardiac differentiation (Lu et al., 2022). In this study, our non-replated hESC-CM model enabled us to recapitulate both the dynamic spatiotemporal expression pattern and interactions between sarcomeric proteins during the human sarcomere assembly process. Moreover, our approach has further addressed fundamental questions regarding sarcomere assembly. First, we show that sarcomeres start to assemble at day 5 of directed cardiac differentiation, representing a cardiac progenitor stage. Second, though previous studies have shown that TTN cannot be recognized until after thick filament assembly (Fulton and Alftine, 1997), we found that both TTN-N and TTN-C could be detected at the same stage of differentiation (day 5), albeit at low expression levels and with punctate signal patterns. Third, we reveal that UNC45B is also expressed in the protocostamere area. Taken together, we have uncovered that sarcomere assembly starts at the cardiac progenitor stage of cardiac differentiation and also revealed differences between humans and other species in terms of the spatiotemporal expression patterns of sarcomeric proteins during sarcomere assembly.

Recently, we showed that aberrant splicing of ACTN2 or TTN disrupts ACTN2-TTN interaction, resulting in MYH6 accumulation around the nucleus. Consequently, MYH6 cannot replace MYH10 (Lu et al., 2022). Indeed, we observed that TTN-N co-localizes with ACTN2 and forms Z bodies at day 5. Moreover, Chopra et al. also showed that truncated TTN in A bands and ACTN2 lacking its C terminus cannot bind to cardiac myosin and TTN, respectively, resulting in significantly impaired sarcomere assembly (Chopra et al., 2018). Taken together, these data suggest that the mechanical force generated by protocostamere-ACTN2-TTN-MYH6 assembly may account for MYH6-MYH10 replacement.

UNC45B can be dynamically translocated in the cytoplasm as sarcomere development progresses and it overlaps with various markers, shuttling between the cytoplasm, A bands (myosin thick filament), and Z lines (Etard et al., 2008). Accordingly, diffuse expression patterns and diverse functions of UNC45B during myosin assembly have been observed and postulated previously (Barral et al., 1998; Bernick et al., 2010; Chen et al., 2012; Landsverk et al., 2007). However, multiple lines of evidence suggest that UNC45B also performs critical functions in early cardiac myofibrillogenesis: (1) knockout of *UNC45* in *C. elegans* causes embryonic lethality at a stage corresponding to the onset of myofibrillogenesis (Venolia and Waterston, 1990); (2) UNC45 co-localizes with NMM and integrin adhesion sites at developing sarcomeric Z lines in *Drosophila*, implying that UNC45 and NMM are involved in the formation of cell-matrix attachment complexes (Lee et al., 2011); and (3) zebrafish *unc45b* mutants display delayed nucleation of α-actinin and mislocalization of NMM (Myhre et al., 2014). Our findings are consistent with UNC45B being involved in early cardiac myofibrillogenesis. Importantly, we demonstrate that UNC45B binds to the KIND2 and mediates its expression, facilitating proper protocostamere formation, and thus provide a mechanistic model for early cardiac myofibrillogenesis. Interestingly, knockdown/knockout of KIND2 in zebrafish and mouse ESCs results in a failure of myofibril attachment to the protocostamere, as well as reduces ITGB1 activity (Dowling et al., 2008; Zhang et al., 2019) corroborating the function of KIND2 in the protocostamere.

Moreover, in the zebrafish model, Myhre et al. showed that unc45b mediated costamere formation by regulating NMM expression (Myhre et al., 2014). However, in our study, we found that ablation of UNC45B enhanced MYH10 expression. Moreover, ablation of MYH10 from hiPSC-CMs did not affect sarcomere assembly (Chopra et al., 2018), further indicating that MYH10 is important for zebrafish sarcomere assembly, but not critical for that process in human.

Additionally, knockdown of zebrafish *Unc45b* causes Z line disorganization (Hawkins et al., 2008). Moreover, Unc45b deficiency in *Xenopus* results in delayed polymerization of Z disc formation (Geach and Zimmerman, 2010), implying that it is required for Z line organization. Here, we have also revealed an accumulation of Z lines in UNC45B^−/−^-CMs. However, ACTN2 is still able to bind to the TTN-N and F-ACTIN, enabling Z body formation. Importantly, ectopic expression of KIND2 in UNC45B-knockout line rescued protocostamere-deficient phenotypes, so ACTN2 can consequently form filamentous-like structures and F-ACTIN can be polymerized. Therefore, we have demonstrated that ACTN2 accumulation is due to a deficiency of protocostamere formation. Consequently, ACTN2 cannot anchor to protocostameres and establish the nucleation sites where ACTN2 and F-ACTIN assemble into the sarcomeric structure.

Additionally, even though UNC45B strongly co-localized with MYH10, ablation of UNC45B actually enhanced MYH10 expression. Although MYH10 knockout in mice elicits embryonic lethality (Tullio et al., 1997), ablation of MYH10 from hiPSC-CMs did not affect sarcomere assembly (Chopra et al., 2018), indicating that MYH10 may not be critical for human sarcomere assembly.

Moreover, even though UNC45B did not affect the transcript levels of sarcomere markers (e.g., *MYH6*, *MYH10*, *ACTN2*, *KIND2*), we found that 28 genes are down-regulated in day 7 UNC45B^−/−^-CMs. Interestingly, many of these genes perform CM-related functions: (1) *IRX4* has been shown involved in heart development by regulating expression of chamber-specific genes, and the loss of *Irx4* in mice results in cardiac decompensation (Kim et al., 2012). (2) PITX2 plays crucial roles in inner curvature remodeling and ventricular chamber expansion (Tessari et al., 2008). (3) *ALPK3* regulates heart development (Jorholt et al., 2020). Moreover, ALPK3 together with UNC45B and KIND2 and have been identified in the N-cadherin interactome (Li et al., 2019), suggesting potential interactions among these three proteins at the costamere. (4) *FREM2* mutants led to septa and valve malformations (Hardell et al., 1984). (5) *PCDHA10* is expressed predominantly in the atria of the heart and mutations in the mouse PCDHA10 ortholog (PCDHA9) resulted in bicuspid aortic valve (Zhu et al., 2022). (6) *LAMA4* is highly abundant in the heart, and mutations in this gene are associated with dilated cardiomyopathy (Wang et al., 2006).

Notably, RNA-seq data revealed an altered expression in a larger number of genes in d30 (1,847 up- and down-regulated genes) compared with that in day 7 (38 up- and down-regulated genes) in the UNC45B^−/−^-CMs. As a mechanosignaling hub linking the sarcomere and extracellular matrix, the protocostamere deficiency could lead to abnormal transcriptomic regulation (Henderson et al., 2017). It is possible that the DEGs observed in RNA-seq data are attributed to direct or indirect effects of UNC45B ablation. Thus, in addition to functioning as a molecular chaperone, UNC45B can also modulate expression of genes conducting CM-related functions and it would be interested to further investigate in the future.

In conclusion, we used hESCs to reveal the spatiotemporal process of cardiac myofibrillogenesis. Moreover, we have uncovered the function of UNC45B in human sarcomere assembly and demonstrated that UNC45B mediates KIND2 to form protocostameres, representing the initial sites of sarcomere assembly.

## Experimental procedures

### Resource availability

#### Corresponding author

Requests for further information or more detailed protocols should be directed to and will be fulfilled by the corresponding author, Su-Yi Tsai (suyitsai@ntu.edu.tw).

#### Materials availability

This study did not generate new unique reagents.

### Cell culture

Human H9 ESCs and directed cardiac differentiation were cultured and performed as previously described (Lu et al., 2022). For further information on generation of knockout hESC lines, overexpression of UNC45B and KIND2 in UNC45B-knockout or WT hESC lines, see supplemental experimental procedures.

### IF analysis, western blotting, fluorescence-activated cell sorting, flow cytometry, real-time qPCR, transmission electron microscopy, and RNA isolation, sequencing, and analyses

These methods were performed as previously described (Lu et al., 2022). For the detailed information, see supplemental experimental procedures.

### Co-IP and shotgun proteomic identifications by MS

WT-CMs and UNC45B-FLAG-CMs were cultured in two 10 cm dishes until they attained ∼90% confluency and then harvested using Accutase. Pellets were washed with 1x PBS and resuspended in ice-cold lysis buffer (50 mM Tris-HCl [pH 7.5], 1 mM EDTA, 150 mM NaCl, 1% Triton X-100, 10% glycerol, 1 mM PMSF) with protease inhibitor cocktail (Roche). For the detailed information, see supplemental experimental procedures.

### Statistics

Statistical analyses were conducted in Prism (version 7). To test data normality, a Shapiro-Wilk test was performed. For normally distributed data, a parametric two-tailed t tests (2-group data) or one-way ANOVA (>2-group data) followed by Tukey’s post hoc test was used to calculate statistical significance. For data that failed the normality test, a Mann-Whitney test (2-group data) or Kruskal-Wallis test (>2-group data) followed by Dunn’s post hoc test was performed. The notation for p values is as follows: ^∗^p < 0.05, ^∗∗^p < 0.01, and ^∗∗∗^p < 0.001.

## Author contributions

S.H.-A.L., Y.-H.W., L.-Y.S., Z.-T.H., T.-H.W., H.-Y.W., and C.Y., design, collection, and analysis of data; H.-Y.W., performance of IPA; S.-Y.T., conception and design of experiments, data collection and analyses, and manuscript writing.

## Data Availability

RNA-seq data are available from the Gene Expression Omnibus (GEO) repository database (GSE229229). MS data are available from the ProteomeXchange database (PXD042075). Raw data and images are available upon request to the corresponding author.

## Supplemental information

Document S1. Figures S1–S8 and Tables S1 and S2

Table S3. Protein lists from Co-IP followed by mass spectrometry

Document S2. Article plus supplemental information
