# Supplementary material for: Cardiac myofibrillogenesis is spatiotemporally modulated by the molecular chaperone UNC45B
Source: Stem Cell Reports. 2023 Jun 8;18(7):1405–20. doi: 10.1016/j.stemcr.2023.05.006 (PMC10362501; doi:10.1016/j.stemcr.2023.05.006)
Supplement: Document S1. Figures S1–S8 and Tables S1 and S2 [file mmc1.pdf]

Stem Cell Reports, Volume *18*

Supplemental Information

Cardiac myofibrillogenesis is spatiotemporally modulated by the molecular chaperone UNC45B

Serena Huei-An Lu, Yi-Hsuan Wu, Liang-Yu Su, Zi-Ting Hsu, Tzu-Han Weng, Hsin-Yu Wang, Chiao Yu, Paul Wei-Che Hsu, and Su-Yi Tsai

Supplemental Information

Cardiac Myofibrillogenesis is Spatiotemporally Modulated by the Molecular Chaperone UNC45B

Serena Huei-An Lu[#], Yi-Hsuan Wu[#], Liang-Yu Su[#], Zi-Ting Hsu[#], Tzu-Han Weng, Hsin-Yu Wang, Chiao Yu, Paul Wei-Che Hsu, Su-Yi Tsai[*]

Supplementary Experimental Procedures

hESC culture and directed cardiac differentiation

Human H9 ESCs were purchased from WiCell. All hESCs used in this study were derived from the MYH6:mCherry reporter line(Tsai et al., 2020). hESCs were grown on a Matrigel (Corning, 354277)-coated plate in Essential 8 medium (Gibco, A1516901) supplemented with E8 Supplement (Gibco, A1517101) and Normocin (InvivoGen, ant-nr-2) at 37 °C in 5% CO_2. The culture medium was exchanged daily. We used 0.5 mM EDTA (Invitrogen, 15575038) for routine passaging of hESCs. Accutase (BD Biosciences, 561527) was used for single-cell dissociation. Directed cardiac differentiation was conducted as described previously(Lu et al., 2022). In brief, hESCs were cultured in hESC medium until they attained ~80-90% confluency. At differentiation day 0, hESCs were treated with 12 μM CHIR99021 (CHIR, Tocris Bioscience) in RPMI (Gibco, 11875085) supplemented with B27 minus insulin (Gibco, A1895601) for 24 h. CHIR was removed the next day. At day 3, differentiated cells were treated with 5 μM WNT antagonist I (IWR-1, Tocris Bioscience) for 2 days. IWR-1 was removed at day 5. At day 7, B27 minus insulin in cardiac differentiation medium was changed to RPMI-B27 (Gibco, 17504044). Beating cells were typically observed around day 6-7. Both the lactate metabolic-selection method(Tohyama et al., 2013) and fluorescence-activated cell sorting (FACS) were used to purify hESC-CMs.

Generation of UNC45B-knockout hESC lines using the CRISPR/Cas9 technique

Two sets of sgRNA sequences were designed using the website http://chopchop.cbu.uib.no. The knockout strategy was as described previously[11]. In brief, the sgRNA sequences were ligated into pX330-U6-Chimeric_BB-CBh-hSpCas9 vector (Addgene, plasmid #42230)(Cong et al., 2013; Ran et al., 2013). MYH6:mCherry hESCs were transfected by electroporation using an Amaxa Human Stem Cell Nucleofector Kit 2 (Lonza, VPH-5022) according to the manufacturer’s guidelines. After electroporation, cells were re-plated on Matrigel-coated plates with 10 μM ROCK inhibitor (Tocris) at a low cell density (100 cells/well of a 6-well plate). After ~10 days, single clones were picked and separated into two wells of a 96-well plate. When the cells reached 80-90% confluency, we added lysis buffer (Sigma, Cat. #L3289) and neutralization buffer (Sigma, Cat. #N9784) to isolate genomic DNA for genotyping and DNA sequencing. All primers are listed in Table 1.

Overexpression of UNC45B and KIND2 in UNC45B-knockout or wild-type hESC lines

Human UNC45B or KIND2 coding regions were amplified by polymerase chain reaction (PCR) using hESC-CM cDNA as template and then cloned into the pCMV3flag8HOIL-1L vector (Addgene, #50016). The UNC45B-FLAG or KIND2-FLAG genes hosting FLAG tags were subcloned into lentivector pLKO AS3w.bsd. For virus production, 10 μg of target vector (UNC45B-FLAG), lentiviral envelope and the packaging plasmids pCMV-VSV-G (Addgene

#8454), psPAX2 (Addgene, #12260) and pMD2.G (Addgene, #12259) were all transfected into 293T cells by means of a standard $CaCl_2$ transfection method. After 72 h, lentiviral supernatants were collected and filtered through a 0.45 μm syringe filter. UNC45B-knockout and wild-type (WT) hESCs were infected with UNC45B-FLAG or KIND2-FLAG lentivirus using a spin infection method, as described previously[11]. After infection, the medium was replaced with E8 medium. Two days later, Blasticidin (6.67 μg/ml, Gibco, R21001) was used to select UNC45B-FLAG or KIND2-FLAG -positive clones.

Immunofluorescence analysis

Day 5 to 10 hESC-CMs were cultured on Matrigel-coated coverslides. To replate day15 to 30 hESC-CMs, the cells at day 10 to 25 were dissociated into single cells using TrypLE Express (Gibco 12604021) combined with Collagenase Type IV (Gibco, 17104019) for 10-15 min at 37 °C. The cells were centrifuged at 300 *g* for 4 min and replated on Matrigel-coated coverslides or on an Ibidi 96-well plate (Ibidi, IB-89626). Cells then were fixed with 4% PFA for 8 min at room temperature (RT). Blocking was performed in 5% horse serum (Gibco) and 0.3% Triton X-100 (93443-500ML, Sigma-Aldrich) in PBS for 1 h at RT, followed by primary antibody incubation overnight at 4 °C or for 2 h at RT. The following antibodies were used: mouse anti-α-Actinin (Sarcomeric) (A7811, Sigma-Aldrich); rabbit anti-α-Actinin (Sarcomeric) (710947, Invitrogen); mouse anti-MYH6 (MAB8979, R&D Systems); mouse or rabbit anti-TNNT2 (Invitrogen, MA5-12960; ab45932, Abcam); mouse or rabbit anti-MYH10 (GTX634160, GeneTex; GTX133378, GeneTex); rabbit anti-TTN-N (TTN-1, Myomedix); rabbit anti-TTN-C (TTN-9, Myomedix); rabbit anti-HSP70 (10995-1-AP, Proteintech); rabbit anti-HSP90AA1 (GTX109753, GeneTex); rabbit anti-HSP90AB1 (GTX101448, GeneTex); rabbit anti-ITGB1 (GTX128839, GeneTex); rat anti-ITGB1 9EG7 (553715, BD Biosciences); rabbit anti-KINDLIN2 (GTX118359, GeneTex); mouse anti-PAXILLIN (610051, BD Biosciences); goat or rabbit anti-UNC45B (GTX88214, GeneTex; PA5-53648, Invitrogen); mouse anti-VINCULIN (V9131, Sigma-Aldrich); and Fluor 647 PHALLOIDIN Conjugate (A22287, Invitrogen). Antibodies were detected with Alexa-488-, Alexa-555- and Alexa-647-conjugated donkey secondary antibodies against mouse, goat or rabbit (1:1000, Invitrogen). Nuclei were counterstained with DAPI. Images were obtained using a fluorescence microscope (Olympus IX83).

Western blotting

Cell pellets were lysed using RIPA buffer containing 1% PMSF. Protein concentration was measure by the BCA method using a Pierce BCA Protein Assay Kit (23225, Thermo Fisher Scientific). Samples were resolved on 9-13.3% sodium dodecyl sulfate polyacrylamide gels (SDS-PAGE), which were then transferred to PVDF membrane at 100v for 120 min. Membranes were blocked using 5% non-fat dry milk in 1xTBST at RT for 1 h and then incubated with primary antibody at 4 °C overnight. The following antibodies were used: goat or rabbit anti-

UNC45B (GTX88214, GeneTex; PA5-53648, Invitrogen); mouse or rabbit anti-α-ACTININ (Sarcomeric) (A7811, Sigma-Aldrich; 710947, Invitrogen); rabbit anti-DDDDK tag (GTX115043, GeneTex); rabbit anti-HSP90AA1 (GTX109753, GeneTex); rabbit anti-HSP90AB1 (GTX101448, GeneTex); rabbit anti-ITGB1 (GTX128839, GeneTex); rabbit anti-KINDLIN2 (13562, Cell Signaling); mouse MYH6 (MAB8979, R&D Systems); rabbit anti-MYH10 (GTX133378, GeneTex); mouse PAXILLIN (610051, BD Biosciences); mouse anti-VINCULIN (V9131, Sigma-Aldrich); rabbit anti-BETA-ACTIN (ab1801, abcam); rabbit anti-GAPDH (GTX100118, GeneTex); and mouse anti-ALPHA TUBULIN (GTX628802, GeneTex). After appropriate washing with 1xTBST, the membranes were incubated with HRP goat anti-mouse IgG (Jackson ImmunoResearch, 115-035-003) or HRP goat anti-rabbit IgG (GeneTex, GTX213110) at RT for 30 min. Chemiluminescent assay was performed using ECL substrates, and signals were detected with a FUSION Solo S chemiluminescence imaging system (Vilber).

FACS and flow cytometry

hESC-CMs were dissociated into single cells using TrypLE Express (Gibco 12604021) combined with Collagenase Type IV (Gibco, 17104019) for 10-15 min at 37 °C. Cells were centrifuged at 300 *g* for 4 min and resuspended in FACS buffer containing DMEM without phenol red (Life Technologies, 21063-029), 1 mM EDTA, 25 mM HEPES (Corning, 13116004), and 5% fetal bovine serum (FBS). MYH6:mCherry-positive cells were collected using a BD FACS Aria Cell Sorter (BD Bioscience). Flow cytometric analysis of cardiac differentiation efficiency using TNNT2-FITC (Santa Cruz, sc-20025) and MYH6:mCherry signals was performed on an Aurora (Cytek) system.

RNA isolation, sequencing and analyses

Total RNA was extracted using a RNeasy Mini Kit (Qiagen, 74106). RNA purity, concentration and integrity were inspected using a NanoPhotometer® spectrophotometer (IMPLEN, CA, USA), a Qubit® RNA Assay Kit in a Qubit® 2.0 Flurometer (Life Technologies, CA, USA), and an RNA Nano 6000 Assay Kit in a Bioanalyzer 2100 system (Agilent Technologies, CA, USA), respectively. The mRNA sequencing libraries were prepared using a NEBNext® UltraTM RNA Library Prep Kit for Illumina® (NEB, USA), and 150 bp paired-read sequencing was performed using an Illumina HiSeq instrument. Clean data (clean reads) were obtained by removing low-quality reads and reads containing adapters or poly-N from the raw data. Clean reads were mapped to the human genome assembly using HISAT2. Raw read count data was filtered using iDEP (integrated Differential Expression and Pathway analysis, http://bioinformatics.sdstate.edu/idep/) by removing genes with <0.5 counts per million (CPM) across all samples in each dataset. Differential gene expression analysis based on DESeq2 was performed using iDEP. The resulting P-values were adjusted using the Benjamini and

Hochberg's approach to control for the false discovery rate. Genes identified with an adjusted P-value of <0.05 and a fold-change >1.5 were assigned as being differentially expressed. Canonical pathways and functional analysis of the differentially expressed genes were evaluated using Ingenuity Pathway Analysis (IPA; Qiagen Inc.). Scatter plots, Venn diagrams and heatmaps of differentially expressed genes were plotted in R Statistical Software (v4.2.0; R Core Team 2022). Gene ontology (GO) analysis was performed using DAVID Bioinformatics Resources v6.8 (https://david.ncifcrf.gov/).

Co-immunoprecipitation

WT-CMs cells and CMs overexpressing UNC45B-FLAG were cultured in two 10-cm dishes and harvested using Accutase. Pellets were washed with 1x PBS and resuspended in ice-cold lysis buffer (50 mM Tris-HCl pH 7.5, 1 mM EDTA, 150 mM NaCl, 1% Triton X-100, 10% glycerol, 1 mM PMSF) with protease inhibitor cocktail (Roche). After lysis, cell lysates were centrifuged to remove cell debris. The supernatant was collected and protein concentration was measured using a BCA Protein Assay Kit. Part of the supernatant was transferred to a new tube and served as input for co-immunoprecipitation. For sample pre-cleaning, Mag-Beads (TOOLS, TOPG/A-2) were placed on a magnetic stand and equilibrated with Mag-Beads wash buffer (PBS buffer with 0.02% Tween 20). Cell lysates were mixed with the beads and mouse IgG, and then incubated for 2 h at 4 °C with end-over-end rotation. For sample binding, Anti-FLAG® M2 Magnetic Beads (Sigma, M8823) were used and first equilibrated with FLAG-bead wash buffer (50 mM Tris-HCl pH 7.5, 1 mM EDTA, 150 mM NaCl, 0.5% NP-40, 10% glycerol, 1 mM dithiothreitol, 1 mM PMSF). Next, the pre-cleaned supernatant was incubated with equilibrated FLAG beads for 3 h at 4 °C with end-over-end rotation. Beads were collected and washed with wash buffer. Then, 3x FLAG peptide (30 µg/ml, Sigma-Aldrich) was added, and the mixture was incubated in an orbital shaker at 4 °C for 50 min to elute the immunoprecipitate. SDS-PAGE was used for detection and to separate the FLAG-associated immunoprecipitate. The SDS-PAGE gels were stained with Easy Blue-Plus CBB Stain Reagent (EBL, PEB-021000) for 1 h at RT and washed three times with ddH_2O. Each gel lane was cut into three fragments for detection.

Shotgun proteomic identifications by mass spectrometry (MS)

NanoLC−nanoESi-MS/MS analysis was performed on a Thermo UltiMate 3000 RSLCnano system connected to a Thermo Orbitrap Fusion mass spectrometer (Thermo Fisher Scientific, Bremen, Germany) equipped with a nanospray interface (New Objective, Woburn, MA). Peptide mixtures were loaded onto a 75 µm ID, 25-cm long PepMap C18 column (Thermo Fisher Scientific) packed with 2 µm particles with a pore with of 100 Å, and they were separated over 1 h using a segmented gradient from 5% to 35% solvent B (0.1% formic acid in acetonitrile) at a flow rate of 300 nl/min. Solvent A was 0.1% formic acid in water. The mass spectrometer was

operated in the data-dependent mode. In brief, survey scans of peptide precursors from 350 to 1600 *m*/*z* were performed at 240K resolution with a $2×10^5$ ion count target. Tandem MS was performed using an isolation window of 1.6 Da with the quadrupole, HCD fragmentation with a normalized collision energy of 30, and rapid-scan MS analysis in the ion trap. The MS^2 ion count target was set to $1×10^4$ and the max injection time was 50 ms. Only those precursors with charge state 2–6 were sampled for MS^2. The instrument was run in top speed mode with 3 s cycles. The dynamic exclusion duration was set to 15 s, with a 10 ppm tolerance around the selected precursor and its isotopes. Monoisotopic precursor selection was turned on.

Proteome Discoverer label-free quantification

The MS and tandem MS raw data were processed in Proteome Discoverer (v.2.4.0; Thermo Scientific, Waltham, MA, USA) and searched against the Swiss-Prot protein sequence database with the Mascot server (v.2.7.0; Matrix Science, Boston, MA, USA). Taxonomy was set as *Homo sapiens*. The search criteria used were trypsin digestion, static modification as carbamidomethyl (C), variable modification as oxidation (M), and allowing up to 2 missed cleavages, a mass accuracy of 10 ppm for the parent ion, and 0.6 Da for the fragment ion mass tolerance. Label-free quantification was performed without normalization. Protein ratio was calculated in Proteome Discoverer using a pairwise ratio-based approach. To identify potential protein candidates interacting with UNC45B, the tandem MS data were analyzed by selecting proteins with a unique peptide number >25 and an abundance ratio >1.5.

Quantitative real-time PCR (qRT-PCR)

We used 2 μg of total RNA to synthesize cDNA (Thermo Fisher Scientific, 18080400). Real-time qPCR was performed in a CFX384 (Bio-Rad) machine with Maxima SYBR Green qPCR Master mix (K0252, ThermoFisher). Differences between samples and controls were calculated based on the $2^{-\Delta\Delta CT}$ method and normalized by GAPDH. Statistical significance was determined using a two-tailed Student's t-test ($p < 0.05$). The primers are listed in Table S1.

Transmission electron microscopy (TEM)

hESC-derived CMs were replated on Matrigel-coated Aclar embedding film for 5 days. Cells were fixed with 2.5% glutaraldehyde, 2% paraformaldehyde, 0.1% tannic acid in 0.1 M cacodylate buffer (pH7.2) for 30 min. Cells were then washed three times with 0.2 M sucrose and 0.1% calcium chloride in 0.1 M cacodylate buffer (pH7.2), and then post-fixed in 1% OsO_4 in 0.1 M cacodylate buffer (pH7.2) for 30 min. Cells were washed with ddH_2O three times and stained with 1% uranyl acetate for 30 min. The cells were then washed for a further three times with ddH_2O before being dehydrated via an ethanol gradient (30%, 50%, 70%, 90%, 100%; for 5 min at each concentration). Samples were infiltrated with 1:1, 2:1 (EPON: 100% EtOH) and pure EPON resin. All above-described procedures were performed at RT. Samples

were embedded in EPON at 60 °C for 48 hr. After slicing, images of cell ultrastructures were captured using a Tecnai G2 Spirit TWIN electron microscope (FEI) equipped with a Gatan CCD Camera (794.10.BP2 MultiScan) and acquisition software DigitalMicrograph (Gatan).

Fig. S1

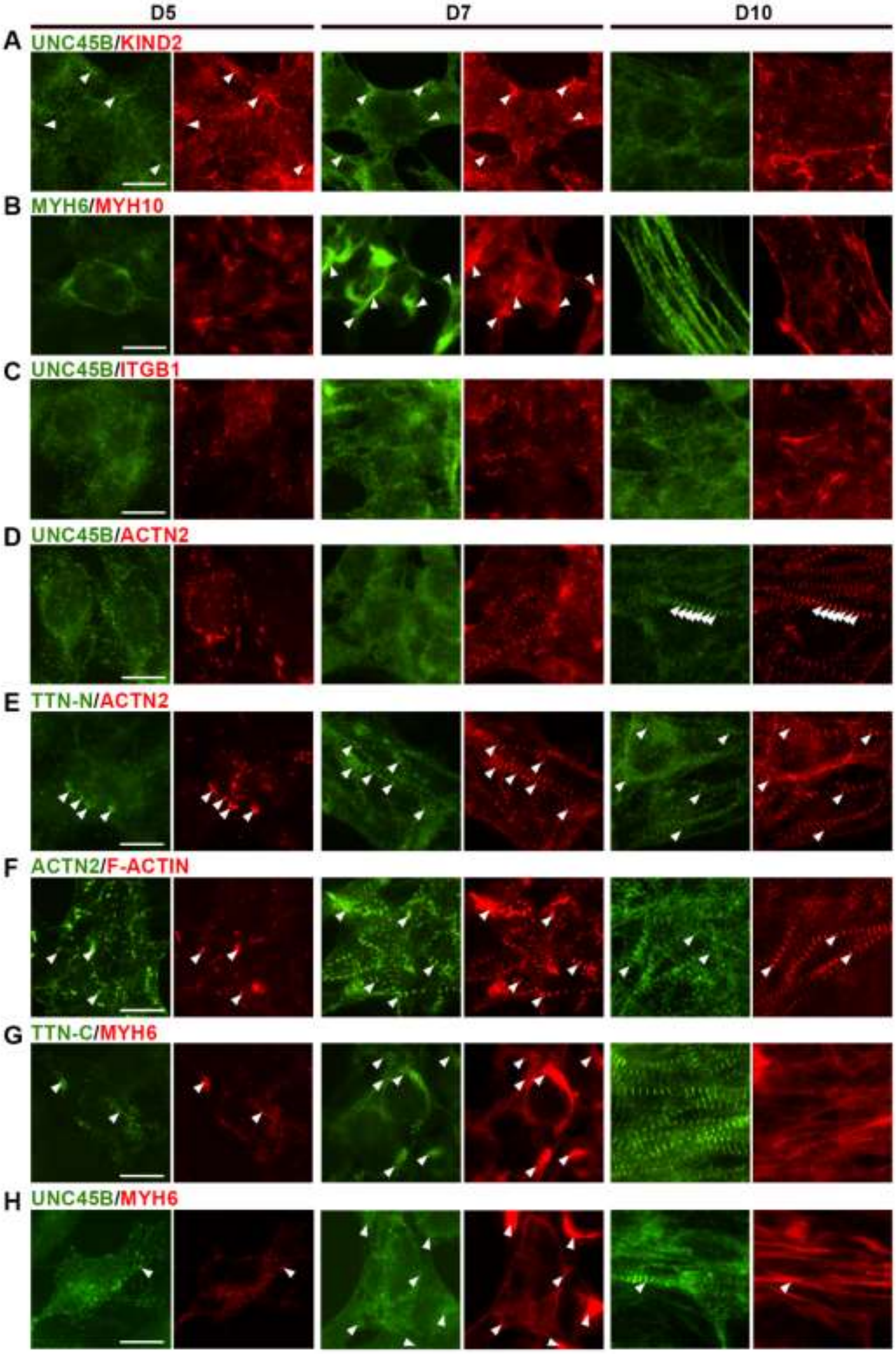

D5
D7
D10
A UNC45B/KIND2
B MYH6/MYH10
C UNC45B/ITGB1
D UNC45B/ACTN2
E TTN-N/ACTN2
F ACTN2/F-ACTIN
G TTN-C/MYH6
H UNC45B/MYH6

Supplemental Figure 1. Unmerged immunofluorescence images of cardiac myofibrillogenesis in human embryonic stem cell-derived cardiomyocytes (hESC-derived CMs). Related to Figure 1.

A-H. Representative unmerged immunofluorescence images of WT-CMs cultured on Matrigel-coated coverslides and harvested at the indicated time-points (D5, D7 and D10). Cells were stained for: (A) UNC45B (Unc-45 Myosin Chaperone B; green) and KIND2 (kindlin-2; red); (B) MYH6 (muscle myosin II; green) and MYH10 (non-muscle myosin IIB; red); (C) UNC45B (green) and ITGB1 (Integrin beta-1; red); (D) UNC45B (green) and ACTN2 (α-actinin; red); (E) TTN-N (N-terminus of titin; green) and ACTN2 (red); (F) ACTN2 (green) and F-ACTIN (red); (G) TTN-C (C-terminus of titin; green) and MYH6 (red); and (H) UNC45B (green) and MYH6 (red). Overlapping fluorescence signals in each panel are indicated by arrowheads. Scale bars: 10 μm.

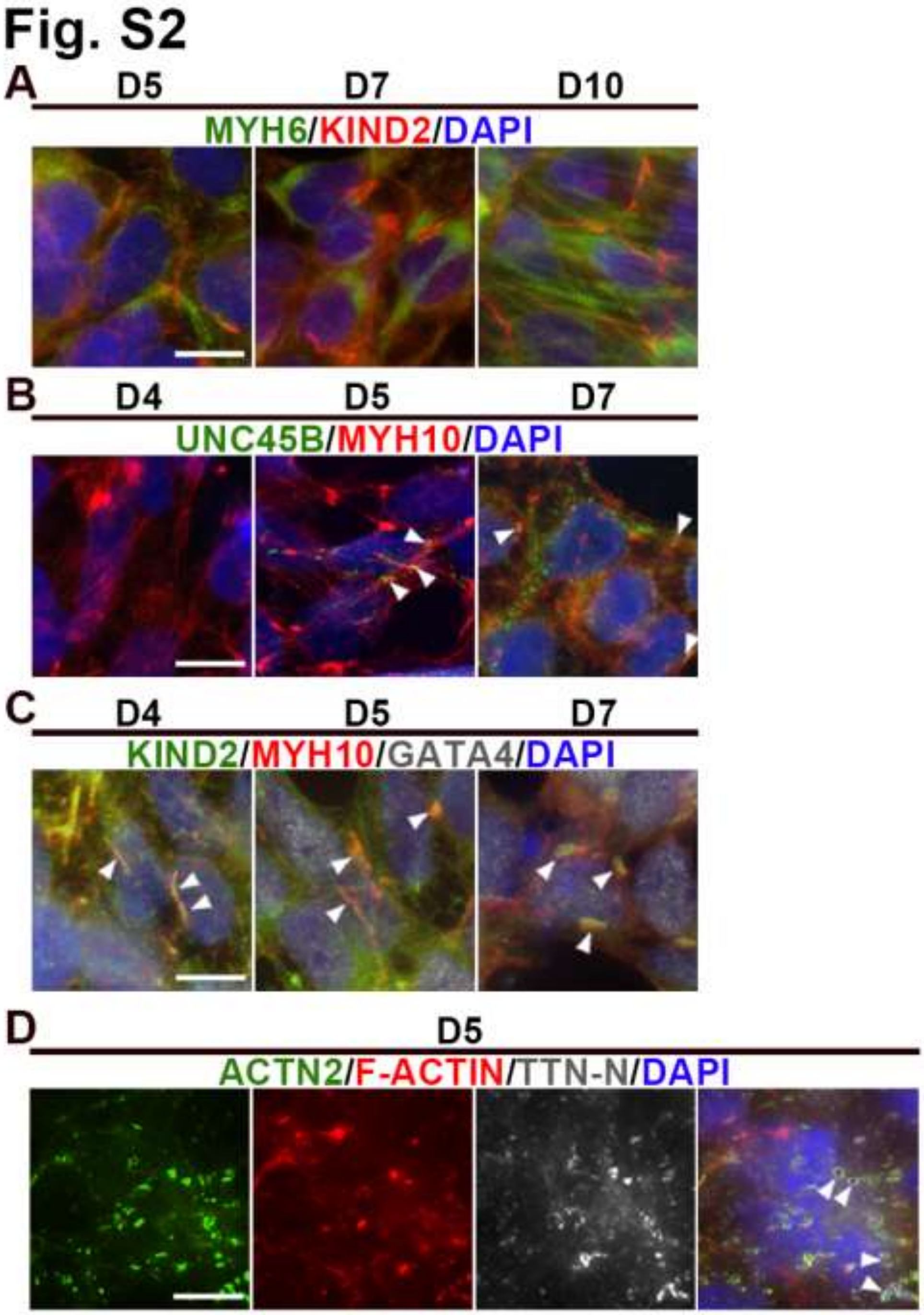

Supplemental Figure 2. Protocostameres are initiated on cardiac differentiation day 4. Related to Figure 1.

A.-**C**. Representative immunofluorescence images of WT-CMs harvested at the indicated time-points. Cells were stained with antibodies for: (A) MYH6 (green) and KIND2 (red); (B) UNC45B (green) and MYH10 (red); or (C) KIND2 (green), MYH10 (red), and GATA4 (white). **D**. Representative immunofluorescence images of WT-CMs harvested at day 5, stained for ACTN2 (green), F-ACTIN (red), and TTN-N (white). Overlapping fluorescence signals in each panel are indicated by arrowheads. Scale bars: 10 μm

Fig. S3

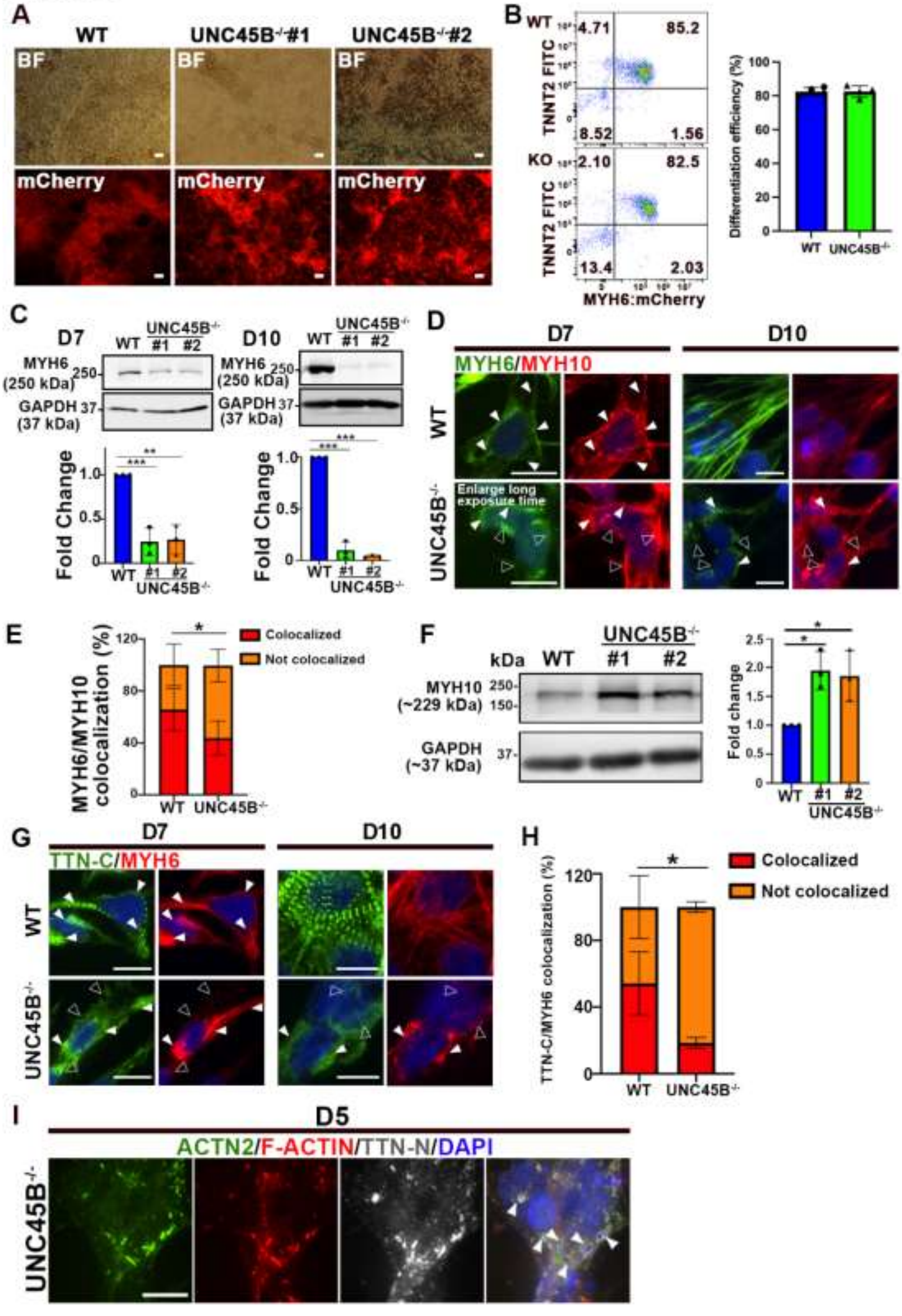
A
WT
UNC45B-/-#1
UNC45B-/-#2
BF
mCherry
B
WT
KO
TNNT2 FITC
MYH6:mCherry
4.71
85.2
8.52
1.56
2.10
82.5
13.4
2.03
Differentiation efficiency (%)
WT
UNC45B-/-
C
D7
D10
MYH6 (250 kDa)
GAPDH (37 kDa)
Fold Change
D
MYH6/MYH10
Enlarge long exposure time
E
MYH6/MYH10 colocalization (%)
Colocalized
Not colocalized
F
kDa
MYH10 (~229 kDa)
GAPDH (~37 kDa)
Fold change
G
TTN-C/MYH6
H
TTN-C/MYH6 colocalization (%)
I
D5
ACTN2/F-ACTIN/TTN-N/DAPI

Supplemental Figure 3. UNC45B$^{-/-}$ hESCs can differentiate into cardiomyocytes. Related to Figure 2 and Figure 3.

A. Images of WT and UNC45B$^{-/-}$ hESC-CM lines acquired under bright-field (upper panels; BF) and fluorescence (bottom panels; mCherry) microscopy at differentiation day 10. Scale bars: 100 µm. **B**. Representative flow cytometry plots of cardiomyocyte differentiation efficiency, as determined by flow cytometry of TNNT2-FITC-conjugated and MYH6:mCherry signal (WT n=4, UNC45B$^{-/-}$ line n=4). Quantification results of flow cytometry analysis (right panel). Error bars represent SD. **C.** Western blots and quantification data for MYH6 signal in day 7 and day 10 WT-CMs and UNC45B$^{-/-}$-CMs. GAPDH served as the loading control. Error bars represent SD. Statistical significance is indicated: *$p < 0.05$. **$p < 0.01$. ***$p < 0.005$. **D**. Representative unmerged immunofluorescence images of WT-CMs and UNC45B$^{-/-}$-CMs cultured on Matrigel-coated coverslides and harvested at the indicated time-points (D7 and D10). Cells were stained for MYH6 (green) and MYH10 (red). Overlapping fluorescence signals in each panel are indicated by white arrowheads. Non-overlapping fluorescence signals in each panel are indicated by empty arrowheads. **E.** Quantification of overlapping fluorescence signals of MYH6 and MYH10. Error bars represent SD. Statistical significance is indicated: *$p < 0.05$. **F.** Western blots and quantification data for MYH10 signal in WT-CMs and UNC45B$^{-/-}$-CMs. GAPDH served as the loading control. Error bars represent SD. Statistical significance is indicated: *$p < 0.05$. **G.** Representative unmerged immunofluorescence images of non-replated WT-CMs and UNC45B$^{-/-}$-CMs and harvested at the indicated time-points (D7 and D10). Cells were stained for TTN-C (green) and MYH6 (red). Overlapping fluorescence signals in each panel are indicated by white arrowheads. Non-overlapping fluorescence signals in each panel are indicated by empty arrowheads. **H.** Quantification of overlapping fluorescence signals of MYH6 and MYH10. Error bars represent SD. Statistical significance is indicated: *$p < 0.05$. **I.** Representative immunofluorescence images of UNC45B$^{-/-}$-CMs harvested at day 5 and stained for ACTN2 (green), F-ACTIN (red), and TTN-N (white). Overlapping fluorescence signals in each panel are indicated by arrowheads. Merged fluorescence images with DAPI staining (blue) are also shown. Scale bars: 10 µm.

Fig. S4

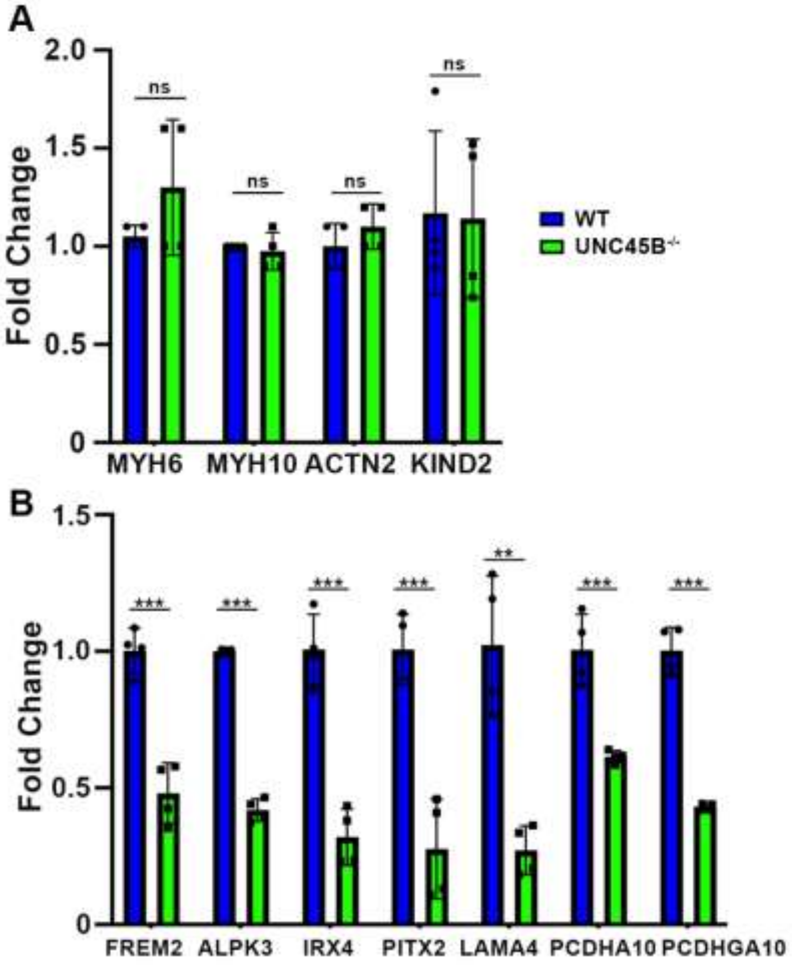

Supplemental Figure 4. The transcript levels of sarcomere markers and cardiomyocyte-related genes in WT-CMs and UNC45B$^{-/-}$-CMs. Related to Figure 4.

A-B. The expression levels of sarcomeric genes (A) and cardiomyocyte-related genes (B) in day 7 WT-CMs and UNC45B$^{-/-}$-CMs.

Fig. S5

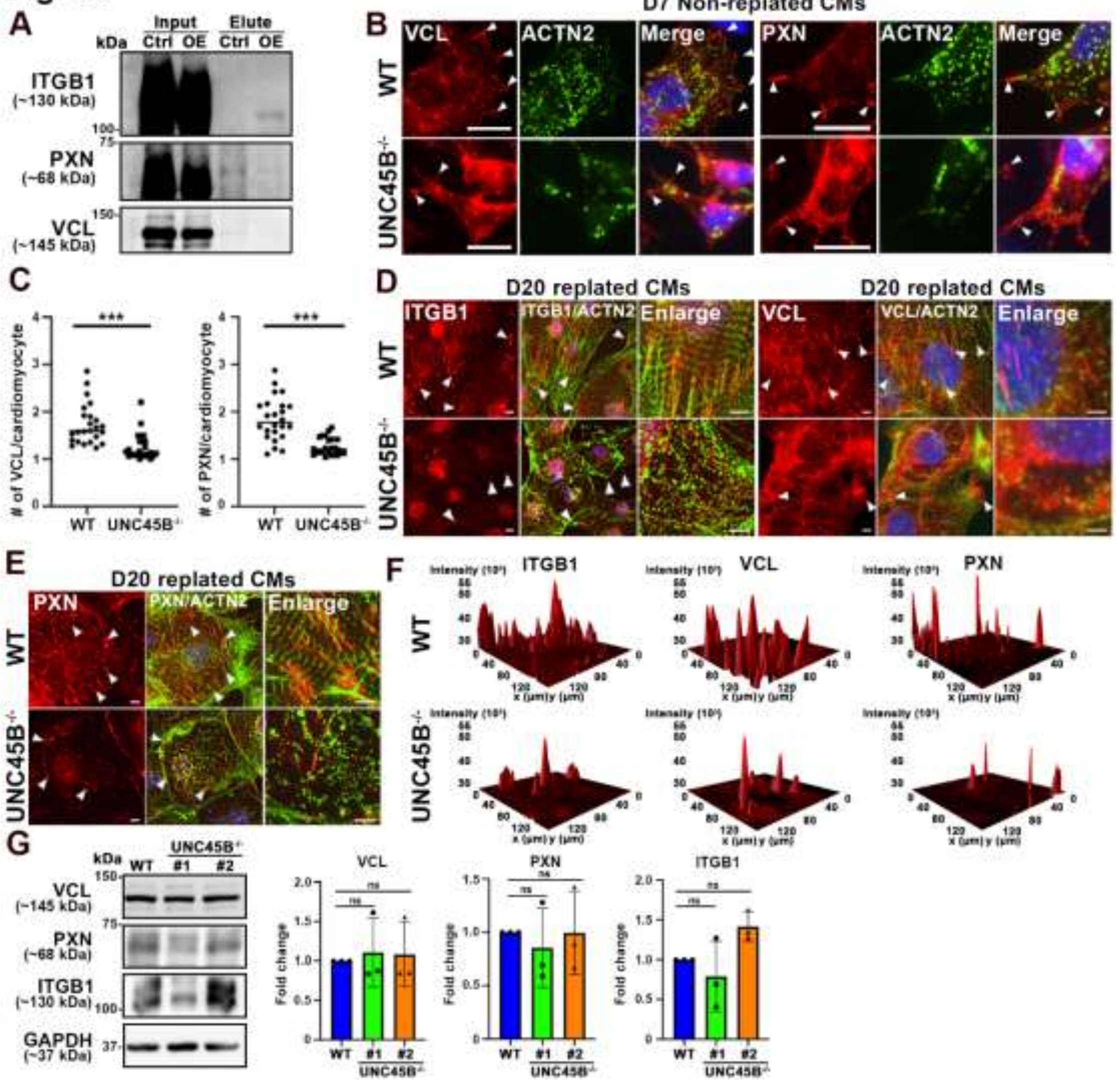

Supplemental Figure 5. UNC45B deletion significantly reduces numbers of protocostamere nucleation sites. Related to Figure 5.

A. Association of UNC45B-FLAG with ITGB1, PXN, and VCL, as observed by co-IP assay followed by Western blot analysis. **B**. Representative immunofluorescence images of WT-CMs and UNC45B$^{-/-}$-CMs harvested at day 7. Cells were stained for antibodies against (b, left panel) VCL (red) and ACTN2 (green) or (right panel) PXN (red) and ACTN2 (green). **C**. Quantification results for numbers of VCL (left) and PXN (right) nucleation sites per cardiomyocyte in WT-CMs and UNC45B$^{-/-}$-CMs (VCL n=24, PXN n=24). Error bars represent SD. Statistical significance is indicated: ***$p < 0.001$. **D**.-**E**. Representative immunofluorescence images of replated WT-CMs and UNC45B$^{-/-}$-CMs harvested at day 20. Cells were stained for antibodies against (D, left panel) ITGB1 (red) and ACTN2 (green), (D, right panel) VCL (red) and ACTN2 (green), or (E) PXN (red) and ACTN2 (green). Scale bars: 10 μm. **F**. Representative fluorescence intensity 3D surface plots of ITGB1 (left), VCL (middle), and PXN (right) signals for replated WT-CMs (top)

and UNC45B$^{-/-}$-CMs (bottom) harvested at day 20, illustrating protocostamere sites. **G**. Western blots and quantification data for VCL, PXN, and ITGB1 in WT-CMs and UNC45B$^{-/-}$-CMs. GAPDH served as the loading control. Error bars represent SD.

Fig. S6

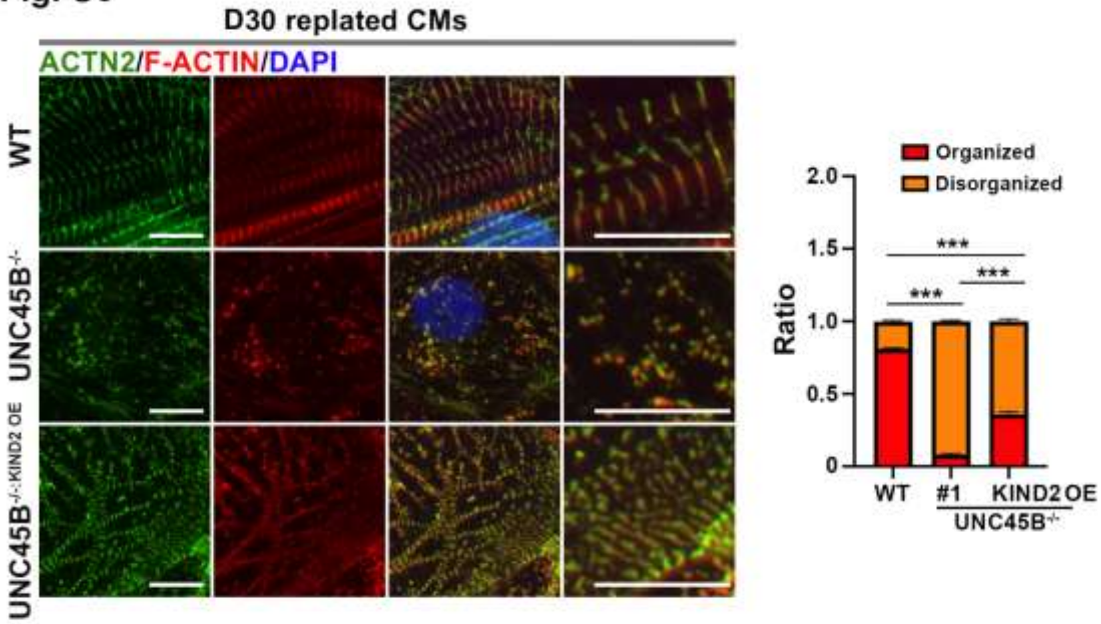

Supplemental Figure 6. ACTN2 accumulation and a failure of F-ACTIN polymerized can be improved by ectopic expression of full-length KIND2 in a UNC45B$^{-/-}$ line. Related to Figure 5.

Left panel. Representative immunofluorescence images of re-plated day 30 WT, UNC45B$^{-/-}$-CMs and UNC45B$^{-/-:KIND2\ OE}$-CMs and stained for the Z-line marker ACTN2 (green) and Actin thin marker F-ACTIN (red). **Right panel.** Rescue efficiency was quantified based on ACTN2 staining. Note that ~35.9±0.98 % of ACTN2 accumulation was rescued in the UNC45B$^{-/-:KIND2\ OE}$-CMs. Results represent data from at least three independent experiments. Scale bars: 10 μm.

Table S1. Primer lists. **Related to Figure 2, Figure 6 and Figure 7.**

Name of Primer	Sequence (5’ to 3’)
UNC45B sgRNA#1_F	GGCCACGCTTTATCGGAACC
UNC45B sgRNA#1_R	GGTTCCGATAAAGCGTGGCC
UNC45B sgRNA#2_F	CGCCACAAATAGCTACAGCC
UNC45B sgRNA#2_R	GGCTGTAGCTATTTGTGGCG
UNC45B genotype_F	GCTGACCAGACAGAGCTTCTCCTG
UNC45B genotype_R	GAATGAAGCCCTCTGGATCCCGAG
qPCR UNC45B_F	TGACCTCTGCAATTTGCTTG
qPCR UNC45B_R	ATATGGGGAGGGGAAATGAG
qPCR GAPDH_F	GAAGGTGAAGGTCGGAGTC
qPCR GAPDH_R	GAAGATGGTGATGGGATTT
UNC45B OE_1_NheI_F	GCTAGCATGGCAGAGGTGGAAGCGGTAC
UNC45B OE_1_SalI_R	GTCGACCCTTCCGCAAACTGCCTGAGAC
UNC45B OE_2_SalI_F	GTCGACAGAAAAACTGGCCAAACAGTGTCGC
UNC45B OE_2_NotI_R	GCGGCCGCAGACACTGGTTTAATGAAACCATAATCCATG
KIND2 OE_NheI_F	GCTAGCATGGCTCTGGACGGGATAAGGATG
KIND2 OE_NotI_R	GCGGCCGCCACCCAACCACTGGTAAGTTTGTAG
FLAG_EcoRI_R	GAATTCCTATTTATCGTCATCATCTTTGTAGTCCTTG
UNC45B OE_sequence_F	GCCAGAGCCACAGTGATTCTGC
UNC45B OE_sequence_R	CCTTGAGCCACAATGGTGCCTC
pLAS3W sequence	GTTCGGCTTCTGGCGTGTG
pX330 sequence	GGACTATCATATGCTTACCG

Table S2. Ingenuity Canonical Pathway analysis of day 7 RNA sequencing data. Related to Figure 4.

Ingenuity Canonical Pathways	Log (p-value)	z-score
GP6 Signaling Pathway	5.43	-2.111
Human Embryonic Stem Cell Pluripotency	4.23	-1.732
CREB Signaling in Neurons	3.4	-3.13
Intrinsic Prothrombin Activation Pathway	3.39	-2.236
LXR/RXR Activation	3.25	-2.646
Neurovascular Coupling Signaling Pathway	3.07	-2.714
cAMP-mediated signaling	3.01	-1.508
HIF1α Signaling	2.89	-2.53
G-Protein Coupled Receptor Signaling	2.61	-1.528
Breast Cancer Regulation by Stathmin1	2.04	-2.668
S100 Family Signaling Pathway	1.86	-2.236
STAT3 Pathway	1.78	-2
Role of PKR in Interferon Induction and Antiviral Response	1.76	-1.633
Glutamate Receptor Signaling	1.75	-2
Corticotropin Releasing Hormone Signaling	1.55	-1.633
eNOS Signaling	1.5	-2.236
BAG2 Signaling Pathway	1.42	2
Immunogenic Cell Death Signaling Pathway	1.32	2
NOD1/2 Signaling Pathway	1.17	2.449
Synaptic Long Term Depression	1.1	-2.449
IL-15 Production	0.936	-2
Calcium Signaling	0.932	-2.236
White Adipose Tissue Browning Pathway	0.807	-2
Endocannabinoid Neuronal Synapse Pathway	0.726	-2
Semaphorin Neuronal Repulsive Signaling Pathway	0.719	-2
Synaptogenesis Signaling Pathway	0.699	-1.89
FAK Signaling	0.642	-2.524
Phagosome Formation	0.446	-2.309
CLEAR Signaling Pathway	0	2
Chaperone Mediated Autophagy Signaling Pathway	0	2.236
Mitochondrial Dysfunction	0	-2
Phospholipase C Signaling	0	-2
Systemic Lupus Erythematosus In B Cell Signaling Pathway	0	-1.633

Supplemental References

Cong, L., Ran, F.A., Cox, D., Lin, S., Barretto, R., Habib, N., Hsu, P.D., Wu, X., Jiang, W., Marraffini, L.A., and Zhang, F. (2013). Multiplex genome engineering using CRISPR/Cas systems. Science *339*, 819-823.

Lu, S.H., Lee, K.Z., Hsu, P.W., Su, L.Y., Yeh, Y.C., Pan, C.Y., and Tsai, S.Y. (2022). Alternative Splicing Mediated by RNA-Binding Protein RBM24 Facilitates Cardiac Myofibrillogenesis in a Differentiation Stage-Specific Manner. Circ Res *130*, 112-129. 10.1161/circresaha.121.320080.

Ran, F.A., Hsu, P.D., Wright, J., Agarwala, V., Scott, D.A., and Zhang, F. (2013). Genome engineering using the CRISPR-Cas9 system. Nat Protoc *8*, 2281-2308.

Tohyama, S., Hattori, F., Sano, M., Hishiki, T., Nagahata, Y., Matsuura, T., Hashimoto, H., Suzuki, T., Yamashita, H., Satoh, Y., et al. (2013). Distinct Metabolic Flow Enables Large-Scale Purification of Mouse and Human Pluripotent Stem Cell-Derived Cardiomyocytes. Cell Stem Cell *12*, 127-137. https://doi.org/10.1016/j.stem.2012.09.013.

Tsai, S.Y., Ghazizadeh, Z., Wang, H.J., Amin, S., Ortega, F.A., Badieyan, Z.S., Hsu, Z.T., Gordillo, M., Kumar, R., Christini, D.J., et al. (2020). A human embryonic stem cell reporter line for monitoring chemical-induced cardiotoxicity. Cardiovasc Res *116*, 658-670. 10.1093/cvr/cvz148.
