# Supplementary material for: Cardiac myofibrillogenesis is spatiotemporally modulated by the molecular chaperone UNC45B
Source: Stem Cell Reports. 2023 Jun 8;18(7):1405–20. doi: 10.1016/j.stemcr.2023.05.006 (PMC10362501; doi:10.1016/j.stemcr.2023.05.006)
Supplement: Document S2. Article plus supplemental information [file mmc5.pdf]

Stem Cell Reports
Article
OPEN ACCESS

Cardiac myofibrillogenesis is spatiotemporally modulated by the molecular chaperone UNC45B

Serena Huei-An Lu,[1,5] Yi-Hsuan Wu,[1,5] Liang-Yu Su,[1,5] Zi-Ting Hsu,[1,5] Tzu-Han Weng,[1] Hsin-Yu Wang,[1] Chiao Yu,[1] Paul Wei-Che Hsu,[4] and Su-Yi Tsai[1,2,3,*]
[1]Department of Life Science, National Taiwan University, Taipei 10617, Taiwan
[2]Research Center for Developmental Biology and Regenerative Medicine, National Taiwan University, Taipei 10617, Taiwan
[3]Genome and Systems Biology Degree Program, National Taiwan University, Taipei 10617, Taiwan
[4]Institute of Molecular and Genomic Medicine, National Health Research Institutes, Miaoli County 350, Taiwan
[5]These authors contributed equally
*Correspondence: suyitsai@ntu.edu.tw
https://doi.org/10.1016/j.stemcr.2023.05.006

SUMMARY

Sarcomeres are fundamental to cardiac muscle contraction. Their impairment can elicit cardiomyopathies, leading causes of death worldwide. However, the molecular mechanism underlying sarcomere assembly remains obscure. We used human embryonic stem cell (hESC)-derived cardiomyocytes (CMs) to reveal stepwise spatiotemporal regulation of core cardiac myofibrillogenesis-associated proteins. We found that the molecular chaperone UNC45B is highly co-expressed with KINDLIN2 (KIND2), a marker of protocostameres, and later its distribution overlaps with that of muscle myosin MYH6. UNC45B-knockout CMs display essentially no contractility. Our phenotypic analyses further reveal that (1) binding of Z line anchor protein ACTN2 to protocostameres is perturbed because of impaired protocostamere formation, resulting in ACTN2 accumulation; (2) F-ACTIN polymerization is suppressed; and (3) MYH6 becomes degraded, so it cannot replace non-muscle myosin MYH10. Our mechanistic study demonstrates that UNC45B mediates protocostamere formation by regulating KIND2 expression. Thus, we show that UNC45B modulates cardiac myofibrillogenesis by interacting spatiotemporally with various proteins.

INTRODUCTION

Assembled by hundreds of proteins, the sarcomere is the basic contractile unit of heart muscle (Clark et al., 2002). Each sarcomere is defined by two Z lines consisting of an A band (myosin thick filament) and an I band (actin thin filament). Mutations in genes encoding sarcomere-associated proteins result in cardiomyopathies, leading causes of morbidity and mortality worldwide. Although the function and organization of the sarcomere have been studied extensively, the precise process of sarcomere assembly remains elusive.

Among current models of sarcomere assembly, the premyofibril model is the widely accepted framework (Rhee et al., 1994). In this model, integrin adhesion sites, which form protocostameres (initial sites of sarcomerogenesis), recruit the Z line anchor protein ACTN2, actin, and non-muscle myosin (NMM) MYH10 for premyofibril assembly. During the transition from nascent myofibrils to mature myofibrils, muscle myosin II (MYH6) starts to displace NMM II B (MYH10), and Z bodies (composed of ACTN2, ACTIN, and TITIN [TTN]) amalgamate other Z bodies laterally into a Z line to form the mature sarcomere (Rhee et al., 1994; Sanger et al., 2010; Sparrow and Schock, 2009). Apart from the scaffold proteins that assemble into the building blocks of sarcomeres, the sarcomere assembly process is modulated by multiple regulatory factors, such as RNA-binding proteins (RBPs) and molecular chaperones (Poon et al., 2012; Srikakulam et al., 2008).

Our previous study demonstrated that the RBP RBM24 mediates alternative splicing of core myofibrillogenesis genes in a stage-specific manner and that ACTN2 interacts with the N terminus of TTN (TTN-N), allowing MYH6 to bind the C terminus of TTN (TTN-C) (Lu et al., 2022). Here, we examine how a molecular chaperone regulates sarcomere assembly. Uncoordinated mutant number 45 (Unc45) is a key molecular chaperone exerting a critical role in myosin assembly during sarcomerogenesis (Barral et al., 2002; Lee et al., 2011). Unc45 was first discovered in *Caenorhabditis elegans* (Epstein and Thomson, 1974; Venolia et al., 1999), and two isoforms (UNC45A and UNC45B) have been identified in mammals (Price et al., 2002); whereas UNC45A is expressed ubiquitously in all mammalian cells, UNC45B is expressed primarily in skeletal and cardiac striated muscle cells (Price et al., 2002). Myosin forms a complex with multiple chaperones when it assembles, including Unc45b and heat shock protein 90 (Hsp90) (Etard et al., 2007). Ultimately, myosin is incorporated into thick filaments, but Unc45b and Hsp90 both migrate to and remain at the Z line (Etard et al., 2008). Notably, under stress conditions, Unc45b and Hsp90 move to A bands, preventing myosin from denaturing (Etard et al., 2008). Additionally, ablation of

This is an open access article under the CC BY license (http://creativecommons.org/licenses/by/4.0/).

Unc45b in mice elicited defective heart looping, and the mutant mice displayed embryonic lethality (Chen et al., 2012). *Unc45b* knockdown in zebrafish resulted in delayed nucleation of α-actinin and mislocalization of NMM (Myhre et al., 2014). Thus, UNC45B may exert functions in addition to modulating myosin folding during sarcomere assembly.

Human pluripotent stem cells (PSCs), including embryonic stem cells (ESCs) and induced PSCs (iPSCs), have the unique competence to differentiate into all cell types, including cardiomyocytes (CMs). Hence, human ESC (hESC)-derived CMs (hESC-CMs) provide great potential for studying sarcomere formation and cardiac development. Sarcomere assembly is a multi-step process, so we first used hESC-CMs to dissect the critical steps of the sarcomere assembly process using core myofibrillogenesis markers (ACTN2, MYH6, TTN, MYH10, and F-ACTIN), protocostamere markers (ITGB1 and KINDLIN2 [KIND2]), and a molecular chaperone (UNC45B). We established the following stepwise process of sarcomere assembly in our hESC-CM system: (1) protocostameres are initiated at directed cardiac differentiation day 4; (2) sarcomeres start to assemble and expression of muscle myosin MYH6 initiates at day 5, with premyofibrils being assembled by protocostameres, NMM, and Z bodies (ACTN2, TTN, and F-ACTIN); (3) at day 7 (the nascent myofibril stage), muscle myosin MYH6 replaces NMM MYH10 and completely colocalizes with TTN-C, with F-ACTIN also beginning to polymerize into actin thin filaments; and (4) at day 10, Z lines are established, and MYH6 fully replaces MYH10, thus completing the sarcomere assembly process.

Significantly, during the sarcomere assembly process, we found that UNC45B co-localizes with KIND2, MYH10, and MYH6 at different stages of sarcomere assembly. We sought to determine the function of *UNC45B*, so we ablated *UNC45B* from MYH6:mCherry cardiac reporter hESCs using the CRISPR-Cas9 technique. Interestingly, although hESCs with homozygous ablation of UNC45B (UNC45B$^{-/-}$) still differentiated into CMs, essentially no beating contractility was detectable in the mCherry-positive cells. Phenotypic analysis further revealed ACTN2 accumulation because of impaired protocostamere formation, failure to polymerize F-ACTIN, and MYH6 replacement defects in the mutant cell lines. Notably, our mechanistic study demonstrates that UNC45B facilitates expression of the protocostamere marker KIND2, with disruption of *UNC45B* resulting in impairment of the initiating site of sarcomere assembly. Thus, UNC45B directs human cardiac myofibrillogenesis by spatiotemporally modulating protocostamere formation and muscle myosin folding. In conclusion, we have used directed cardiac differentiation to establish the spatiotemporal expression pattern of cardiac myofibrillogensis genes and the function of a molecular chaperon during the process of human sarcomere assembly.

RESULTS

Deciphering the stepwise processes of cardiac myofibrillogenesis in hESC-derived CMs

To elucidate the stepwise process of human cardiac myofibrillogenesis, we cultured MYH6:mCherry cardiac reporter hESCs on Matrigel-coated cover slides and harvested hESC-CMs at differentiation days 5, 7, and 10 without replating, allowing us to observe the spatiotemporal process of sarcomere assembly. Immunofluorescence (IF) staining was conducted to examine the expression patterns of various markers for protocostameres (ITGB1 and KIND2), core myofibrillogenesis proteins (ACTN2, TTN, MYH6, MYH10, and F-ACTIN), and a molecule chaperone (UNC45B). Among these, MYH10, KIND2, ITGB1, and F-ACTIN can also be detected in non-cardiac cells. Expression of all cardiac-specific markers (UNC45B, ACTN2, TTN, and MYH6) we examined began on directed cardiac differentiation day 5 (Figures 1A–1H, S1A–S1H [unmerged images], and S2A–S2B), whereas NMM (MYH10) and the protocostamere marker KIND2 were co-localized on day 4 (Figure S2C), indicating that protocostameres form before other sarcomere components. Moreover, on day 5, we observed higher expression of UNC45B compared with other cardiac-specific markers (Figures 1D, 1H, S1D, and S1H). We also observed that on day 5, ACTN2 co-localized with TTN-N (stained with TTN-N-specific antibody) and actin thin filament (F-ACTIN), forming nucleation sites termed Z bodies (precursors of Z lines) (Figures 1E, 1F, and S2D). Furthermore, muscle myosin MYH6 co-localized with TTN-C (stained with TTN-C-specific antibody) and UNC45B (Figures 1G, 1H, S1G, and S1H). On day 7, MYH6 partially replaced NMM MYH10, so some MYH6-positive cells displayed signal overlap with MYH10 (Figure 1B, middle, and Figure S1B), and F-ACTIN began to polymerize into actin thin filaments (Figure 1F, middle, and Figure S1F). On day 10, MYH6 had fully replaced MYH10, with the former displaying a filamentous-like structure and the MYH10 solely being expressed at the cell periphery (Figure 1B, right panel, and Figure S1B). IF staining patterns for ACTN2, MYH6, and TTN were punctate-like (Figures 1D–1H and S1D–S1H), whereas those of MYH6, ACTN2, TTN, F-ACTIN, and UNC45B were filamentous-like (Figures 1B, 1D, 1E–1H, S1B, S1D, and S1E–S1H).

Intriguingly, IF staining revealed co-localization of UNC45B with the protocostamere marker KIND2 (Figures 1A and S1A), muscle myosin MYH6 (Figures 1H and S1H), as well as NMM MYH10 (Figure S2B). We also co-stained for the protocostamere marker KIND2 and

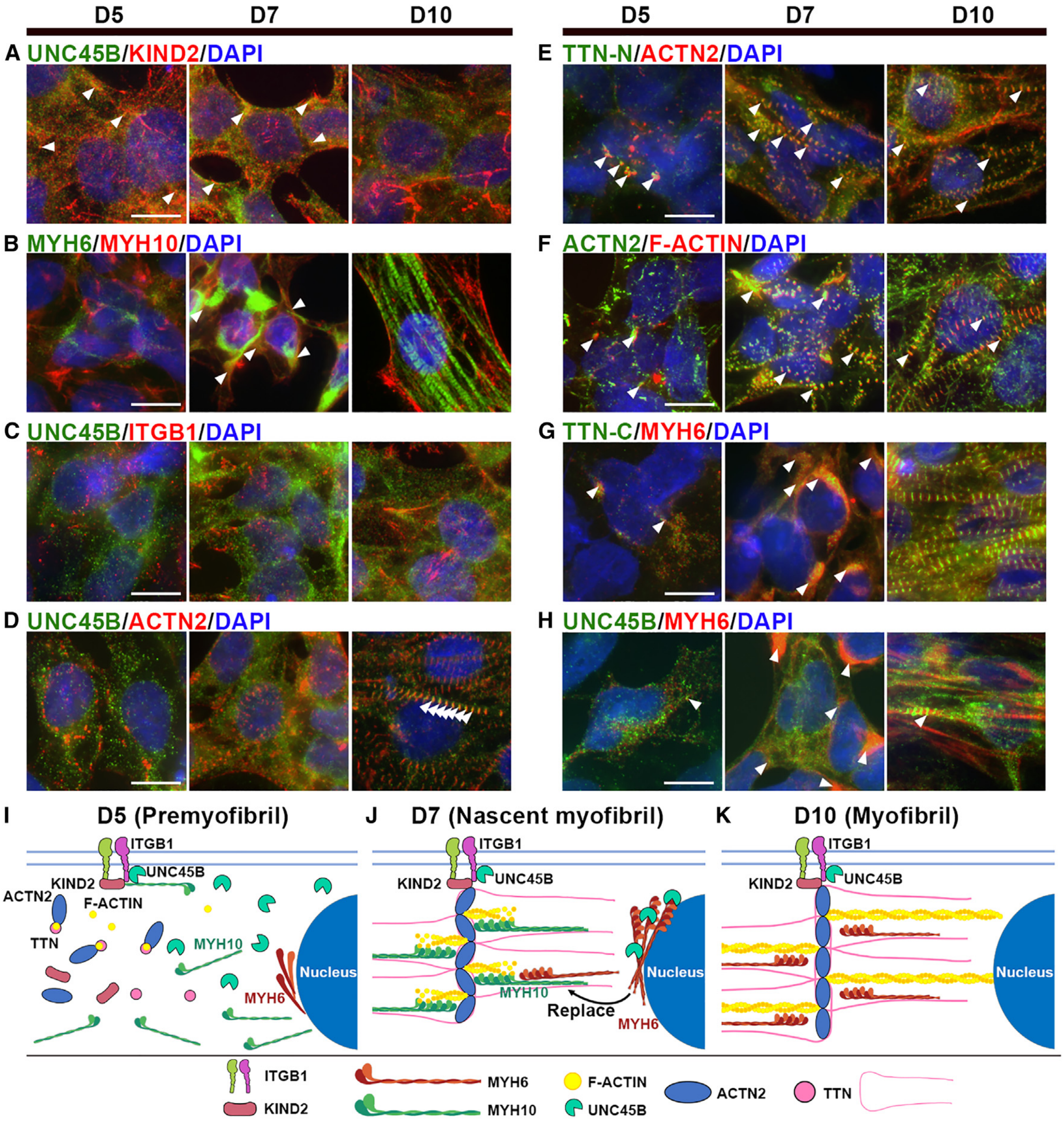

Figure 1. The stepwise process of cardiac myofibrillogenesis in human embryonic stem cell-derived cardiomyocytes

(A–H) Representative immunofluorescence (IF) images of WT-CMs cultured on Matrigel-coated cover slides and harvested at the indicated time points. Cells were stained for (A) UNC45B and KIND2, (B) MYH6 and MYH10, (C) UNC45B and ITGB1, (D) UNC45B and ACTN2, (E) TTN-N and ACTN2, (F) ACTN2 and F-ACTIN, (G) TTN-C and MYH6, and (H) UNC45B and MYH6. Overlapping fluorescence signals in each panel are indicated by arrowheads. All fluorescence images have been merged with DAPI staining. Scale bars: 10 μm.

(I–K) Schematics of sarcomere assembly and integrity at (I) day 5 (the premyofibril stage), (J) day 7 (nascent myofibril), and (K) day 10 (mature myofibril). Results represent data from at least three independent experiments.

See also Figures S1 and S2.

both muscle myosin MYH6 and NMM MYH10. Notably KIND2 only co-localized with MYH10, and not with MYH6 (Figures S2A and S2C). Thus, UNC45B co-localizes with various sarcomeric components (protocostamere, muscle myosin, NMM). Taken together, our IF analysis reveals that (1) by directed cardiac differentiation day 4, NMM MYH10 has bound to protocostameres (labeled by KIND2); (2) sarcomere assembly begins at day 5, with UNC45B being present in the protocostamere and NMM forming earlier than other tested sarcomeric components (Figure 1I); (3) core myofibrillogenesis proteins (ACTN2, TTN, MYH6, and F-ACTIN) displayed a punctate pattern

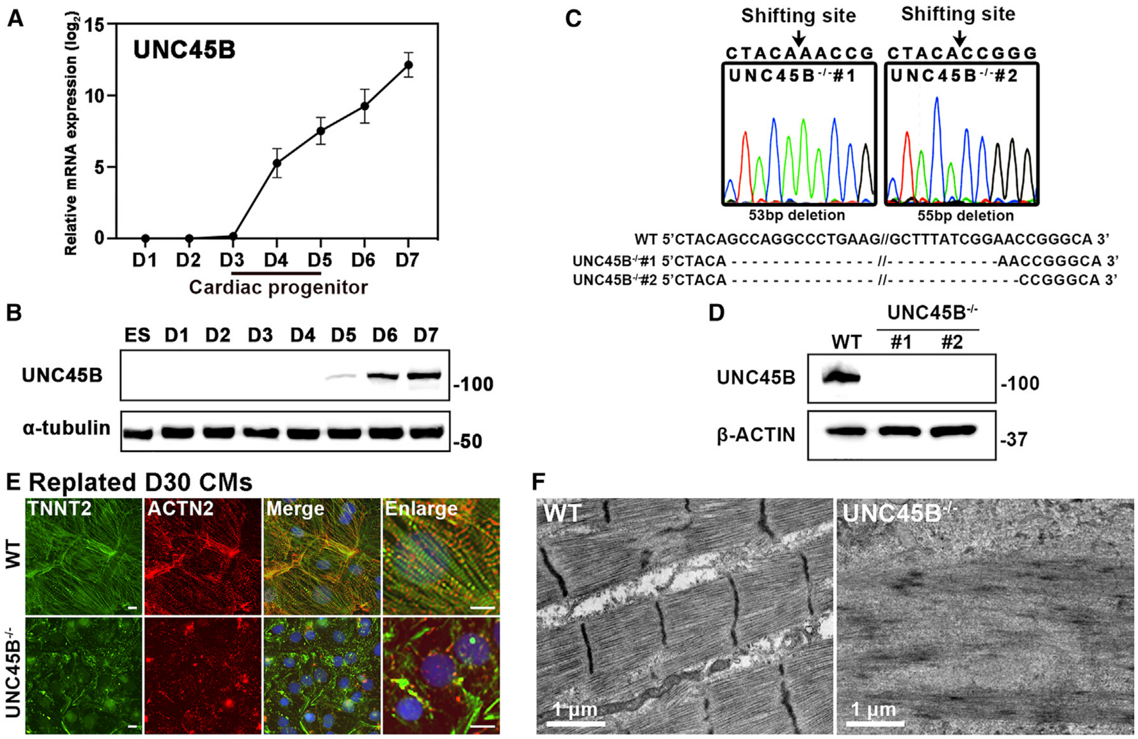

Figure 2. Ablation of *UNC45B* from hESCs

(A and B) Transcription (A) and protein (B) levels of *UNC45B* in WT hESCs during directed cardiac differentiation from day 1 to day 7, as determined by qRT-PCR (n = 3) and western blot. Error bars represent SD.

(C) DNA sequencing data of genomic DNA samples from the two homozygous *UNC45B*-knockout lines. Black arrows indicate the sgRNA cleavage sites.

(D) Protein levels of UNC45B in WT-CMs and UNC45B$^{-/-}$-CMs, as determined using western blot.

(E) Representative IF images of replated WT-CMs and UNC45B$^{-/-}$-CMs harvested at day 30 and stained for TNNT2 and ACTN2. Scale bars: 10 μm.

(F) Representative TEM images of WT-CMs and UNC45B$^{-/-}$-CMs. Scale bars are indicated.

See also Figure S3.

and low expression levels (Figure 1I); (4) ACTN2, together with TTN-N and actin thin filament (F-ACTIN), forms nucleation sites (i.e., Z bodies) (Figure S2D), with only a small proportion of muscle myosin MYH6 being bound to TTN-C and UNC45B (Figure 1I); (5) MYH6 starts to replace MYH10 by day 7, and F-ACTIN begins to polymerize into actin thin filaments (Figure 1J); and (6) by day 10, Z lines are established, and MYH6 has completely replaced MYH10, thereby completing the process of sarcomere assembly (Figure 1K).

UNC45B knockout does not affect cardiac differentiation efficiency, but the resulting cells lack contractility

Previous studies have shown that UNC45B plays a critical role in myosin assembly (Barral et al., 1998, 2002; Landsverk et al., 2007). However, we found that *UNC45B* expression began at the cardiac progenitor stage (UNC45B mRNA and protein were detected at days 4 and 5, respectively), and gradually increased at the CM stage (Figures 2A and 2B). Importantly, our IF analysis revealed that UNC45B is associated with different sarcomeric components (Figures 1A, 1D, 1H, S1A, S1D, S1H, and S2B). Thus, in order to determine if UNC45B conducts diverse functions in different components of the sarcomere, we ablated *UNC45B* from our previously published hESC cardiac reporter line (MYH6:mCherry) (Tsai et al., 2020) using a CRISPR-Cas9 approach. DNA sequencing further validated the deletion mutants. We selected two homozygous mutant lines harboring 53 or 55 bp deletions, respectively, for further study (Figure 2C). Western blot analysis revealed that UNC45B protein was essentially undetectable in both of these knockout lines (Figure 2D).

Next, we sought to assess if homozygous knockout of *UNC45B* (denoted UNC45B$^{-/-}$) affected CM differentiation. We observed that UNC45B$^{-/-}$ hESCs still

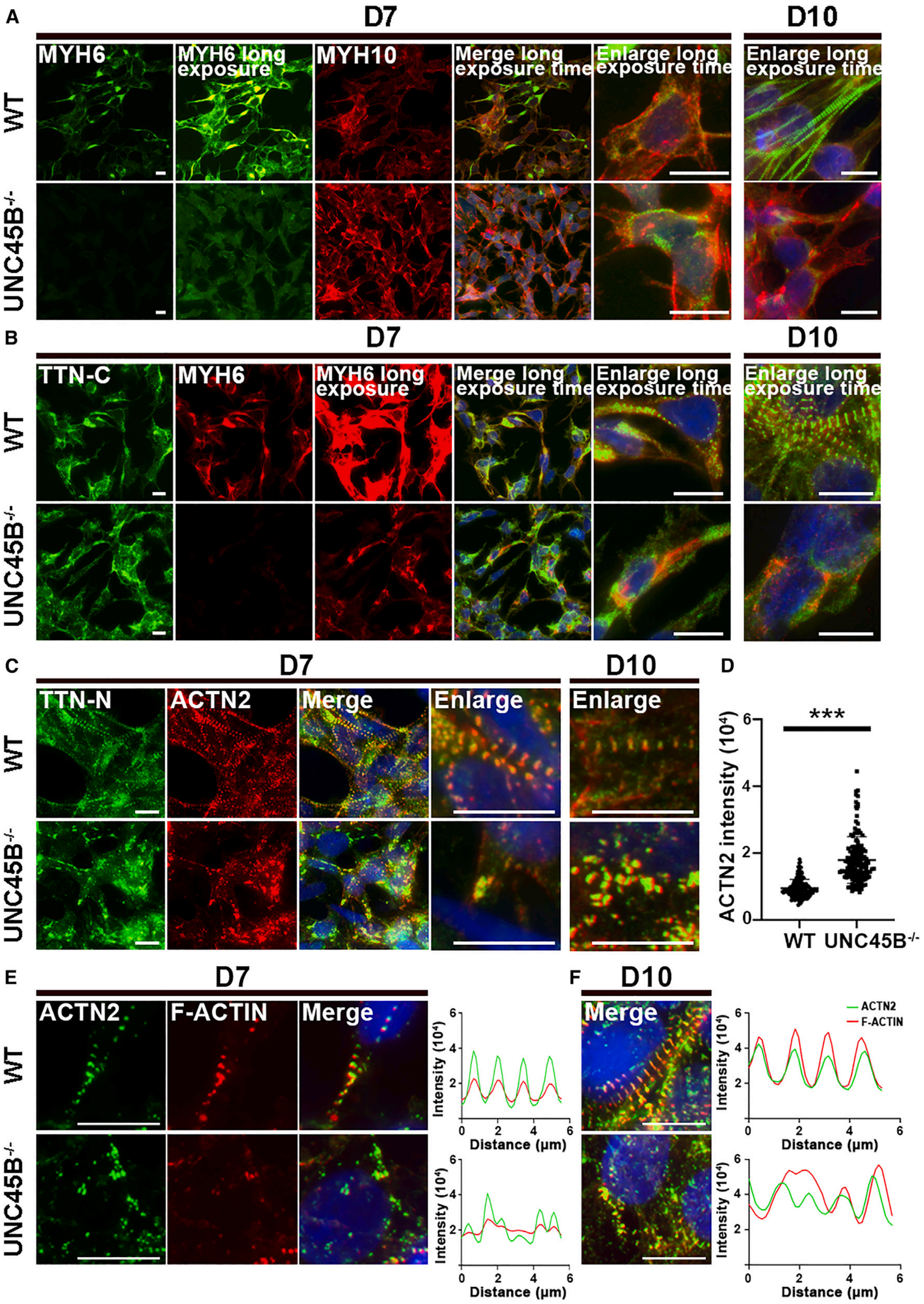

(legend on next page)

differentiated into CMs and displayed MYH6:mCherry signals (Figure S3A). Flow cytometry analysis using TNNT2 staining combined with MYH6:mCherry signals further demonstrated a similar differentiation efficiency compared with a wild-type (WT) line (Figure S3B). However, whereas WT-CMs beat regularly, the UNC45B$^{-/-}$-CMs did not exhibit any contractility (Video S1).

UNC45B$^{-/-}$-CMs exhibit severely disrupted sarcomeric structures

As UNC45B$^{-/-}$-CMs could not contract, we investigated if the phenotype is caused by impairment of sarcomere structures. To do so, we conducted IF staining for ACTN2 (Z line marker) and TNNT2 (actin thin filament) on hESC-CMs at differentiation day 30. Notably, we observed disordered Z lines and actin thin filaments in our UNC45B$^{-/-}$-CMs (Figure 2E). To further verify our IF results, we performed transmission electron microscopy (TEM) to visualize the sarcomeric structures of the hESC-CMs. Strikingly, in contrast to the well-organized sarcomere structures found in WT-CMs, UNC45B$^{-/-}$-CMs displayed aberrant filamentous structures (Figure 2F), supporting that UNC45B modulates sarcomere organization and that blocking its activity inhibits cell contractility.

To further investigate how sarcomere assembly is affected by *UNC45B* ablation at early differentiation days 7 and 10 (representing the nascent myofibril and mature myofibril stages, respectively), we conducted IF staining for core myofibrillogenesis markers (MYH6, MYH10, ACTN2, TTN, and F-ACTIN). UNC45B is involved in regulating folding of the myosin head domain (Srikakulam et al., 2008). Our IF staining using anti-MYH6 antibody revealed that levels of MYH6 were substantially reduced in UNC45B$^{-/-}$-CMs relative to WT (Figures 3A and 3B). Subsequently, we conducted western blot analysis to examine MYH6 protein expression. MYH6 levels were indeed significantly reduced in the UNC45B$^{-/-}$-CMs at differentiation days 7 and 10 (Figure S3C). We examined if the unfolded MYH6 affects MYH10 replacement and TTN binding during sarcomere formation, potentially leading to the reduced MYH6 levels observed in the mutant cell lines. In WT-CMs, MYH6 co-localized with MYH10 at early cardiac differentiation day 7 (Figures 3A and S3D), with MYH6 displaying filamentous structures at day 10 when MYH10 signal substantially diminished (Figure 3A, upper panel). However, in the UNC45B$^{-/-}$-CMs, most MYH6 did not co-localize with MYH10 at day 7 (Figures S3D and S3E), nor did it replace MYH10 (Figure S3D). Instead, in the mutant cells, MYH6 appeared to surround the nucleus at day 10 and did not form well-organized filaments (Figure 3A, lower panel, and Figure S3D). Western blotting analysis showed that amounts of MYH10 in fact increased in UNC45B$^{-/-}$-CMs at day 15 (Figure S3F), indicating that *UNC45B* knockout causes myosin (MYH6) protein degradation, thereby limiting replacement of MYH10 by MYH6. Moreover, TTN-C and MYH6 co-localized and surrounded the nucleus of WT-CMs at day 7 (Figure 3B, upper panel, and Figures S3G and S3H), forming filamentous structures in those cells by day 10 (Figure 3B, upper panel, and Figure S3G). In contrast, MYH6 did not co-localize with TTN-C on days 7 or 10 in UNC45B$^{-/-}$-CMs (Figure 3B, lower panel, and Figures S3G-H).

TTN-N and F-ACTIN can both bind to ACTN2, forming nucleation sites at day 5 (Figure S2D). As Z lines were severely disrupted in the UNC45B$^{-/-}$-CMs, we examined if this phenotype is due to impaired nucleation site formation. Accordingly, we conducted IF staining to determine if ACTN2 co-localizes with TTN-N and F-ACTIN. Even though Z lines were perturbed in UNC45B$^{-/-}$-CMs, ACTN2 still co-localized with TTN-N and F-ACTIN in both WT and UNC45B$^{-/-}$-CMs (Figures 3C–3F and S3I). Notably, we observed an accumulation of ACTN2 (Figures 3C–3F), as well as a lack of filamentous-like actin thin filaments (Figures 3E and 3F, lower panel), in UNC45B$^{-/-}$-CMs at days 7 and 10, indicating that the nucleation sites may not function properly. Thus, in addition to its role in myosin assembly, UNC45B most likely regulates Z line and actin filament formation.

In addition, as UNC45B is a molecule chaperon, it is most likely that UNC45B regulates these sarcomere markers (MYH6, MYH10, ACTN2, and KIND2) only in the protein level, but not their transcription. We therefore performed

Figure 3. ACTN2 accumulation and defective F-ACTIN polymerization during the nascent myofibril stage in UNC45B$^{-/-}$-CMs

(A and B) Representative IF images of MYH6, MYH10, and TTN-C in non-replated WT-CMs and UNC45B$^{-/-}$-CMs harvested at day 7 or day 10. Cells were stained for (A) MYH6 and MYH10 and (B) TTN-C and MYH6. To improve visualization of MYH6 signal in the UNC45B$^{-/-}$ cells, additional sets of MYH6 images with a longer exposure time are shown. Scale bars: 10 μm.

(C) Representative IF images of non-replated WT-CMs and UNC45B$^{-/-}$-CMs harvested at day 7 or day 10 and stained for TTN-N and ACTN2. Scale bars: 10 μm.

(D) Quantification results for ACTN2 signal intensity in WT-CMs and UNC45B$^{-/-}$-CMs at day 7, showing ACTN2 accumulation (n = 150). Error bars represent SD. Statistical significance is indicated: ***$p < 0.001$.

(E and F) Left panel: representative IF images of WT-CMs and UNC45B$^{-/-}$-CMs harvested at (E) day 7 and (F) day 10 and stained for ACTN2 and F-ACTIN. Scale bars: 10 μm. Results represent data from at least three independent experiments.

See also Figure S3.

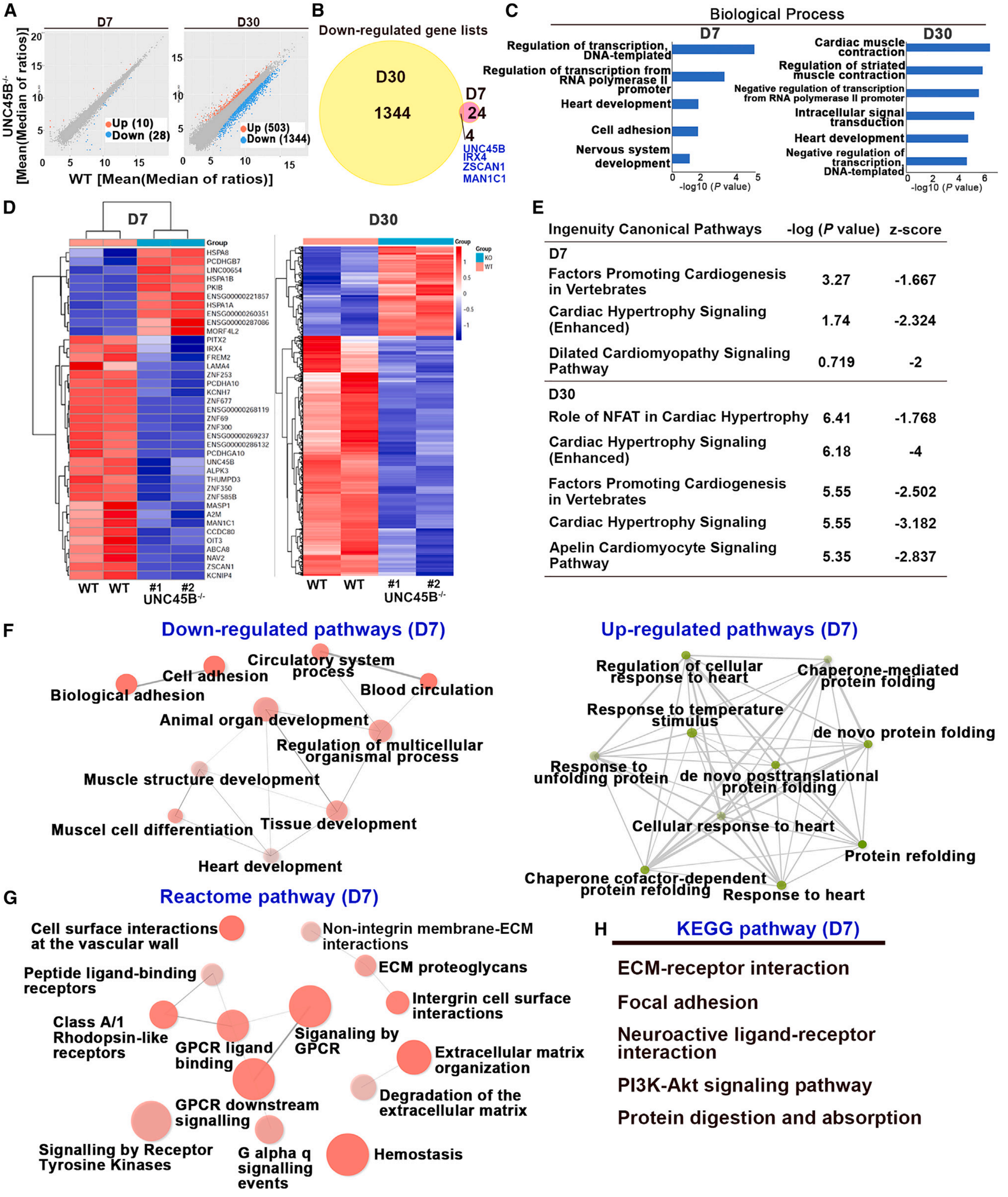

Figure 4. *UNC45B*-regulated genes mainly involved in heart development, extracellular matrix organization and focal adhesion
(A) Scatterplot illustrating the DEGs based on RNA sequencing results from day 7 and 30 WT-CMs and UNC45B$^{-/-}$-CMs. Up-regulated and down-regulated genes (adjusted $p < 0.05$) in UNC45B$^{-/-}$-CMs relative to WT-CMs are shown in orange and blue, respectively.

(legend continued on next page)

qPCR and corroborated that ablation of UNC45B did not affect the transcript levels of these tested sarcomere markers (Figure S4A).

UNC45B regulates protocostamere formation as well as myosin degradation to modulate myofibrillogenesis

We sought to elucidate the molecular mechanism by which UNC45B regulates sarcomere assembly. Hence, we isolated the RNAs of MYH6:mCherry-positive cells at differentiation day 7 and day 30 from both WT-CMs and UNC45B$^{-/-}$-CMs, and then carried out RNA sequencing (RNA-seq). In Figure 4A, we present a scatterplot of genes either 1.5-fold up- or down-regulated (adjusted $p < 0.05$) in UNC45B$^{-/-}$-CMs relative to WT-CMs. We detected 503 and 1344 genes that are either up- or down-regulated by *UNC45B* at day 30, respectively. However, much fewer genes were affected by *UNC45B* at early-stage day 7 (28 down-regulated and 10 up-regulated genes). We further performed Venn diagram analysis on those day 7 and day 30 down-regulated gene lists and found that four genes (*UNC45B*, *IRX4*, *ZSCAN1*, and *MAN1C1*) were down-regulated at both stages (Figure 4B). Gene Ontology (GO) analysis revealed that these down-regulated genes are involved in heart development in both day 7 and day 30 UNC45B$^{-/-}$-CMs (Figure 4C). Additionally, day 7 down-regulated genes are also involved in regulation of transcript from RNA polymerase II, heart development, and cell adhesion (Figure 4C, left panel), whereas day 30 down-regulated genes are predominantly involved in cardiac muscle contraction, intracellular signal transduction, the Wnt signaling pathway, and regulation of striated muscle contraction, among other terms (Figure 4C, right panel). Next, to further validate the RNA-seq data, we selected multiple genes from different GO categories of day 7 RNA-seq and examined them using qPCR. These genes include (1) heart development (*FREM2*, *ALPK3*, *IRX4*), (2) cell adhesion (*FREM2*, *LAMA4*, *PCDHGA10*, and *PCDHA10*), and (3) regulation of transcript from RNA polymerase II promoter (*IRX4* and *PITX4*). Consistently, these selected genes are significantly down-regulated in day 7 UNC45B$^{-/-}$-CMs (Figure S4B). Moreover, the heatmap in Figure 4D summarizes that *UNC45B* deletion mainly causes the majority of affected genes to be down-regulated. To further understand the UNC45B regulatory network at the early differentiation stage, we selected genes that are 1.5-fold up- and down-regulated ($p < 0.05$) from our day 7 and day 30 RNA-seq datasets and performed Ingenuity Pathway Analysis (IPA) (Figure 4E). Interestingly, we found that UNC45B-regulated genes in both day 7 and day 30 UNC45B$^{-/-}$-CMs are involved in (1) factors that promote cardiogenesis in vertebrates and (2) signaling pathways related to cardiac hypertrophy and dilated cardiomyopathy (Figure 4E). In addition to cardiac-related pathways, UNC45B-regulated genes (day 7) also participate in the GP6 signaling pathway, cAMP-mediated signaling, HIF1 signaling, and the BAG2 signaling pathway, among others (Table S2). Additionally, gene enrichment network analysis on day 7 differentially expressed genes (DEGs) further revealed that these DEGs are involved in both down-regulated pathways (e.g., biological and cell adhesion, circulatory system process, muscle structure development, and heart development) (Figure 4F, left panel) and up-regulated pathways (e.g., chaperone-mediated protein folding, response to unfolded protein, and protein refolding) (Figure 4F, right panel). Moreover, Kyoto Encyclopedia of Genes and Genomes (KEGG) enrichment network analysis and reactome pathway analysis uncovered that these DEGs (day 7) are involved in extracellular matrix organization, focal adhesion, GPCR signaling, and integrin cell surface interactions (Figures 4G and 4H). Taken together, these gene regulatory networks corroborate that UNC45B is involved in cardiogenesis and the extracellular cell matrix (focal adhesion), consistent with the protocostamere phenotypes displayed by CMs upon UNC45B knockout.

Next, to determine the binding partners of UNC45B during sarcomere assembly, we conducted co-immunoprecipitation (co-IP) coupled with mass spectrometry (MS). Among them were a number of well-known UNC45B binding partners (e.g., MYH6, HSP90AA1, HSP90AB1 and HSP70) (Table S3), which were verified using western blotting (Figure 5A), thereby validating our co-IP/MS analysis. IF staining further confirmed co-localization of

(B) Venn diagram analysis of UNC45B-regulated genes on the basis of day 7 (pink circle) and day 30 (yellow circle) RNA-seq datasets.
(C) Gene Ontology (GO) biological process analyses of down-regulated genes in day 7 and day 30 UNC45B$^{-/-}$-CMs.
(D) Hierarchical clustering analysis of the DEGs from day 7 and day 30 RNA-seq datasets. Scale bar represents the Z score value. For RNA-seq, WT-CMs (n = 2), and UNC45B$^{-/-}$-CMs (n = 2).
(E) Ingenuity canonical pathway analysis of DEGs from day 7 and 30 RNA-seq datasets. Gene lists from up-regulated and down-regulated genes ($p < 0.05$) in UNC45B$^{-/-}$-CMs relative to WT-CMs.
(F) DEG enrichment network analysis of day 7 RNA-seq data reveals that these DEGs are involved in both down-regulated pathways and up-regulated pathways.
(G and H) Reactome (G) and KEGG (H) pathway analysis of day 7 RNA-seq datasets.
Note that gene lists for (E)–(H) are from fold change (F.C.) > 1.5 up-regulated and down-regulated genes ($p < 0.05$) in UNC45B$^{-/-}$-CMs relative to WT-CMs. See also Figure S4.

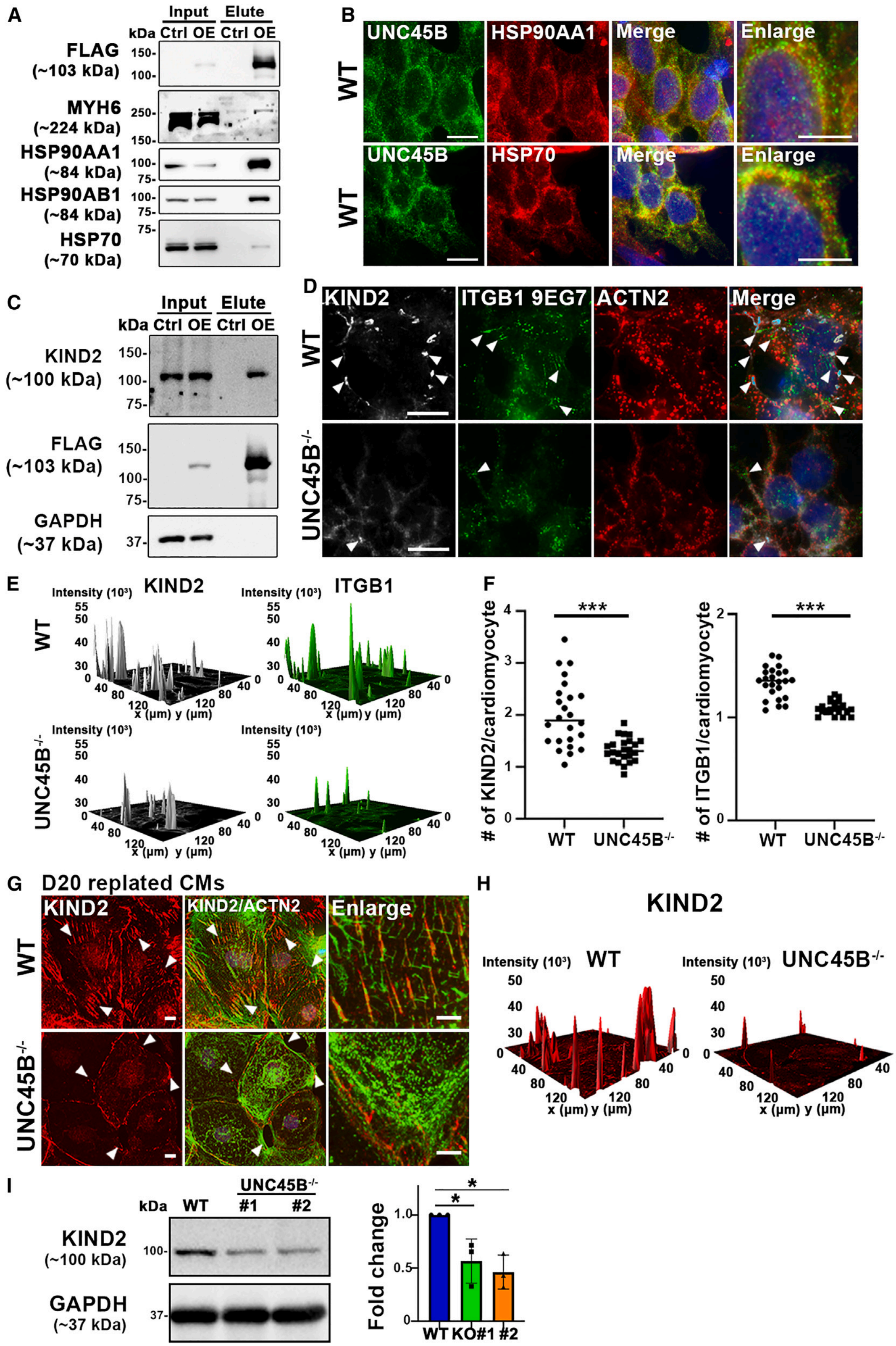

(legend on next page)

UNC45B with MYH6, HSP90AA1, and HSP70 (Figures 1H and 5B).

Apart from these known UNC45B binding partners, our co-IP/MS analysis also identified the protocostamere marker KIND2 (Table S3). Western blotting of our UNC45B pull-down samples with KIND2 and other protocostamere markers (i.e., ITGB1, vinculin [VCL] and paxillin [PXN]) revealed that only KIND2 interacts with UNC45B (Figure 5C), and not the other protocostamere markers (Figure S5A). Strikingly, IF staining revealed that expression of all these protocostamere markers (KIND2, ITGB1, VCL, and PXN) in nucleation sites was substantially reduced in UNC45B$^{-/-}$-CMs relative to WT-CMs at day 7 (non-replated) and day 20 (replated) (Figures 5D–5H and S5B–S5F). As KIND2 strongly co-localizes with UNC45B at early differentiation day 5 (i.e., the premyofibril stage), we wanted to examine KIND2 expression in our *UNC45B*-knockout lines using western blot. We found that levels of KIND2 were significantly reduced in UNC45B$^{-/-}$-CMs relative to WT-CMs (Figure 5I). Although co-localization of other protocostamere markers (VCL, PXL, and ITGB1) with sarcomere nucleation sites was significantly diminished, as revealed by immunostaining analysis, the protein levels of those protocostamere markers were not significantly different between WT-CMs and UNC45B$^{-/-}$-CMs (Figure S5G). Thus, we propose that UNC45B is involved in protocostamere formation and that it modulates KIND2 expression levels. To further demonstrate that KIND2 expression levels modulate UNC45B-mediated protocostamere formation, we ectopically expressed KIND2 in a UNC45B$^{-/-}$ line (hereafter denoted UNC45B$^{-/-:KIND2\ OE}$) (Figure 6A). Notably, IF staining of KIND2 and MYH10 revealed their strong co-localization in both WT and UNC45B$^{-/-:KIND2\ OE}$ CMs at day 7, indicating that the protocostamere-defective phenotype can be rescued by overexpressing KIND2 in the UNC45B$^{-/-}$ line (Figure 6B). Next, we wanted to determine if ACTN2 accumulation and a failure of F-ACTIN polymerization in the UNC45B$^{-/-}$ line are attributable to ACTN2 not being able to anchor to protocostameres. Importantly, IF staining of ACTN2 and F-ACTIN displayed filamentous-like structures in day 9 (not replated) and day 30 (replated) UNC45B$^{-/-:KIND2\ OE}$-CMs (Figures 6C, 6D, and S6), supporting that ACTN2 accumulation and failed F-ACTIN polymerization are associated with the protocostamere deficiency.

Ectopic expression of UNC45B does not affect sarcomere structure

Previous studies have shown that UNC45B overexpression in zebrafish and *C. elegans* disrupts myosin thick filament and enhances myosin degradation (Bernick et al., 2010; Landsverk et al., 2007; Ni et al., 2011). In order to determine if ectopic expression of UNC45B affects human sarcomere assembly, we overexpressed UNC45B in WT (denoted WT^{UNC45B}) and UNC45B$^{-/-}$ (denoted UNC45B$^{-/-:OE}$) hESC-CMs (Figure 7A). First, we performed IF staining for sarcomere markers (ACTN2, TNNT2, MYH6, and F-ACTIN) to examine the structure of sarcomeres in WT^{UNC45B}-CMs. In contrast to previous observations for zebrafish and *C. elegans*, we detected well-organized sarcomeric structures in the WT^{UNC45B}-CMs (Figure 7B), indicating that ectopic UNC45B in hESCs does not affect their sarcomere structures. However, ectopic UNC45B overexpression in a UNC45B$^{-/-}$ mutant line did rescue its knockout phenotypes, as the UNC45B$^{-/-:OE}$-CMs displayed beating contractility equivalent to WT (Video S2)

Figure 5. UNC45B modulates protocostamere formation by regulating KIND2 expression
(A) Co-IP assay showing the association of UNC45B-FLAG with MYH6 and heat shock proteins, as detected using western blots. Cell lysates of WT (negative control) and WT$^{UNC45B-OE}$-CMs (input) and pull-down samples using anti-FLAG beads (elute) were analyzed using SDS-PAGE. Anti-FLAG antibody was used to validate the co-IP assay. WT-CMs acted as a negative control.
(B) Representative IF images of WT-CMs harvested at day 7 and stained with antibodies for UNC45B and HSP90AA1 or HSP70. Scale bars: 10 μm. Enlargement scale bars: 5 μm. Results represent data from at least three independent experiments.
(C) Western blot following UNC45B-FLAG and KIND2 pull-down assay.
(D) Representative IF images of WT-CMs and UNC45B$^{-/-}$-CMs harvested at day 7 and stained for KIND2, ITGB1 9EG7, and ACTN2. Nucleation sites are indicated by arrowheads. Scale bars: 10 μm.
(E) Representative fluorescence intensity three-dimensional (3D) surface plots of KIND2 and ITGB1 9EG7 signals in day7 WT-CMs and UNC45B$^{-/-}$ CMs, illustrating protocostamere sites.
(F) Quantification results of numbers of KIND2 and ITGB1 nucleation sites per CM in WT-CMs and UNC45B$^{-/-}$-CMs (KIND2, n = 24; ITGB1, n = 24).
(G) Representative IF images of replated WT-CMs and UNC45B$^{-/-}$-CMs at day 20 and stained for KIND2 and ACTN2. Protocostamere sites are indicated by arrowheads. Scale bars: 10 μm. Enlargement scale bars: 5 μm.
(H) Representative fluorescence intensity 3D surface plots of KIND2 signal in replated WT-CMs and UNC45B$^{-/-}$-CMs at day 20.
(I) Western blots and the corresponding quantification data for KIND2 signal in WT-CMs and UNC45B$^{-/-}$-CMs at day 20. Error bars represent SD. Statistical significance is indicated: $^{*}p < 0.05$ and $^{***}p < 0.001$. Results represent data from at least three independent experiments. See also Figure S5.

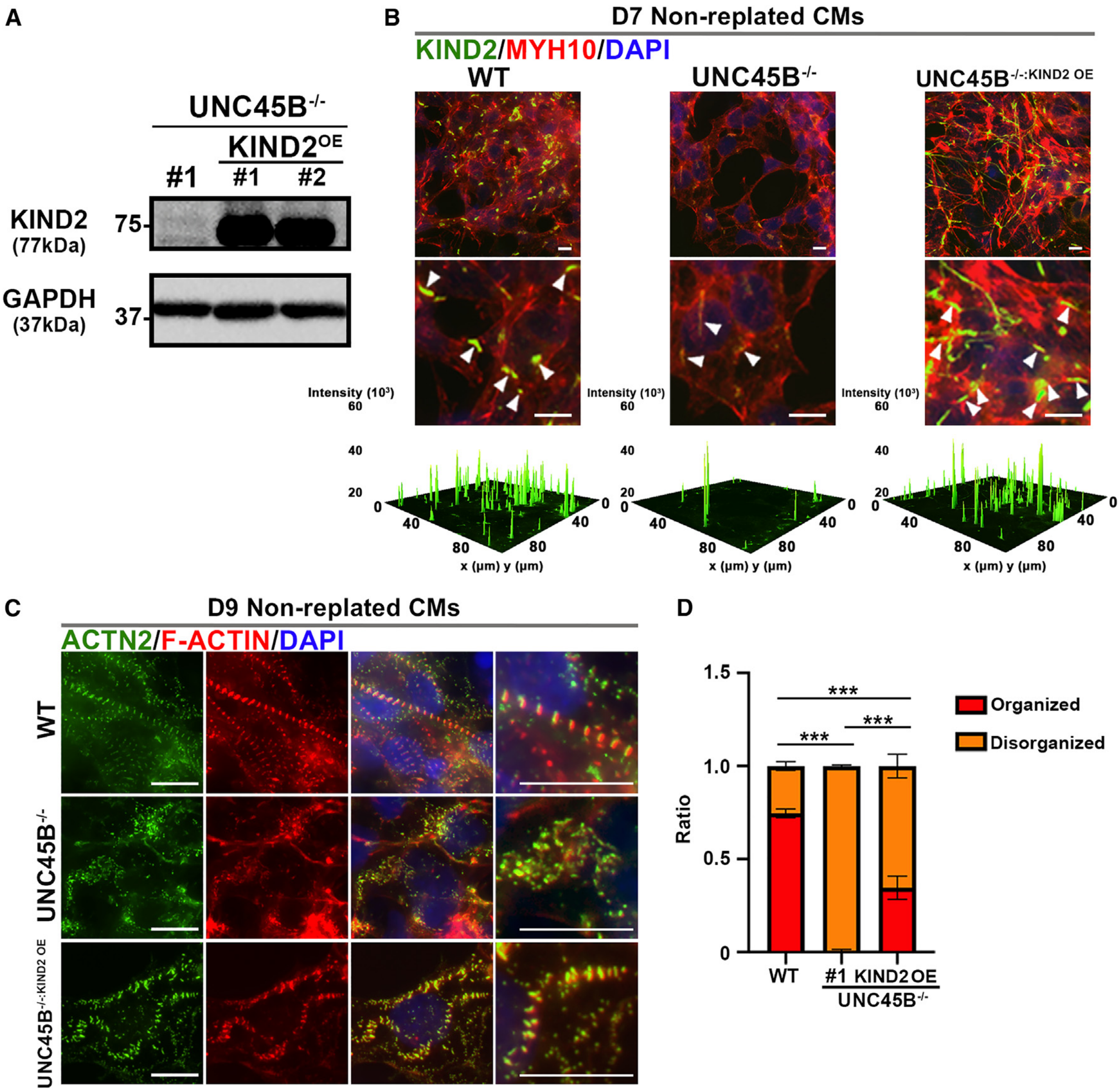

Figure 6. Ectopic expression of full-length KIND2 in a UNC45B$^{-/-}$ line rescues the protocostamere phenotype

(A) Western blots of day 7 UNC45B$^{-/-}$-CMs and UNC45B$^{-/-:KIND2\ OE}$-CMs.

(B) Representative IF images of non-replated WT, UNC45B$^{-/-}$-CMs, and UNC45B$^{-/-:KIND2\ OE}$-CMs harvested at day 8 and stained for KIND2 and MYH10. Representative fluorescence intensity 3D surface plots of KIND2 signal in non-replated WT-CMs, UNC45B$^{-/-}$-CMs, and UNC45B$^{-/-:KIND2\ OE}$-CMs. Note that KIND2 and MYH10 IF signals strongly overlap in both WT-CM and UNC45B$^{-/-:KIND2\ OE}$-CMs.

(C) Representative IF images of non-replated WT, UNC45B$^{-/-}$-CMs and UNC45B$^{-/-:KIND2\ OE}$-CMs harvested at day 9 and stained for ACTN2 and F-ACTIN.

(D) Rescue efficiency was quantified on the basis of ACTN2 staining. Note that ~34.7% ± 3.62% of ACTN2 accumulation was rescued in the UNC45B$^{-/-:KIND2\ OE}$-CMs. Results represent data from at least three independent experiments. Scale bars: 10 μm.

See also Figure S6.

and TEM analysis revealed well-organized sarcomeric structures (Figure 7C, lower panel).

Taken together, our findings demonstrate that (1) ablation of *UNC45B* impairs protocostamere formation (representing the initiating sites of myofibrillogenesis), so Z bodies (ACTN2, TTN, F-ACTIN) cannot bind to protocostameres to form mature Z lines and actin thin filament cannot be polymerized, and (2) *UNC45B* knockout perturbs myosin folding, so MYH6 cannot replace NMM MYH10, arresting myofibrillogenesis at an early stage and eliciting disrupted sarcomeres displaying filamentous-like structures.

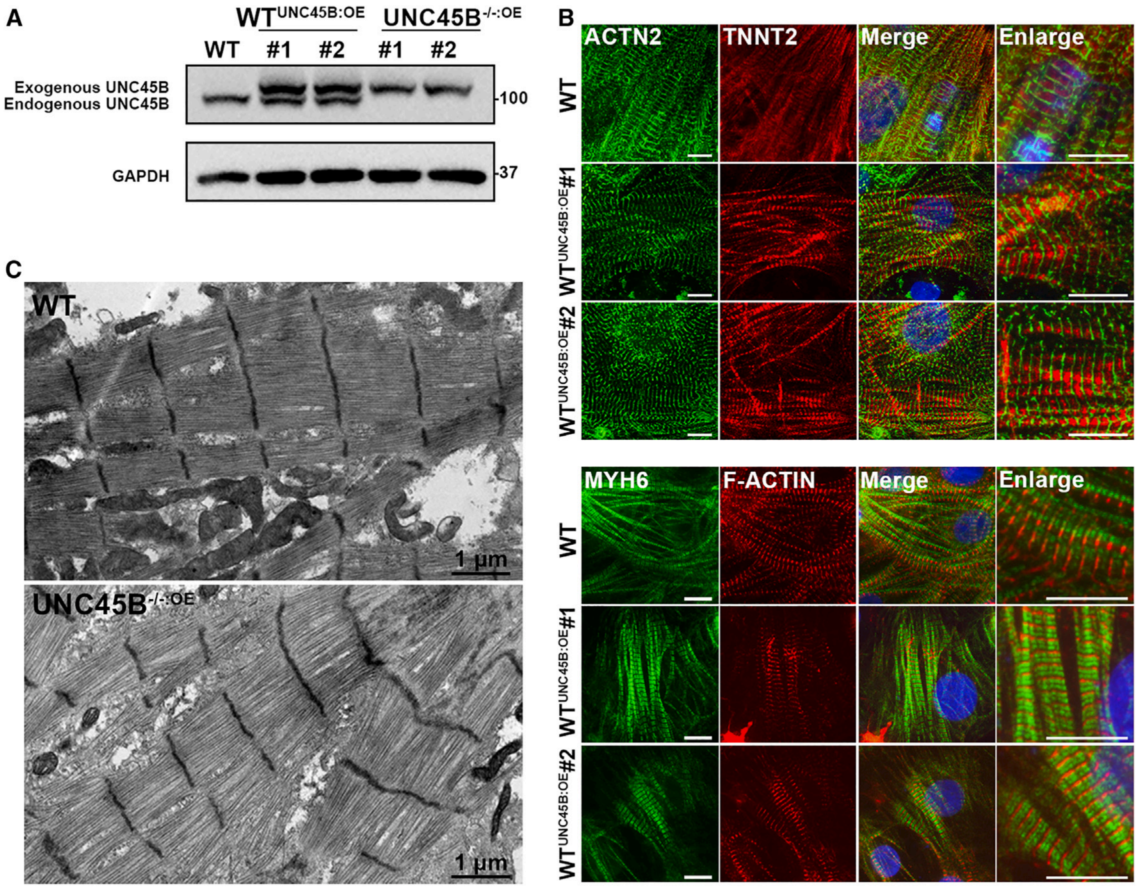

Figure 7. Effect of ectopic UNC45B expression in WT-CMs and UNC45B$^{-/-:}$-CMs

(A) Western blots of the protein expression levels of UNC45B in WT-CMs, WT$^{UNC45B\text{-}OE}$-CMs, and UNC45B$^{-/-:OE}$-CMs. Exogenous and endogenous UNC45B are indicated.

(B) Representative TEM images of WT-CMs and UNC45B$^{-/-:OE}$-CMs. Scale bars are indicated.

(C) Representative IF images of replated WT-CMs and WT$^{UNC45B\text{-}OE}$-CMs harvested at day 30 and stained for the ACTN2 and TNNT2 (upper panel) or for MYH6 and F-ACTIN (bottom panel). Scale bars: 10 μm. Results represent data from at least three independent experiments.

DISCUSSION

In this study, we first used hESC-CMs as a model to elucidate the spatiotemporal expression of core myofibrillogenesis markers (ACTN2, TTN, MYH6, MYH10, and F-ACTIN), protocostameric markers (ITGB1 and KIND2) and a molecular chaperone (UNC45B). We propose a model that sarcomere assembly starts from directed cardiac differentiation at day 5 when (1) protocostameres form as initial sites of cardiac myofibrillogenesis; (2) NMM MYH10 is highly expressed and co-localizes with the protocostamere marker KIND2; (3) ACTN2, TTN-N, and F-ACTIN form Z bodies (representing Z line precursors), and expression of muscle myosin MYH6 and UNC45B begins; (4) then, on day 7, F-ACTIN polymerizes to form actin thin filaments, and muscle myosin MYH6 starts to replace NMM MYH10; and, ultimately, (5) on day 10, ACTN2, TTN, MYH6, F-ACTIN, and UNC45B display a filamentous morphology and form sarcomeres.

Intriguingly, UNC45B co-localized with both KIND2 and MYH6. We ablated *UNC45B* from hESCs and found that doing so impaired protocostamere formation, so that the Z line anchor protein ACTN2 could not bind to protocostameres, resulting in ACTN2 accumulation and F-ACTIN polymerization in UNC45B$^{-/-}$-CMs. We have further demonstrated that UNC45B mediates protocostamere formation by regulating expression of KIND2.

The dynamic process of sarcomere assembly has been characterized previously using immunohistochemical analyses on non-human CMs (Rhee et al., 1994; Schultheiss et al., 1990). Moreover, because of the limited proliferative ability of human CMs, our knowledge of the process of cardiac myofibrillogenesis remains incomplete. Chopra et al. performed elegant experiments using human iPSC

(hiPSC)-CMs as a model and showed that human cardiac sarcomere assembly starts at a site of cell-matrix adhesion (known as the protocostamere) (Chopra et al., 2018). In order to observe the process of sarcomerogenesis, in that study they replated day 30 hiPSC-CMs to trigger sarcomere re-assembly and demonstrated that β-cardiac myosin-titin-protocostameres form a mechanical connection required for sarcomere assembly (Chopra et al., 2018). We recently established a protocol for culturing hESCs on Matrigel-coated cover slides, enabling direct harvesting of hESC-CMs at different time points of directed cardiac differentiation (Lu et al., 2022). In this study, our non-replated hESC-CM model enabled us to recapitulate both the dynamic spatiotemporal expression pattern and interactions between sarcomeric proteins during the human sarcomere assembly process. Moreover, our approach has further addressed fundamental questions regarding sarcomere assembly. First, we show that sarcomeres start to assemble at day 5 of directed cardiac differentiation, representing a cardiac progenitor stage. Second, though previous studies have shown that TTN cannot be recognized until after thick filament assembly (Fulton and Alftine, 1997), we found that both TTN-N and TTN-C could be detected at the same stage of differentiation (day 5), albeit at low expression levels and with punctate signal patterns. Third, we reveal that UNC45B is also expressed in the protocostamere area. Taken together, we have uncovered that sarcomere assembly starts at the cardiac progenitor stage of cardiac differentiation and also revealed differences between humans and other species in terms of the spatiotemporal expression patterns of sarcomeric proteins during sarcomere assembly.

Recently, we showed that aberrant splicing of ACTN2 or TTN disrupts ACTN2-TTN interaction, resulting in MYH6 accumulation around the nucleus. Consequently, MYH6 cannot replace MYH10 (Lu et al., 2022). Indeed, we observed that TTN-N co-localizes with ACTN2 and forms Z bodies at day 5. Moreover, Chopra et al. also showed that truncated TTN in A bands and ACTN2 lacking its C terminus cannot bind to cardiac myosin and TTN, respectively, resulting in significantly impaired sarcomere assembly (Chopra et al., 2018). Taken together, these data suggest that the mechanical force generated by protocostamere-ACTN2-TTN-MYH6 assembly may account for MYH6-MYH10 replacement.

UNC45B can be dynamically translocated in the cytoplasm as sarcomere development progresses and it overlaps with various markers, shuttling between the cytoplasm, A bands (myosin thick filament), and Z lines (Etard et al., 2008). Accordingly, diffuse expression patterns and diverse functions of UNC45B during myosin assembly have been observed and postulated previously (Barral et al., 1998; Bernick et al., 2010; Chen et al., 2012; Landsverk et al., 2007). However, multiple lines of evidence suggest that UNC45B also performs critical functions in early cardiac myofibrillogenesis: (1) knockout of *UNC45* in *C. elegans* causes embryonic lethality at a stage corresponding to the onset of myofibrillogenesis (Venolia and Waterston, 1990); (2) UNC45 co-localizes with NMM and integrin adhesion sites at developing sarcomeric Z lines in *Drosophila*, implying that UNC45 and NMM are involved in the formation of cell-matrix attachment complexes (Lee et al., 2011); and (3) zebrafish *unc45b* mutants display delayed nucleation of α-actinin and mislocalization of NMM (Myhre et al., 2014). Our findings are consistent with UNC45B being involved in early cardiac myofibrillogenesis. Importantly, we demonstrate that UNC45B binds to the KIND2 and mediates its expression, facilitating proper protocostamere formation, and thus provide a mechanistic model for early cardiac myofibrillogenesis. Interestingly, knockdown/knockout of KIND2 in zebrafish and mouse ESCs results in a failure of myofibril attachment to the protocostamere, as well as reduces ITGB1 activity (Dowling et al., 2008; Zhang et al., 2019) corroborating the function of KIND2 in the protocostamere.

Moreover, in the zebrafish model, Myhre et al. showed that unc45b mediated costamere formation by regulating NMM expression (Myhre et al., 2014). However, in our study, we found that ablation of UNC45B enhanced MYH10 expression. Moreover, ablation of MYH10 from hiPSC-CMs did not affect sarcomere assembly (Chopra et al., 2018), further indicating that MYH10 is important for zebrafish sarcomere assembly, but not critical for that process in human.

Additionally, knockdown of zebrafish *Unc45b* causes Z line disorganization (Hawkins et al., 2008). Moreover, Unc45b deficiency in *Xenopus* results in delayed polymerization of Z disc formation (Geach and Zimmerman, 2010), implying that it is required for Z line organization. Here, we have also revealed an accumulation of Z lines in UNC45B$^{-/-}$-CMs. However, ACTN2 is still able to bind to the TTN-N and F-ACTIN, enabling Z body formation. Importantly, ectopic expression of KIND2 in UNC45B-knockout line rescued protocostamere-deficient phenotypes, so ACTN2 can consequently form filamentous-like structures and F-ACTIN can be polymerized. Therefore, we have demonstrated that ACTN2 accumulation is due to a deficiency of protocostamere formation. Consequently, ACTN2 cannot anchor to protocostameres and establish the nucleation sites where ACTN2 and F-ACTIN assemble into the sarcomeric structure.

Additionally, even though UNC45B strongly co-localized with MYH10, ablation of UNC45B actually enhanced MYH10 expression. Although MYH10 knockout in mice elicits embryonic lethality (Tullio et al., 1997), ablation of

MYH10 from hiPSC-CMs did not affect sarcomere assembly (Chopra et al., 2018), indicating that MYH10 may not be critical for human sarcomere assembly.

Moreover, even though UNC45B did not affect the transcript levels of sarcomere markers (e.g., *MYH6*, *MYH10*, *ACTN2*, *KIND2*), we found that 28 genes are down-regulated in day 7 UNC45B$^{-/-}$-CMs. Interestingly, many of these genes perform CM-related functions: (1) *IRX4* has been shown involved in heart development by regulating expression of chamber-specific genes, and the loss of *Irx4* in mice results in cardiac decompensation (Kim et al., 2012). (2) PITX2 plays crucial roles in inner curvature remodeling and ventricular chamber expansion (Tessari et al., 2008). (3) *ALPK3* regulates heart development (Jorholt et al., 2020). Moreover, ALPK3 together with UNC45B and KIND2 and have been identified in the N-cadherin interactome (Li et al., 2019), suggesting potential interactions among these three proteins at the costamere. (4) *FREM2* mutants led to septa and valve malformations (Hardell et al., 1984). (5) *PCDHA10* is expressed predominantly in the atria of the heart and mutations in the mouse PCDHA10 ortholog (PCDHA9) resulted in bicuspid aortic valve (Zhu et al., 2022). (6) *LAMA4* is highly abundant in the heart, and mutations in this gene are associated with dilated cardiomyopathy (Wang et al., 2006).

Notably, RNA-seq data revealed an altered expression in a larger number of genes in d30 (1,847 up- and down-regulated genes) compared with that in day 7 (38 up- and down-regulated genes) in the UNC45B$^{-/-}$-CMs. As a mechanosignaling hub linking the sarcomere and extracellular matrix, the protocostamere deficiency could lead to abnormal transcriptomic regulation (Henderson et al., 2017). It is possible that the DEGs observed in RNA-seq data are attributed to direct or indirect effects of UNC45B ablation. Thus, in addition to functioning as a molecular chaperone, UNC45B can also modulate expression of genes conducting CM-related functions and it would be interested to further investigate in the future.

In conclusion, we used hESCs to reveal the spatiotemporal process of cardiac myofibrillogenesis. Moreover, we have uncovered the function of UNC45B in human sarcomere assembly and demonstrated that UNC45B mediates KIND2 to form protocostameres, representing the initial sites of sarcomere assembly.

EXPERIMENTAL PROCEDURES

Resource availability

Corresponding author

Requests for further information or more detailed protocols should be directed to and will be fulfilled by the corresponding author, Su-Yi Tsai (suyitsai@ntu.edu.tw).

Materials availability

This study did not generate new unique reagents.

Data and code availability

RNA-seq data are available from the Gene Expression Omnibus (GEO) repository database (GSE229229). MS data are available from the ProteomeXchange database (PXD042075). Raw data and images are available upon request to the corresponding author.

Cell culture

Human H9 ESCs and directed cardiac differentiation were cultured and performed as previously described (Lu et al., 2022). For further information on generation of knockout hESC lines, overexpression of UNC45B and KIND2 in UNC45B-knockout or WT hESC lines, see supplemental experimental procedures.

IF analysis, western blotting, fluorescence-activated cell sorting, flow cytometry, real-time qPCR, transmission electron microscopy, and RNA isolation, sequencing, and analyses

These methods were performed as previously described (Lu et al., 2022). For the detailed information, see supplemental experimental procedures.

Co-IP and shotgun proteomic identifications by MS

WT-CMs and UNC45B-FLAG-CMs were cultured in two 10 cm dishes until they attained ~90% confluency and then harvested using Accutase. Pellets were washed with 1x PBS and resuspended in ice-cold lysis buffer (50 mM Tris-HCl [pH 7.5], 1 mM EDTA, 150 mM NaCl, 1% Triton X-100, 10% glycerol, 1 mM PMSF) with protease inhibitor cocktail (Roche). For the detailed information, see supplemental experimental procedures.

Statistics

Statistical analyses were conducted in Prism (version 7). To test data normality, a Shapiro-Wilk test was performed. For normally distributed data, a parametric two-tailed t tests (2-group data) or one-way ANOVA (>2-group data) followed by Tukey's post hoc test was used to calculate statistical significance. For data that failed the normality test, a Mann-Whitney test (2-group data) or Kruskal-Wallis test (>2-group data) followed by Dunn's post hoc test was performed. The notation for p values is as follows: *$p < 0.05$, **$p < 0.01$, and ***$p < 0.001$.

SUPPLEMENTAL INFORMATION

Supplemental information can be found online at https://doi.org/10.1016/j.stemcr.2023.05.006.

AUTHOR CONTRIBUTIONS

S.H.-A.L., Y.-H.W., L.-Y.S., Z.-T.H., T.-H.W., H.-Y.W., and C.Y., design, collection, and analysis of data; H.-Y.W., performance of IPA; S.-Y.T., conception and design of experiments, data collection and analyses, and manuscript writing.

ACKNOWLEDGMENTS

We thank Dr. Kuo-Chiang Hsia for his valuable discussions and critical review of this article. We appreciate the support of the Flow Cytometry Core Facility, Imaging Core, and the Bioinformatics Core at the Institute of Molecular Biology, Academia Sinica. We acknowledge the service provided by the Flow Cytometric Analyzing and Sorting Core Facility at National Taiwan University Hospital and MS analysis by Proteomics Mass Spectrometry Common Facility at the Institute of Biological Chemistry, Academia Sinica (AS-CFII-111-209). We also thank John O'Brien for manuscript editing and the Technology Commons, College of Life Science, National Taiwan University, for technical support. This work was supported by National Taiwan University (108L7868, 111L104010, and 111L891105 to S.-Y.T.) and the National Science and Technology Council, Taiwan (NSTC 109-2314-B-002-087-MY3 and 111-2628-B-002 -027 -MY3 to S.-Y.T.).

CONFLICTS OF INTEREST

The authors declare no competing interests.

Received: October 26, 2022
Revised: May 9, 2023
Accepted: May 9, 2023
Published: June 8, 2023

REFERENCES

Barral, J.M., Bauer, C.C., Ortiz, I., and Epstein, H.F. (1998). Unc-45 mutations in Caenorhabditis elegans implicate a CRO1/She4p-like domain in myosin assembly. J. Cell Biol. *143*, 1215–1225.

Barral, J.M., Hutagalung, A.H., Brinker, A., Hartl, F.U., and Epstein, H.F. (2002). Role of the myosin assembly protein UNC-45 as a molecular chaperone for myosin. Science *295*, 669–671. https://doi.org/10.1126/science.1066648.

Bernick, E.P., Zhang, P.J., and Du, S. (2010). Knockdown and overexpression of Unc-45b result in defective myofibril organization in skeletal muscles of zebrafish embryos. BMC Cell Biol. *11*, 70. https://doi.org/10.1186/1471-2121-11-70.

Chen, D., Li, S., Singh, R., Spinette, S., Sedlmeier, R., and Epstein, H.F. (2012). Dual function of the UNC-45b chaperone with myosin and GATA4 in cardiac development. J. Cell Sci. *125*, 3893–3903.

Chopra, A., Kutys, M.L., Zhang, K., Polacheck, W.J., Sheng, C.C., Luu, R.J., Eyckmans, J., Hinson, J.T., Seidman, J.G., Seidman, C.E., and Chen, C.S. (2018). Force generation via β-cardiac myosin, titin, and α-actinin Drives cardiac sarcomere assembly from cell-matrix adhesions. Dev. Cell *44*, 87–96.e5. https://doi.org/10.1016/j.devcel.2017.12.012.

Clark, K.A., McElhinny, A.S., Beckerle, M.C., and Gregorio, C.C. (2002). Striated muscle cytoarchitecture: an intricate web of form and function. Annu. Rev. Cell Dev. Biol. *18*, 637–706. https://doi.org/10.1146/annurev.cellbio.18.012502.105840.

Dowling, J.J., Gibbs, E., Russell, M., Goldman, D., Minarcik, J., Golden, J.A., and Feldman, E.L. (2008). Kindlin-2 is an essential component of intercalated discs and is required for vertebrate cardiac structure and function. Circ. Res. *102*, 423–431. https://doi.org/10.1161/circresaha.107.161489.

Epstein, H.F., and Thomson, J.N. (1974). Temperature-sensitive mutation affecting myofilament assembly in Caenorhabditis elegans. Nature *250*, 579–580.

Etard, C., Behra, M., Fischer, N., Hutcheson, D., Geisler, R., and Strähle, U. (2007). The UCS factor Steif/Unc-45b interacts with the heat shock protein Hsp90a during myofibrillogenesis. Dev. Biol. *308*, 133–143. https://doi.org/10.1016/j.ydbio.2007.05.014.

Etard, C., Roostalu, U., and Strähle, U. (2008). Shuttling of the chaperones Unc45b and Hsp90a between the A band and the Z line of the myofibril. J. Cell Biol. *180*, 1163–1175. https://doi.org/10.1083/jcb.200709128.

Fulton, A.B., and Alftine, C. (1997). Organization of protein and mRNA for titin and other myofibril components during myofibrillogenesis in cultured chicken skeletal muscle. Cell Struct. Funct. *22*, 51–58. https://doi.org/10.1247/csf.22.51.

Geach, T.J., and Zimmerman, L.B. (2010). Paralysis and delayed Z-disc formation in the Xenopus tropicalis unc45b mutant dicky ticker. BMC Dev. Biol. *10*, 75. https://doi.org/10.1186/1471-213x-10-75.

Hardell, L., Bengtsson, N.O., Jonsson, U., Eriksson, S., and Larsson, L.G. (1984). Aetiological aspects on primary liver cancer with special regard to alcohol, organic solvents and acute intermittent porphyria–an epidemiological investigation. Br. J. Cancer *50*, 389–397. https://doi.org/10.1038/bjc.1984.188.

Hawkins, T.A., Haramis, A.P., Etard, C., Prodromou, C., Vaughan, C.K., Ashworth, R., Ray, S., Behra, M., Holder, N., Talbot, W.S., et al. (2008). The ATPase-dependent chaperoning activity of Hsp90a regulates thick filament formation and integration during skeletal muscle myofibrillogenesis. Development *135*, 1147–1156. https://doi.org/10.1242/dev.018150.

Henderson, C.A., Gomez, C.G., Novak, S.M., Mi-Mi, L., and Gregorio, C.C. (2017). Overview of the muscle cytoskeleton. Compr. Physiol. *7*, 891–944. https://doi.org/10.1002/cphy.c160033.

Jorholt, J., Formicheva, Y., Vershinina, T., Kiselev, A., Muravyev, A., Demchenko, E., Fedotov, P., Zlotina, A., Rygkov, A., Vasichkina, E., et al. (2020). Two new cases of hypertrophic cardiomyopathy and skeletal muscle features associated with ALPK3 homozygous and compound heterozygous variants. Genes *11*, 1201. https://doi.org/10.3390/genes11101201.

Kim, K.H., Rosen, A., Bruneau, B.G., Hui, C.C., and Backx, P.H. (2012). Iroquois homeodomain transcription factors in heart development and function. Circ. Res. *110*, 1513–1524. https://doi.org/10.1161/circresaha.112.265041.

Landsverk, M.L., Li, S., Hutagalung, A.H., Najafov, A., Hoppe, T., Barral, J.M., and Epstein, H.F. (2007). The UNC-45 chaperone mediates sarcomere assembly through myosin degradation in Caenorhabditis elegans. J. Cell Biol. *177*, 205–210. https://doi.org/10.1083/jcb.200607084.

Lee, C.F., Melkani, G.C., Yu, Q., Suggs, J.A., Kronert, W.A., Suzuki, Y., Hipolito, L., Price, M.G., Epstein, H.F., and Bernstein, S.I. (2011). Drosophila UNC-45 accumulates in embryonic blastoderm and in muscles, and is essential for muscle myosin stability. J. Cell Sci. *124*, 699–705.

Li, Y., Merkel, C.D., Zeng, X., Heier, J.A., Cantrell, P.S., Sun, M., Stolz, D.B., Watkins, S.C., Yates, N.A., and Kwiatkowski, A.V. (2019). The N-cadherin interactome in primary cardiomyocytes as defined using quantitative proximity proteomics. J. Cell Sci. *132*, jcs221606. https://doi.org/10.1242/jcs.221606.

Lu, S.H.A., Lee, K.Z., Hsu, P.W.C., Su, L.Y., Yeh, Y.C., Pan, C.Y., and Tsai, S.Y. (2022). Alternative splicing mediated by RNA-binding protein RBM24 facilitates cardiac myofibrillogenesis in a differentiation stage-specific manner. Circ. Res. *130*, 112–129. https://doi.org/10.1161/circresaha.121.320080.

Myhre, J.L., Hills, J.A., Jean, F., and Pilgrim, D.B. (2014). Unc45b is essential for early myofibrillogenesis and costamere formation in zebrafish. Dev. Biol. *390*, 26–40. https://doi.org/10.1016/j.ydbio.2014.02.022.

Ni, W., Hutagalung, A.H., Li, S., and Epstein, H.F. (2011). The myosin-binding UCS domain but not the Hsp90-binding TPR domain of the UNC-45 chaperone is essential for function in Caenorhabditis elegans. J. Cell Sci. *124*, 3164–3173. https://doi.org/10.1242/jcs.087320.

Poon, K.L., Tan, K.T., Wei, Y.Y., Ng, C.P., Colman, A., Korzh, V., and Xu, X.Q. (2012). RNA-binding protein RBM24 is required for sarcomere assembly and heart contractility. Cardiovasc. Res. *94*, 418–427.

Price, M.G., Landsverk, M.L., Barral, J.M., and Epstein, H.F. (2002). Two mammalian UNC-45 isoforms are related to distinct cytoskeletal and muscle-specific functions. J. Cell Sci. *115*, 4013–4023.

Rhee, D., Sanger, J.M., and Sanger, J.W. (1994). The premyofibril: evidence for its role in myofibrillogenesis. Cell Motil Cytoskeleton *28*, 1–24. https://doi.org/10.1002/cm.970280102.

Sanger, J.W., Wang, J., Fan, Y., White, J., and Sanger, J.M. (2010). Assembly and dynamics of myofibrils. J. Biomed. Biotechnol. *2010*, 858606. https://doi.org/10.1155//858606.

Schultheiss, T., Lin, Z.X., Lu, M.H., Murray, J., Fischman, D.A., Weber, K., Masaki, T., Imamura, M., and Holtzer, H. (1990). Differential distribution of subsets of myofibrillar proteins in cardiac nonstriated and striated myofibrils. J. Cell Biol. *110*, 1159–1172. https://doi.org/10.1083/jcb.110.4.1159.

Sparrow, J.C., and Schöck, F. (2009). The initial steps of myofibril assembly: integrins pave the way. Nat. Rev. Mol. Cell Biol. *10*, 293–298. https://doi.org/10.1038/nrm2634.

Srikakulam, R., Liu, L., and Winkelmann, D.A. (2008). Unc45b forms a cytosolic complex with Hsp90 and targets the unfolded myosin motor domain. PLoS One *3*, e2137. https://doi.org/10.1371/journal.pone.0002137.

Tessari, A., Pietrobon, M., Notte, A., Cifelli, G., Gage, P.J., Schneider, M.D., Lembo, G., and Campione, M. (2008). Myocardial Pitx2 differentially regulates the left atrial identity and ventricular asymmetric remodeling programs. Circ. Res. *102*, 813–822. https://doi.org/10.1161/circresaha.107.163188.

Tsai, S.Y., Ghazizadeh, Z., Wang, H.J., Amin, S., Ortega, F.A., Badieyan, Z.S., Hsu, Z.T., Gordillo, M., Kumar, R., Christini, D.J., Evans, T., and Chen, S. (2020). A human embryonic stem cell reporter line for monitoring chemical-induced cardiotoxicity. Cardiovascular research *116*, 658–670. https://doi.org/10.1093/cvr/cvz148.

Tullio, A.N., Accili, D., Ferrans, V.J., Yu, Z.X., Takeda, K., Grinberg, A., Westphal, H., Preston, Y.A., and Adelstein, R.S. (1997). Nonmuscle myosin II-B is required for normal development of the mouse heart. Proc. Natl. Acad. Sci. USA *94*, 12407–12412. https://doi.org/10.1073/pnas.94.23.12407.

Venolia, L., Ao, W., Kim, S., Kim, C., and Pilgrim, D. (1999). unc-45 gene of Caenorhabditis elegans encodes a muscle-specific tetratricopeptide repeat-containing protein. Cell Motil Cytoskeleton *42*, 163–177. https://doi.org/10.1002/(sici)1097-0169(1999)42:3<163::Aid-cm1>3.0.Co;2-e.

Venolia, L., and Waterston, R.H. (1990). The unc-45 gene of Caenorhabditis elegans is an essential muscle-affecting gene with maternal expression. Genetics *126*, 345–353.

Wang, J., Hoshijima, M., Lam, J., Zhou, Z., Jokiel, A., Dalton, N.D., Hultenby, K., Ruiz-Lozano, P., Ross, J., Jr., Tryggvason, K., and Chien, K.R. (2006). Cardiomyopathy associated with microcirculation dysfunction in laminin alpha4 chain-deficient mice. J. Biol. Chem. *281*, 213–220. https://doi.org/10.1074/jbc.M505061200.

Zhang, Z., Mu, Y., Zhang, J., Zhou, Y., Cattaneo, P., Veevers, J., Peter, A.K., Manso, A.M., Knowlton, K.U., Zhou, X., et al. (2019). Kindlin-2 is essential for Preserving integrity of the developing heart and preventing ventricular rupture. Circulation *139*, 1554–1556. https://doi.org/10.1161/circulationaha.118.038383.

Zhu, W., Williams, K., Young, C., Lin, J.H., Teekakirikul, P., and Lo, C.W. (2022). Rare and Common variants uncover the role of the atria in coarctation of the aorta. Genes *13*, 636. https://doi.org/10.3390/genes13040636.

Stem Cell Reports, Volume *18*

Supplemental Information

Cardiac myofibrillogenesis is spatiotemporally modulated by the molecular chaperone UNC45B

Serena Huei-An Lu, Yi-Hsuan Wu, Liang-Yu Su, Zi-Ting Hsu, Tzu-Han Weng, Hsin-Yu Wang, Chiao Yu, Paul Wei-Che Hsu, and Su-Yi Tsai

Supplemental Information

Cardiac Myofibrillogenesis is Spatiotemporally Modulated by the Molecular Chaperone UNC45B

Serena Huei-An Lu[#], Yi-Hsuan Wu[#], Liang-Yu Su[#], Zi-Ting Hsu[#], Tzu-Han Weng, Hsin-Yu Wang, Chiao Yu, Paul Wei-Che Hsu, Su-Yi Tsai[*]

Supplementary Experimental Procedures

hESC culture and directed cardiac differentiation

Human H9 ESCs were purchased from WiCell. All hESCs used in this study were derived from the MYH6:mCherry reporter line(Tsai et al., 2020). hESCs were grown on a Matrigel (Corning, 354277)-coated plate in Essential 8 medium (Gibco, A1516901) supplemented with E8 Supplement (Gibco, A1517101) and Normocin (InvivoGen, ant-nr-2) at 37 °C in 5% CO_2. The culture medium was exchanged daily. We used 0.5 mM EDTA (Invitrogen, 15575038) for routine passaging of hESCs. Accutase (BD Biosciences, 561527) was used for single-cell dissociation. Directed cardiac differentiation was conducted as described previously(Lu et al., 2022). In brief, hESCs were cultured in hESC medium until they attained ~80-90% confluency. At differentiation day 0, hESCs were treated with 12 μM CHIR99021 (CHIR, Tocris Bioscience) in RPMI (Gibco, 11875085) supplemented with B27 minus insulin (Gibco, A1895601) for 24 h. CHIR was removed the next day. At day 3, differentiated cells were treated with 5 μM WNT antagonist I (IWR-1, Tocris Bioscience) for 2 days. IWR-1 was removed at day 5. At day 7, B27 minus insulin in cardiac differentiation medium was changed to RPMI-B27 (Gibco, 17504044). Beating cells were typically observed around day 6-7. Both the lactate metabolic-selection method(Tohyama et al., 2013) and fluorescence-activated cell sorting (FACS) were used to purify hESC-CMs.

Generation of UNC45B-knockout hESC lines using the CRISPR/Cas9 technique

Two sets of sgRNA sequences were designed using the website http://chopchop.cbu.uib.no. The knockout strategy was as described previously[11]. In brief, the sgRNA sequences were ligated into pX330-U6-Chimeric_BB-CBh-hSpCas9 vector (Addgene, plasmid #42230)(Cong et al., 2013; Ran et al., 2013). MYH6:mCherry hESCs were transfected by electroporation using an Amaxa Human Stem Cell Nucleofector Kit 2 (Lonza, VPH-5022) according to the manufacturer's guidelines. After electroporation, cells were re-plated on Matrigel-coated plates with 10 μM ROCK inhibitor (Tocris) at a low cell density (100 cells/well of a 6-well plate). After ~10 days, single clones were picked and separated into two wells of a 96-well plate. When the cells reached 80-90% confluency, we added lysis buffer (Sigma, Cat. #L3289) and neutralization buffer (Sigma, Cat. #N9784) to isolate genomic DNA for genotyping and DNA sequencing. All primers are listed in Table 1.

Overexpression of UNC45B and KIND2 in UNC45B-knockout or wild-type hESC lines

Human UNC45B or KIND2 coding regions were amplified by polymerase chain reaction (PCR) using hESC-CM cDNA as template and then cloned into the pCMV3flag8HOIL-1L vector (Addgene, #50016). The UNC45B-FLAG or KIND2-FLAG genes hosting FLAG tags were subcloned into lentivector pLKO AS3w.bsd. For virus production, 10 μg of target vector (UNC45B-FLAG), lentiviral envelope and the packaging plasmids pCMV-VSV-G (Addgene

#8454), psPAX2 (Addgene, #12260) and pMD2.G (Addgene, #12259) were all transfected into 293T cells by means of a standard $CaCl_2$ transfection method. After 72 h, lentiviral supernatants were collected and filtered through a 0.45 μm syringe filter. UNC45B-knockout and wild-type (WT) hESCs were infected with UNC45B-FLAG or KIND2-FLAG lentivirus using a spin infection method, as described previously[11]. After infection, the medium was replaced with E8 medium. Two days later, Blasticidin (6.67 μg/ml, Gibco, R21001) was used to select UNC45B-FLAG or KIND2-FLAG -positive clones.

Immunofluorescence analysis

Day 5 to 10 hESC-CMs were cultured on Matrigel-coated coverslides. To replate day15 to 30 hESC-CMs, the cells at day 10 to 25 were dissociated into single cells using TrypLE Express (Gibco 12604021) combined with Collagenase Type IV (Gibco, 17104019) for 10-15 min at 37 °C. The cells were centrifuged at 300 *g* for 4 min and replated on Matrigel-coated coverslides or on an Ibidi 96-well plate (Ibidi, IB-89626). Cells then were fixed with 4% PFA for 8 min at room temperature (RT). Blocking was performed in 5% horse serum (Gibco) and 0.3% Triton X-100 (93443-500ML, Sigma-Aldrich) in PBS for 1 h at RT, followed by primary antibody incubation overnight at 4 °C or for 2 h at RT. The following antibodies were used: mouse anti-α-Actinin (Sarcomeric) (A7811, Sigma-Aldrich); rabbit anti-α-Actinin (Sarcomeric) (710947, Invitrogen); mouse anti-MYH6 (MAB8979, R&D Systems); mouse or rabbit anti-TNNT2 (Invitrogen, MA5-12960; ab45932, Abcam); mouse or rabbit anti-MYH10 (GTX634160, GeneTex; GTX133378, GeneTex); rabbit anti-TTN-N (TTN-1, Myomedix); rabbit anti-TTN-C (TTN-9, Myomedix); rabbit anti-HSP70 (10995-1-AP, Proteintech); rabbit anti-HSP90AA1 (GTX109753, GeneTex); rabbit anti-HSP90AB1 (GTX101448, GeneTex); rabbit anti-ITGB1 (GTX128839, GeneTex); rat anti-ITGB1 9EG7 (553715, BD Biosciences); rabbit anti-KINDLIN2 (GTX118359, GeneTex); mouse anti-PAXILLIN (610051, BD Biosciences); goat or rabbit anti-UNC45B (GTX88214, GeneTex; PA5-53648, Invitrogen); mouse anti-VINCULIN (V9131, Sigma-Aldrich); and Fluor 647 PHALLOIDIN Conjugate (A22287, Invitrogen). Antibodies were detected with Alexa-488-, Alexa-555- and Alexa-647-conjugated donkey secondary antibodies against mouse, goat or rabbit (1:1000, Invitrogen). Nuclei were counterstained with DAPI. Images were obtained using a fluorescence microscope (Olympus IX83).

Western blotting

Cell pellets were lysed using RIPA buffer containing 1% PMSF. Protein concentration was measure by the BCA method using a Pierce BCA Protein Assay Kit (23225, Thermo Fisher Scientific). Samples were resolved on 9-13.3% sodium dodecyl sulfate polyacrylamide gels (SDS-PAGE), which were then transferred to PVDF membrane at 100v for 120 min. Membranes were blocked using 5% non-fat dry milk in 1xTBST at RT for 1 h and then incubated with primary antibody at 4 °C overnight. The following antibodies were used: goat or rabbit anti-

UNC45B (GTX88214, GeneTex; PA5-53648, Invitrogen); mouse or rabbit anti-α-ACTININ (Sarcomeric) (A7811, Sigma-Aldrich; 710947, Invitrogen); rabbit anti-DDDDK tag (GTX115043, GeneTex); rabbit anti-HSP90AA1 (GTX109753, GeneTex); rabbit anti-HSP90AB1 (GTX101448, GeneTex); rabbit anti-ITGB1 (GTX128839, GeneTex); rabbit anti-KINDLIN2 (13562, Cell Signaling); mouse MYH6 (MAB8979, R&D Systems); rabbit anti-MYH10 (GTX133378, GeneTex); mouse PAXILLIN (610051, BD Biosciences); mouse anti-VINCULIN (V9131, Sigma-Aldrich); rabbit anti-BETA-ACTIN (ab1801, abcam); rabbit anti-GAPDH (GTX100118, GeneTex); and mouse anti-ALPHA TUBULIN (GTX628802, GeneTex). After appropriate washing with 1xTBST, the membranes were incubated with HRP goat anti-mouse IgG (Jackson ImmunoResearch, 115-035-003) or HRP goat anti-rabbit IgG (GeneTex, GTX213110) at RT for 30 min. Chemiluminescent assay was performed using ECL substrates, and signals were detected with a FUSION Solo S chemiluminescence imaging system (Vilber).

FACS and flow cytometry

hESC-CMs were dissociated into single cells using TrypLE Express (Gibco 12604021) combined with Collagenase Type IV (Gibco, 17104019) for 10-15 min at 37 °C. Cells were centrifuged at 300 *g* for 4 min and resuspended in FACS buffer containing DMEM without phenol red (Life Technologies, 21063-029), 1 mM EDTA, 25 mM HEPES (Corning, 13116004), and 5% fetal bovine serum (FBS). MYH6:mCherry-positive cells were collected using a BD FACS Aria Cell Sorter (BD Bioscience). Flow cytometric analysis of cardiac differentiation efficiency using TNNT2-FITC (Santa Cruz, sc-20025) and MYH6:mCherry signals was performed on an Aurora (Cytek) system.

RNA isolation, sequencing and analyses

Total RNA was extracted using a RNeasy Mini Kit (Qiagen, 74106). RNA purity, concentration and integrity were inspected using a NanoPhotometer® spectrophotometer (IMPLEN, CA, USA), a Qubit® RNA Assay Kit in a Qubit® 2.0 Flurometer (Life Technologies, CA, USA), and an RNA Nano 6000 Assay Kit in a Bioanalyzer 2100 system (Agilent Technologies, CA, USA), respectively. The mRNA sequencing libraries were prepared using a NEBNext® UltraTM RNA Library Prep Kit for Illumina® (NEB, USA), and 150 bp paired-read sequencing was performed using an Illumina HiSeq instrument. Clean data (clean reads) were obtained by removing low-quality reads and reads containing adapters or poly-N from the raw data. Clean reads were mapped to the human genome assembly using HISAT2. Raw read count data was filtered using iDEP (integrated Differential Expression and Pathway analysis, http://bioinformatics.sdstate.edu/idep/) by removing genes with <0.5 counts per million (CPM) across all samples in each dataset. Differential gene expression analysis based on DESeq2 was performed using iDEP. The resulting P-values were adjusted using the Benjamini and

Hochberg's approach to control for the false discovery rate. Genes identified with an adjusted P-value of <0.05 and a fold-change >1.5 were assigned as being differentially expressed. Canonical pathways and functional analysis of the differentially expressed genes were evaluated using Ingenuity Pathway Analysis (IPA; Qiagen Inc.). Scatter plots, Venn diagrams and heatmaps of differentially expressed genes were plotted in R Statistical Software (v4.2.0; R Core Team 2022). Gene ontology (GO) analysis was performed using DAVID Bioinformatics Resources v6.8 (https://david.ncifcrf.gov/).

Co-immunoprecipitation

WT-CMs cells and CMs overexpressing UNC45B-FLAG were cultured in two 10-cm dishes and harvested using Accutase. Pellets were washed with 1x PBS and resuspended in ice-cold lysis buffer (50 mM Tris-HCl pH 7.5, 1 mM EDTA, 150 mM NaCl, 1% Triton X-100, 10% glycerol, 1 mM PMSF) with protease inhibitor cocktail (Roche). After lysis, cell lysates were centrifuged to remove cell debris. The supernatant was collected and protein concentration was measured using a BCA Protein Assay Kit. Part of the supernatant was transferred to a new tube and served as input for co-immunoprecipitation. For sample pre-cleaning, Mag-Beads (TOOLS, TOPG/A-2) were placed on a magnetic stand and equilibrated with Mag-Beads wash buffer (PBS buffer with 0.02% Tween 20). Cell lysates were mixed with the beads and mouse IgG, and then incubated for 2 h at 4 °C with end-over-end rotation. For sample binding, Anti-FLAG® M2 Magnetic Beads (Sigma, M8823) were used and first equilibrated with FLAG-bead wash buffer (50 mM Tris-HCl pH 7.5, 1 mM EDTA, 150 mM NaCl, 0.5% NP-40, 10% glycerol, 1 mM dithiothreitol, 1 mM PMSF). Next, the pre-cleaned supernatant was incubated with equilibrated FLAG beads for 3 h at 4 °C with end-over-end rotation. Beads were collected and washed with wash buffer. Then, 3x FLAG peptide (30 µg/ml, Sigma-Aldrich) was added, and the mixture was incubated in an orbital shaker at 4 °C for 50 min to elute the immunoprecipitate. SDS-PAGE was used for detection and to separate the FLAG-associated immunoprecipitate. The SDS-PAGE gels were stained with Easy Blue-Plus CBB Stain Reagent (EBL, PEB-021000) for 1 h at RT and washed three times with ddH_2O. Each gel lane was cut into three fragments for detection.

Shotgun proteomic identifications by mass spectrometry (MS)

NanoLC−nanoESi-MS/MS analysis was performed on a Thermo UltiMate 3000 RSLCnano system connected to a Thermo Orbitrap Fusion mass spectrometer (Thermo Fisher Scientific, Bremen, Germany) equipped with a nanospray interface (New Objective, Woburn, MA). Peptide mixtures were loaded onto a 75 µm ID, 25-cm long PepMap C18 column (Thermo Fisher Scientific) packed with 2 µm particles with a pore with of 100 Å, and they were separated over 1 h using a segmented gradient from 5% to 35% solvent B (0.1% formic acid in acetonitrile) at a flow rate of 300 nl/min. Solvent A was 0.1% formic acid in water. The mass spectrometer was

operated in the data-dependent mode. In brief, survey scans of peptide precursors from 350 to 1600 *m*/*z* were performed at 240K resolution with a $2×10^5$ ion count target. Tandem MS was performed using an isolation window of 1.6 Da with the quadrupole, HCD fragmentation with a normalized collision energy of 30, and rapid-scan MS analysis in the ion trap. The MS^2 ion count target was set to $1×10^4$ and the max injection time was 50 ms. Only those precursors with charge state 2–6 were sampled for MS^2. The instrument was run in top speed mode with 3 s cycles. The dynamic exclusion duration was set to 15 s, with a 10 ppm tolerance around the selected precursor and its isotopes. Monoisotopic precursor selection was turned on.

Proteome Discoverer label-free quantification

The MS and tandem MS raw data were processed in Proteome Discoverer (v.2.4.0; Thermo Scientific, Waltham, MA, USA) and searched against the Swiss-Prot protein sequence database with the Mascot server (v.2.7.0; Matrix Science, Boston, MA, USA). Taxonomy was set as *Homo sapiens*. The search criteria used were trypsin digestion, static modification as carbamidomethyl (C), variable modification as oxidation (M), and allowing up to 2 missed cleavages, a mass accuracy of 10 ppm for the parent ion, and 0.6 Da for the fragment ion mass tolerance. Label-free quantification was performed without normalization. Protein ratio was calculated in Proteome Discoverer using a pairwise ratio-based approach. To identify potential protein candidates interacting with UNC45B, the tandem MS data were analyzed by selecting proteins with a unique peptide number >25 and an abundance ratio >1.5.

Quantitative real-time PCR (qRT-PCR)

We used 2 μg of total RNA to synthesize cDNA (Thermo Fisher Scientific, 18080400). Real-time qPCR was performed in a CFX384 (Bio-Rad) machine with Maxima SYBR Green qPCR Master mix (K0252, ThermoFisher). Differences between samples and controls were calculated based on the $2^{-\Delta\Delta CT}$ method and normalized by GAPDH. Statistical significance was determined using a two-tailed Student's t-test ($p < 0.05$). The primers are listed in Table S1.

Transmission electron microscopy (TEM)

hESC-derived CMs were replated on Matrigel-coated Aclar embedding film for 5 days. Cells were fixed with 2.5% glutaraldehyde, 2% paraformaldehyde, 0.1% tannic acid in 0.1 M cacodylate buffer (pH7.2) for 30 min. Cells were then washed three times with 0.2 M sucrose and 0.1% calcium chloride in 0.1 M cacodylate buffer (pH7.2), and then post-fixed in 1% OsO_4 in 0.1 M cacodylate buffer (pH7.2) for 30 min. Cells were washed with ddH_2O three times and stained with 1% uranyl acetate for 30 min. The cells were then washed for a further three times with ddH_2O before being dehydrated via an ethanol gradient (30%, 50%, 70%, 90%, 100%; for 5 min at each concentration). Samples were infiltrated with 1:1, 2:1 (EPON: 100% EtOH) and pure EPON resin. All above-described procedures were performed at RT. Samples

were embedded in EPON at 60 °C for 48 hr. After slicing, images of cell ultrastructures were captured using a Tecnai G2 Spirit TWIN electron microscope (FEI) equipped with a Gatan CCD Camera (794.10.BP2 MultiScan) and acquisition software DigitalMicrograph (Gatan).

Fig. S1

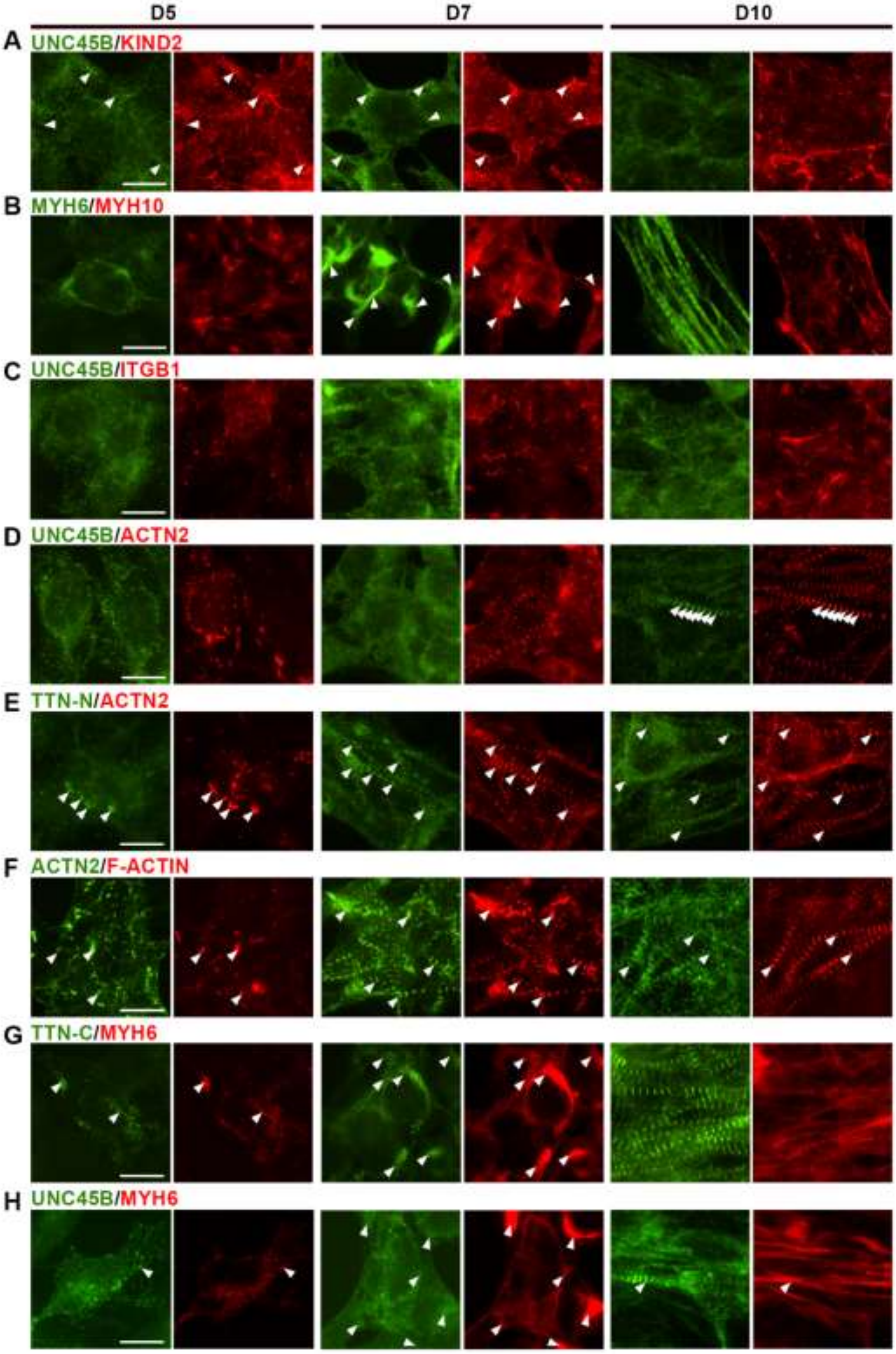
D5
D7
D10
A UNC45B/KIND2
B MYH6/MYH10
C UNC45B/ITGB1
D UNC45B/ACTN2
E TTN-N/ACTN2
F ACTN2/F-ACTIN
G TTN-C/MYH6
H UNC45B/MYH6

Supplemental Figure 1. Unmerged immunofluorescence images of cardiac myofibrillogenesis in human embryonic stem cell-derived cardiomyocytes (hESC-derived CMs). Related to Figure 1.

A-H. Representative unmerged immunofluorescence images of WT-CMs cultured on Matrigel-coated coverslides and harvested at the indicated time-points (D5, D7 and D10). Cells were stained for: (A) UNC45B (Unc-45 Myosin Chaperone B; green) and KIND2 (kindlin-2; red); (B) MYH6 (muscle myosin II; green) and MYH10 (non-muscle myosin IIB; red); (C) UNC45B (green) and ITGB1 (Integrin beta-1; red); (D) UNC45B (green) and ACTN2 (α-actinin; red); (E) TTN-N (N-terminus of titin; green) and ACTN2 (red); (F) ACTN2 (green) and F-ACTIN (red); (G) TTN-C (C-terminus of titin; green) and MYH6 (red); and (H) UNC45B (green) and MYH6 (red). Overlapping fluorescence signals in each panel are indicated by arrowheads. Scale bars: 10 μm.

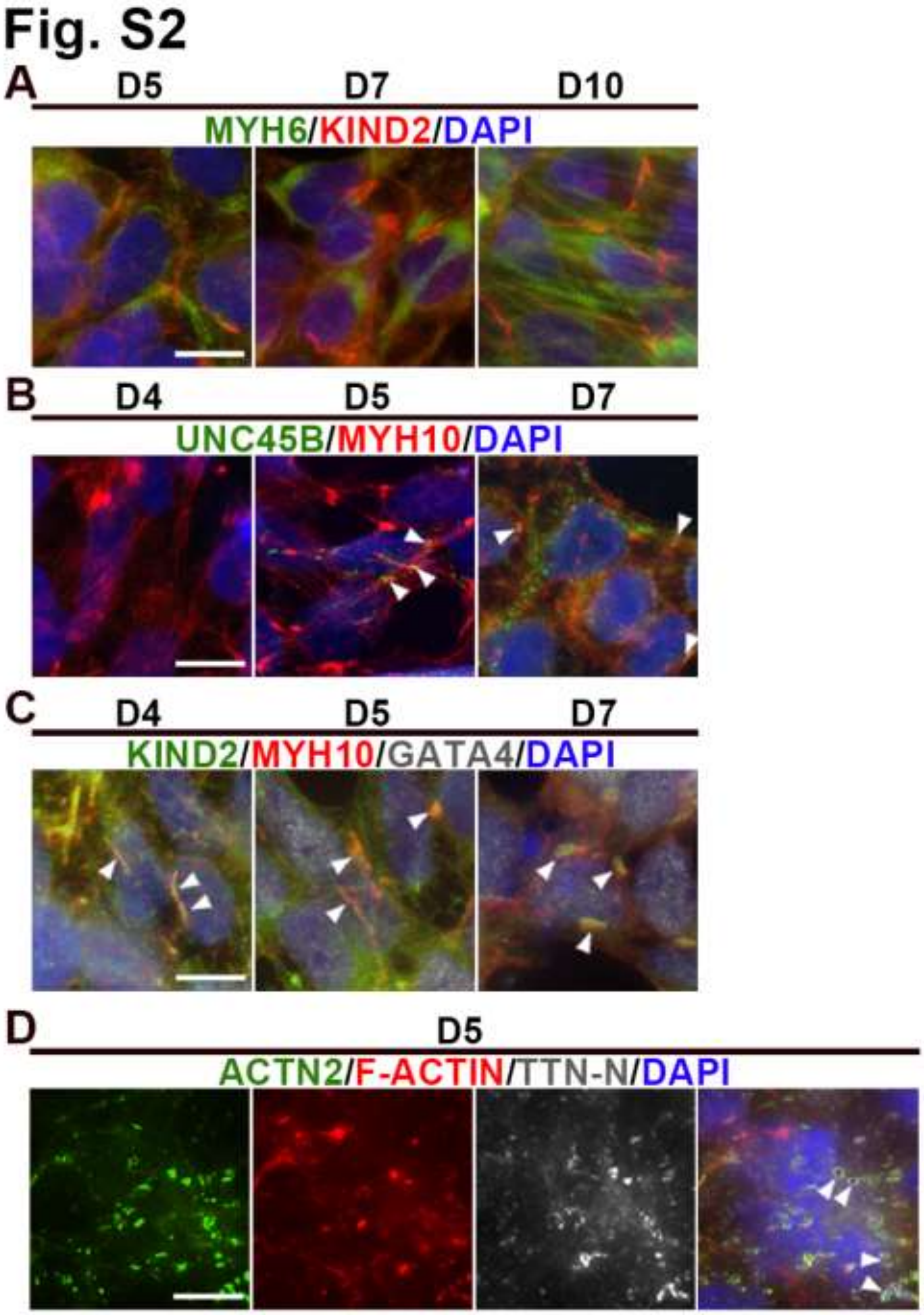

Supplemental Figure 2. Protocostameres are initiated on cardiac differentiation day 4. Related to Figure 1.

A.-**C**. Representative immunofluorescence images of WT-CMs harvested at the indicated time-points. Cells were stained with antibodies for: (A) MYH6 (green) and KIND2 (red); (B) UNC45B (green) and MYH10 (red); or (C) KIND2 (green), MYH10 (red), and GATA4 (white). **D**. Representative immunofluorescence images of WT-CMs harvested at day 5, stained for ACTN2 (green), F-ACTIN (red), and TTN-N (white). Overlapping fluorescence signals in each panel are indicated by arrowheads. Scale bars: 10 μm

Fig. S3

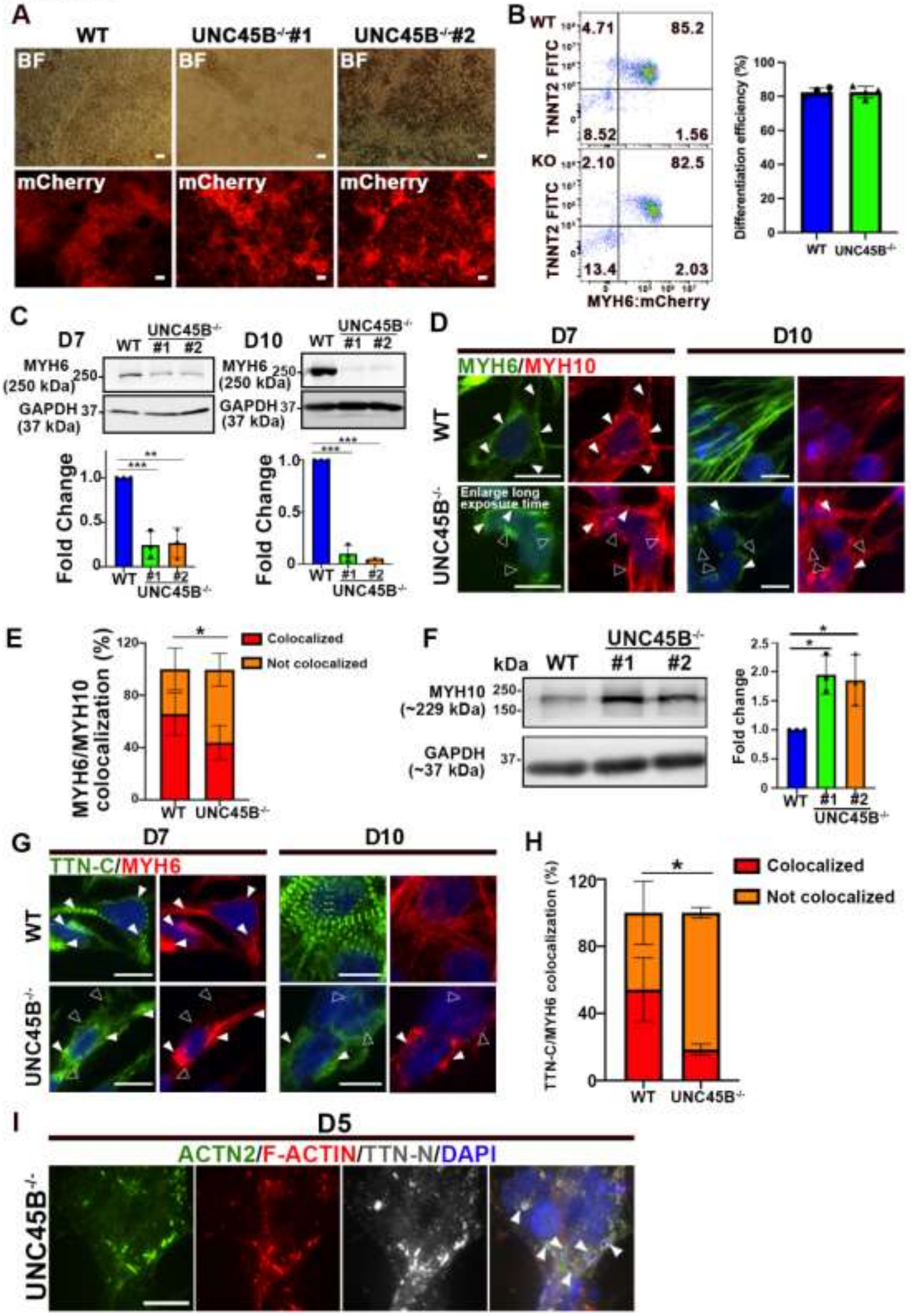
A
WT
UNC45B-/-#1
UNC45B-/-#2
BF
mCherry
B
WT
4.71
85.2
8.52
1.56
KO
2.10
82.5
13.4
2.03
TNNT2 FITC
MYH6:mCherry
Differentiation efficiency (%)
WT
UNC45B-/-
C
D7
D10
UNC45B-/-
WT
#1
#2
MYH6 (250 kDa)
GAPDH (37 kDa)
Fold Change
D
MYH6/MYH10
Enlarge long exposure time
E
MYH6/MYH10 colocalization (%)
Colocalized
Not colocalized
F
kDa
MYH10 (~229 kDa)
GAPDH (~37 kDa)
Fold change
G
TTN-C/MYH6
H
TTN-C/MYH6 colocalization (%)
I
D5
ACTN2/F-ACTIN/TTN-N/DAPI
UNC45B-/-

Supplemental Figure 3. UNC45B$^{-/-}$ hESCs can differentiate into cardiomyocytes. Related to Figure 2 and Figure 3.

A. Images of WT and UNC45B$^{-/-}$ hESC-CM lines acquired under bright-field (upper panels; BF) and fluorescence (bottom panels; mCherry) microscopy at differentiation day 10. Scale bars: 100 μm. **B**. Representative flow cytometry plots of cardiomyocyte differentiation efficiency, as determined by flow cytometry of TNNT2-FITC-conjugated and MYH6:mCherry signal (WT n=4, UNC45B$^{-/-}$ line n=4). Quantification results of flow cytometry analysis (right panel). Error bars represent SD. **C.** Western blots and quantification data for MYH6 signal in day 7 and day 10 WT-CMs and UNC45B$^{-/-}$-CMs. GAPDH served as the loading control. Error bars represent SD. Statistical significance is indicated: *p < 0.05. **p < 0.01. ***p < 0.005. **D**. Representative unmerged immunofluorescence images of WT-CMs and UNC45B$^{-/-}$-CMs cultured on Matrigel-coated coverslides and harvested at the indicated time-points (D7 and D10). Cells were stained for MYH6 (green) and MYH10 (red). Overlapping fluorescence signals in each panel are indicated by white arrowheads. Non-overlapping fluorescence signals in each panel are indicated by empty arrowheads. **E.** Quantification of overlapping fluorescence signals of MYH6 and MYH10. Error bars represent SD. Statistical significance is indicated: *p < 0.05. **F.** Western blots and quantification data for MYH10 signal in WT-CMs and UNC45B$^{-/-}$-CMs. GAPDH served as the loading control. Error bars represent SD. Statistical significance is indicated: *p < 0.05. **G.** Representative unmerged immunofluorescence images of non-replated WT-CMs and UNC45B$^{-/-}$-CMs and harvested at the indicated time-points (D7 and D10). Cells were stained for TTN-C (green) and MYH6 (red). Overlapping fluorescence signals in each panel are indicated by white arrowheads. Non-overlapping fluorescence signals in each panel are indicated by empty arrowheads. **H.** Quantification of overlapping fluorescence signals of MYH6 and MYH10. Error bars represent SD. Statistical significance is indicated: *p < 0.05. **I.** Representative immunofluorescence images of UNC45B$^{-/-}$-CMs harvested at day 5 and stained for ACTN2 (green), F-ACTIN (red), and TTN-N (white). Overlapping fluorescence signals in each panel are indicated by arrowheads. Merged fluorescence images with DAPI staining (blue) are also shown. Scale bars: 10 μm.

Fig. S4

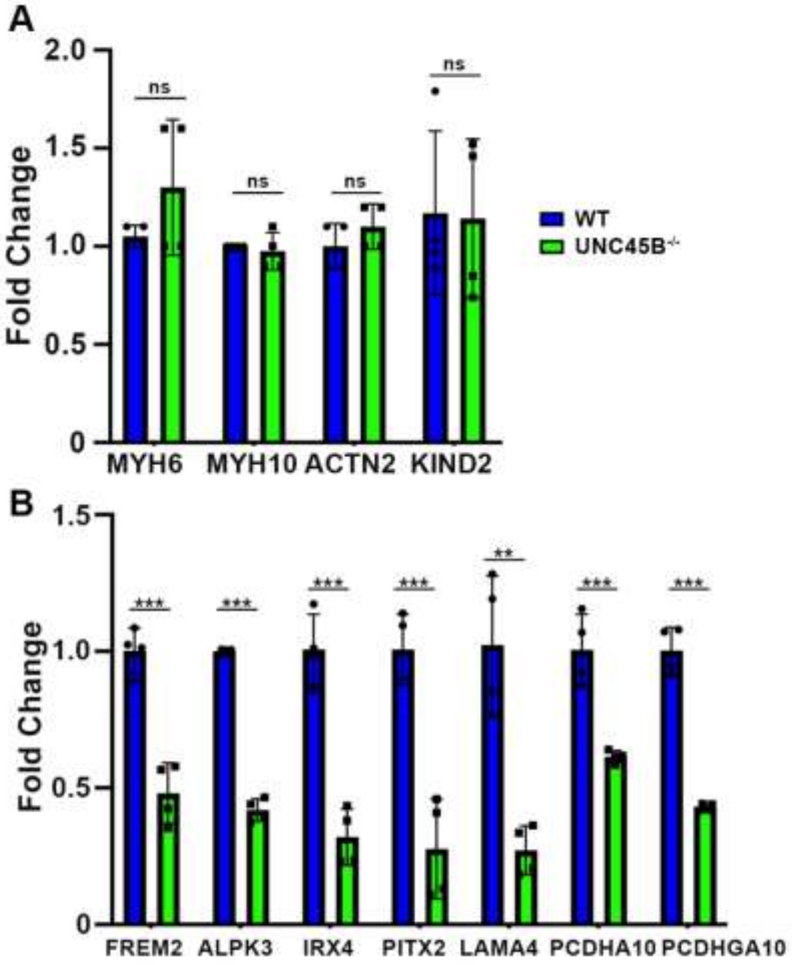

Supplemental Figure 4. The transcript levels of sarcomere markers and cardiomyocyte-related genes in WT-CMs and UNC45B$^{-/-}$-CMs. Related to Figure 4.

A-B. The expression levels of sarcomeric genes (A) and cardiomyocyte-related genes (B) in day 7 WT-CMs and UNC45B$^{-/-}$-CMs.

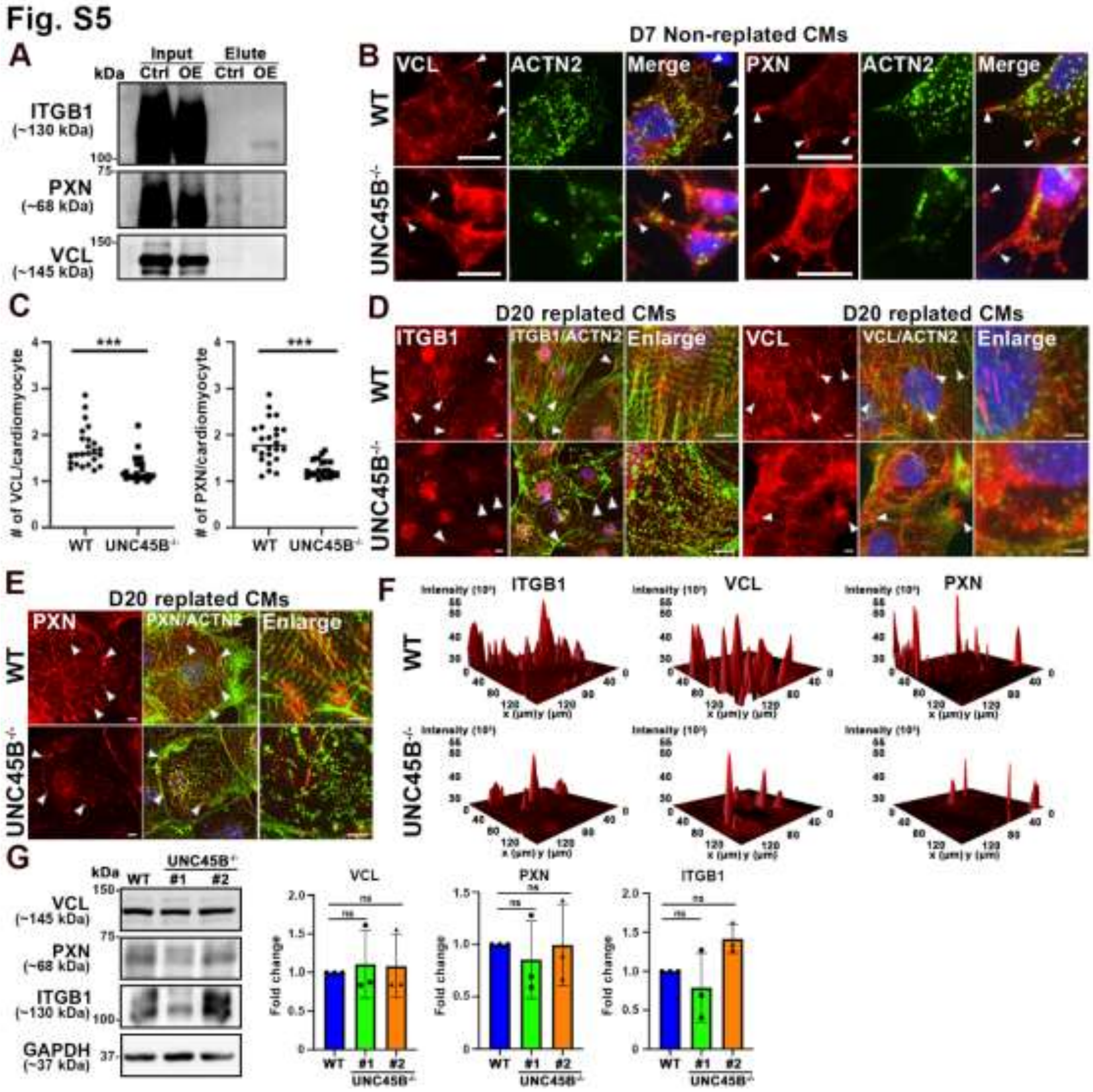

Supplemental Figure 5. UNC45B deletion significantly reduces numbers of protocostamere nucleation sites. Related to Figure 5.

A. Association of UNC45B-FLAG with ITGB1, PXN, and VCL, as observed by co-IP assay followed by Western blot analysis. **B**. Representative immunofluorescence images of WT-CMs and UNC45B$^{-/-}$-CMs harvested at day 7. Cells were stained for antibodies against (b, left panel) VCL (red) and ACTN2 (green) or (right panel) PXN (red) and ACTN2 (green). **C**. Quantification results for numbers of VCL (left) and PXN (right) nucleation sites per cardiomyocyte in WT-CMs and UNC45B$^{-/-}$-CMs (VCL n=24, PXN n=24). Error bars represent SD. Statistical significance is indicated: ***p < 0.001. **D**.-**E**. Representative immunofluorescence images of replated WT-CMs and UNC45B$^{-/-}$-CMs harvested at day 20. Cells were stained for antibodies against (D, left panel) ITGB1 (red) and ACTN2 (green), (D, right panel) VCL (red) and ACTN2 (green), or (E) PXN (red) and ACTN2 (green). Scale bars: 10 µm. **F**. Representative fluorescence intensity 3D surface plots of ITGB1 (left), VCL (middle), and PXN (right) signals for replated WT-CMs (top)

and UNC45B$^{-/-}$-CMs (bottom) harvested at day 20, illustrating protocostamere sites. **G**. Western blots and quantification data for VCL, PXN, and ITGB1 in WT-CMs and UNC45B$^{-/-}$-CMs. GAPDH served as the loading control. Error bars represent SD.

Fig. S6

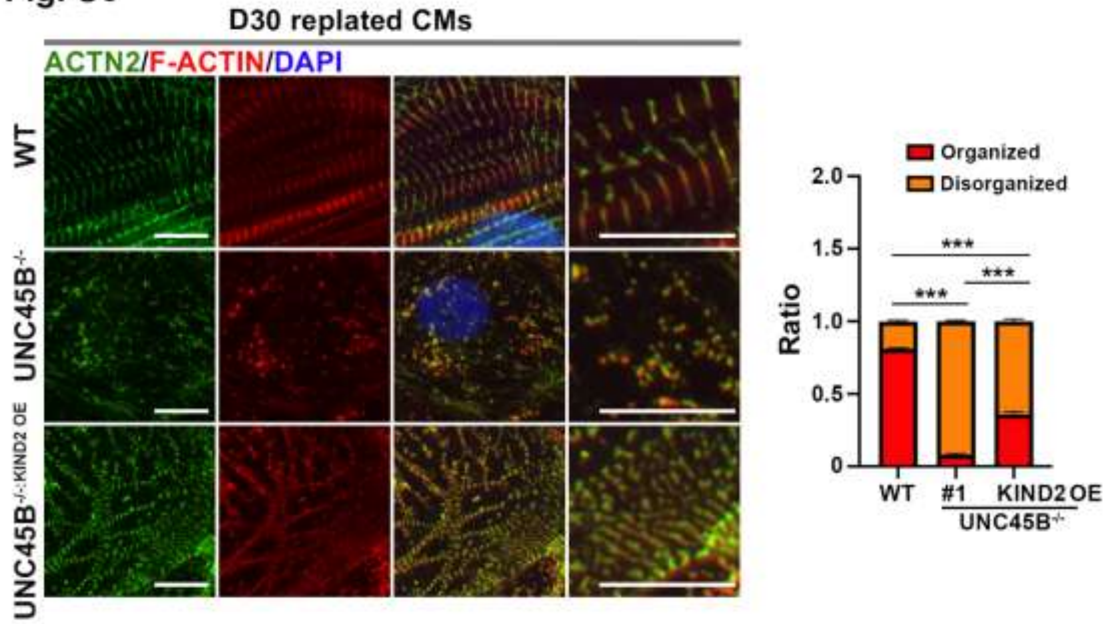

Supplemental Figure 6. ACTN2 accumulation and a failure of F-ACTIN polymerized can be improved by ectopic expression of full-length KIND2 in a UNC45B$^{-/-}$ line. Related to Figure 5.

Left panel. Representative immunofluorescence images of re-plated day 30 WT, UNC45B$^{-/-}$-CMs and UNC45B$^{-/-:KIND2\ OE}$-CMs and stained for the Z-line marker ACTN2 (green) and Actin thin marker F-ACTIN (red). **Right panel.** Rescue efficiency was quantified based on ACTN2 staining. Note that ~35.9±0.98 % of ACTN2 accumulation was rescued in the UNC45B$^{-/-:KIND2\ OE}$-CMs. Results represent data from at least three independent experiments. Scale bars: 10 µm.

Table S1. Primer lists. Related to Figure 2, Figure 6 and Figure 7.

Name of Primer	Sequence (5’ to 3’)
UNC45B sgRNA#1_F	GGCCACGCTTTATCGGAACC
UNC45B sgRNA#1_R	GGTTCCGATAAAGCGTGGCC
UNC45B sgRNA#2_F	CGCCACAAATAGCTACAGCC
UNC45B sgRNA#2_R	GGCTGTAGCTATTTGTGGCG
UNC45B genotype_F	GCTGACCAGACAGAGCTTCTCCTG
UNC45B genotype_R	GAATGAAGCCCTCTGGATCCCGAG
qPCR UNC45B_F	TGACCTCTGCAATTTGCTTG
qPCR UNC45B_R	ATATGGGGAGGGGAAATGAG
qPCR GAPDH_F	GAAGGTGAAGGTCGGAGTC
qPCR GAPDH_R	GAAGATGGTGATGGGATTT
UNC45B OE_1_NheI_F	GCTAGCATGGCAGAGGTGGAAGCGGTAC
UNC45B OE_1_SalI_R	GTCGACCCTTCCGCAAACTGCCTGAGAC
UNC45B OE_2_SalI_F	GTCGACAGAAAAACTGGCCAAACAGTGTCGC
UNC45B OE_2_NotI_R	GCGGCCGCAGACACTGGTTTAATGAAACCATAATCCATG
KIND2 OE_NheI_F	GCTAGCATGGCTCTGGACGGGATAAGGATG
KIND2 OE_NotI_R	GCGGCCGCCACCCAACCACTGGTAAGTTTGTAG
FLAG_EcoRI_R	GAATTCCTATTTATCGTCATCATCTTTGTAGTCCTTG
UNC45B OE_sequence_F	GCCAGAGCCACAGTGATTCTGC
UNC45B OE_sequence_R	CCTTGAGCCACAATGGTGCCTC
pLAS3W sequence	GTTCGGCTTCTGGCGTGTG
pX330 sequence	GGACTATCATATGCTTACCG

Table S2. Ingenuity Canonical Pathway analysis of day 7 RNA sequencing data. Related to Figure 4.

Ingenuity Canonical Pathways	Log (p-value)	z-score
GP6 Signaling Pathway	5.43	-2.111
Human Embryonic Stem Cell Pluripotency	4.23	-1.732
CREB Signaling in Neurons	3.4	-3.13
Intrinsic Prothrombin Activation Pathway	3.39	-2.236
LXR/RXR Activation	3.25	-2.646
Neurovascular Coupling Signaling Pathway	3.07	-2.714
cAMP-mediated signaling	3.01	-1.508
HIF1α Signaling	2.89	-2.53
G-Protein Coupled Receptor Signaling	2.61	-1.528
Breast Cancer Regulation by Stathmin1	2.04	-2.668
S100 Family Signaling Pathway	1.86	-2.236
STAT3 Pathway	1.78	-2
Role of PKR in Interferon Induction and Antiviral Response	1.76	-1.633
Glutamate Receptor Signaling	1.75	-2
Corticotropin Releasing Hormone Signaling	1.55	-1.633
eNOS Signaling	1.5	-2.236
BAG2 Signaling Pathway	1.42	2
Immunogenic Cell Death Signaling Pathway	1.32	2
NOD1/2 Signaling Pathway	1.17	2.449
Synaptic Long Term Depression	1.1	-2.449
IL-15 Production	0.936	-2
Calcium Signaling	0.932	-2.236
White Adipose Tissue Browning Pathway	0.807	-2
Endocannabinoid Neuronal Synapse Pathway	0.726	-2
Semaphorin Neuronal Repulsive Signaling Pathway	0.719	-2
Synaptogenesis Signaling Pathway	0.699	-1.89
FAK Signaling	0.642	-2.524
Phagosome Formation	0.446	-2.309
CLEAR Signaling Pathway	0	2
Chaperone Mediated Autophagy Signaling Pathway	0	2.236
Mitochondrial Dysfunction	0	-2
Phospholipase C Signaling	0	-2
Systemic Lupus Erythematosus In B Cell Signaling Pathway	0	-1.633

Supplemental References

Cong, L., Ran, F.A., Cox, D., Lin, S., Barretto, R., Habib, N., Hsu, P.D., Wu, X., Jiang, W., Marraffini, L.A., and Zhang, F. (2013). Multiplex genome engineering using CRISPR/Cas systems. Science *339*, 819-823.

Lu, S.H., Lee, K.Z., Hsu, P.W., Su, L.Y., Yeh, Y.C., Pan, C.Y., and Tsai, S.Y. (2022). Alternative Splicing Mediated by RNA-Binding Protein RBM24 Facilitates Cardiac Myofibrillogenesis in a Differentiation Stage-Specific Manner. Circ Res *130*, 112-129. 10.1161/circresaha.121.320080.

Ran, F.A., Hsu, P.D., Wright, J., Agarwala, V., Scott, D.A., and Zhang, F. (2013). Genome engineering using the CRISPR-Cas9 system. Nat Protoc *8*, 2281-2308.

Tohyama, S., Hattori, F., Sano, M., Hishiki, T., Nagahata, Y., Matsuura, T., Hashimoto, H., Suzuki, T., Yamashita, H., Satoh, Y., et al. (2013). Distinct Metabolic Flow Enables Large-Scale Purification of Mouse and Human Pluripotent Stem Cell-Derived Cardiomyocytes. Cell Stem Cell *12*, 127-137. https://doi.org/10.1016/j.stem.2012.09.013.

Tsai, S.Y., Ghazizadeh, Z., Wang, H.J., Amin, S., Ortega, F.A., Badieyan, Z.S., Hsu, Z.T., Gordillo, M., Kumar, R., Christini, D.J., et al. (2020). A human embryonic stem cell reporter line for monitoring chemical-induced cardiotoxicity. Cardiovasc Res *116*, 658-670. 10.1093/cvr/cvz148.
